# Construction of an Esophageal Cancer Prognostic Model Based on Mitochondrial Energy Metabolism-Related Genes and Mechanism of INHBA in Promoting Malignant Progression via the Smad2/3 Pathway

**DOI:** 10.3390/biom16091367

**Published:** 2026-09-20

**Authors:** Jinping Li, Yi Yao, Huikai Li, Bozong Shao, Enqiang Linghu

**Affiliations:** Department of Gastroenterology, The First Medical Center of Chinese PLA General Hospital, No.28 Fuxing Road, Beijing 100853, China; lijinping_pla@163.com (J.L.);

**Keywords:** esophageal cancer, mitochondrial energy metabolism, prognostic model, INHBA, epithelial–mesenchymal transition, Smad2/3 signaling pathway

## Abstract

Objective: Esophageal cancer (ESCA) has poor prognosis and lacks reliable biomarkers. This study aimed to construct a mitochondrial energy metabolism-related prognostic model and identify key regulatory genes. Methods: TCGA-ESCA and GSE53625 data were used as training and validation cohorts, respectively. Mitochondrial energy metabolism scores were calculated by ssGSEA. Differential expression analysis and WGCNA identified key genes, followed by consensus clustering, Cox/LASSO regression, enrichment analysis, immune infiltration, TIDE evaluation, and nomogram development. INHBA knockdown or overexpression was performed in KYSE-150 and TE-1 cells to assess malignant phenotypes, mitochondrial function, EMT markers, and Smad2/3 signaling, with SB431542 used for pathway blockade. Results: High mitochondrial energy metabolism scores predicted poorer overall survival. A total of 124 key genes were identified, and three molecular subtypes were established, with Cluster 2 showing the worst prognosis. A four-gene signature comprising COL11A1, INHBA, TNFAIP6, and POSTN stratified patients into high- and low-risk groups in both cohorts. High-risk tumors were enriched in extracellular matrix remodeling, cell adhesion, inflammatory response, hypoxia, angiogenesis, and PI3K-Akt signaling, whereas oxidative phosphorylation was more active in low-risk tumors. The high-risk group had higher TIDE scores, suggesting poorer immunotherapy response. The nomogram incorporating risk group and M stage showed limited discrimination after bootstrap correction. In vitro, INHBA promoted proliferation, migration, invasion, EMT, mitochondrial membrane potential, ATP production, and oxidative phosphorylation. SB431542 partially reversed these effects. Conclusions: This MEMRG-based model predicts ESCA prognosis and immune features. INHBA promotes ESCA progression by activating Smad2/3-mediated EMT and mitochondrial metabolic reprogramming, suggesting its potential as a prognostic biomarker and therapeutic target.

## 1. Introduction

Esophageal cancer (ESCA) remains a major digestive malignancy that poses a serious threat to human health worldwide, and GLOBOCAN 2022 data indicate persistently high incidence and mortality, highlighting a substantial public health burden [1,2]. ESCA mainly comprises esophageal squamous cell carcinoma and esophageal adenocarcinoma, of which esophageal squamous cell carcinoma accounts for a higher proportion in Asia, particularly in the Chinese population, and shows marked molecular differences from esophageal adenocarcinoma [3,4]. Because early symptoms lack specificity, many patients are diagnosed at a locally advanced stage or with metastasis, resulting in unsatisfactory overall treatment outcomes and long-term survival [2]. In recent years, immune checkpoint inhibitors combined with chemotherapy have demonstrated survival benefits in advanced ESCA or esophageal squamous cell carcinoma, and studies such as KEYNOTE-590, CheckMate 648, and ESCORT-1st have driven updates in systemic treatment paradigms for ESCA [5,6,7]. However, a considerable proportion of patients show limited responses to immunotherapy or develop resistance, suggesting that conventional TNM staging and routine pathological indicators do not fully capture tumor heterogeneity and treatment sensitivity [8]. Therefore, identifying novel molecular biomarkers and establishing robust prognostic models is important for risk stratification and individualized treatment in patients with ESCA.

Metabolic reprogramming is a key biological hallmark of cancer, and mitochondrial energy metabolism plays a pivotal role in tumor cell proliferation, invasion, oxidative stress adaptation, and therapeutic resistance [9]. Specifically, mitochondria not only generate adenosine triphosphate (ATP) through the tricarboxylic acid cycle and oxidative phosphorylation, but also participate in reactive oxygen species (ROS) production, maintenance of mitochondrial membrane potential, regulation of cell death, and biosynthesis of metabolic intermediates [9]. Although cancer metabolism research has long focused on the Warburg effect, accumulating evidence indicates that some tumor cells still depend on or reactivate oxidative phosphorylation to meet the energy demands of rapid proliferation and metastasis [9,10]. In esophageal squamous cell carcinoma, mitochondrial energy metabolism has been reported to correlate with an immunosuppressive tumor microenvironment (TME) and a poor prognosis, suggesting its potential role in tumor progression and immune evasion [11]. Moreover, prognostic signatures constructed from mitochondrial energy metabolism-related genes (MEMRGs) can effectively predict survival outcomes in patients with esophageal squamous cell carcinoma, indicating the potential of such genes as tools for prognostic stratification [12]. Therefore, systematically dissecting the molecular heterogeneity of ESCA from a mitochondrial energy metabolism perspective may facilitate the identification of novel prognostic biomarkers and potential therapeutic targets.

Malignant progression of ESCA is jointly shaped not only by tumor-intrinsic alterations but also by extracellular matrix remodeling, inflammatory responses, and the TME [13,14,15]. In particular, extracellular matrix-related genes may influence prognosis in esophageal squamous cell carcinoma by regulating cell adhesion, matrix deposition, tumor cell migration, and immune infiltration, and they are closely associated with therapeutic responses [16]. The tumor immune dysfunction and exclusion (TIDE) algorithm predicts immunotherapy response by evaluating features of T cell dysfunction and T cell exclusion, thereby providing an important transcriptome-level tool for inferring tumor immune evasion [8]. INHBA is a member of the TGF-β superfamily that encodes the βA subunit of Activin A, and previous studies indicate that it is upregulated in esophageal squamous cell carcinoma and is associated with poor prognosis [17]. As a key molecule within the Activin/TGF-β signaling axis, INHBA may regulate tumor cell migration, invasion, epithelial–mesenchymal transition (EMT), and extracellular matrix remodeling through the Smad2/3 pathway [18,19,20]. EMT is typically characterized by downregulation of E-cadherin and upregulation of mesenchymal markers such as N-cadherin, Vimentin, and Snail, and it represents an important mechanism promoting tumor metastasis and therapeutic resistance [21,22]. However, whether INHBA simultaneously modulates EMT and metabolic reprogramming in esophageal squamous cell carcinoma via the Smad2/3 pathway remains insufficiently validated.

Building on this background, this study aimed to investigate molecular features and prognostic differences in ESCA based on MEMRGs by systematically analyzing The Cancer Genome Atlas (TCGA)-ESCA cohort and the Gene Expression Omnibus (GEO) GSE53625 cohort. First, we calculated the mitochondrial energy metabolism score (MEMS) using single-sample gene set enrichment analysis (ssGSEA) and, together with differential expression analysis and weighted gene co-expression network analysis (WGCNA), screened key genes to further construct molecular subtyping and a prognostic risk model. Next, we evaluated the associations between the risk score and clinicopathological features, immune infiltration, TIDE-predicted immunotherapy response, and patient survival, thereby clarifying the clinical predictive value of the prognostic model. Furthermore, we selected the key gene INHBA for in vitro functional validation and assessed its effects on esophageal squamous cell carcinoma cell proliferation, migration, invasion, three-dimensional matrix invasion, mitochondrial membrane potential, ATP production, ROS levels, and oxidative phosphorylation capacity. Finally, by examining EMT markers and Smad2/3 pathway activation and conducting mechanistic validation using the ALK5 inhibitor SB431542, we sought to elucidate the molecular mechanism by which INHBA promotes malignant progression and metabolic reprogramming in esophageal squamous cell carcinoma. Collectively, this study may provide new theoretical and experimental support for prognosis evaluation, molecular subtyping, immunotherapy response prediction, and INHBA-targeted intervention in ESCA.

## 2. Materials and Methods

### 2.1. Data Acquisition

In this study, the TPM expression profile data (ESCA group: 185 cases; control group: 13 cases) and clinical data of the ESCA dataset were downloaded from the TCGA (https://portal.gdc.cancer.gov/; accessed on 21 April 2026)) database using the TCGAbiolinks package (version 2.27.2) (Table 1). Because this study required overall survival (OS) and overall survival time (OS. Time) information for ESCA patients, only “01A” tumor samples with available OS status and OS. Time > 0 were retained; therefore, 149 ESCA tumor samples (without paired adjacent samples) were included in the analysis. In addition, “11A” normal control samples were retained, yielding 13 samples for analysis. For TPM expression profiles, only protein-coding genes were kept, and low-expression genes with zero expression in at least half of the samples were removed; consequently, 18,063 genes were retained for subsequent analyses.

To characterize somatic mutations in ESCA, “Masked Somatic Mutation” data were downloaded from the TCGA database via the TCGAbiolinks package for ESCA patients, including 185 cases. Subsequently, mutation profiles were visualized using the maftools package (version 2.12.0) in R.

To evaluate copy number variation (CNV) in ESCA, “Masked Copy Number Segment” data were downloaded from the TCGA database through the TCGAbiolinks package as CNV data for ESCA patients, comprising 173 cases, and the CNV data were stratified into high- and low-risk groups according to risk classification. Next, GISTIC 2.0 analyses were conducted for CNV segments in the high- and low-risk groups, respectively, using GenePattern. During analysis, the confidence level was set to 0.99 (corresponding to a *q*-value threshold of 0.01), while all other parameters were set to GISTIC 2.0’s default settings.

To provide external validation, the ESCA-related dataset GSE53625 was downloaded from the GEO (https://www.ncbi.nlm.nih.gov/geo/, accessed on 21 April 2026) database using the GEOquery package in R. The dataset was derived from Homo sapiens and generated on the GPL18109 platform, containing 179 tumor samples, all of which were included in the analysis (no paired adjacent samples). For expression data in GSE53625, between-sample normalization was performed using the normalizeBetweenArrays function in the limma package in R to mitigate batch effects. Subsequently, boxplots were used to display gene expression distributions before and after correction.

To comprehensively define MEMRGs, we compiled MEMRGs from studies by Zhang et al. [11] and Meng et al. [23], yielding a total of 1697 MEMRGs. Therefore, we intersected this gene set with the TCGA-ESCA expression profile, yielding 1649 MEMRGs for subsequent analyses (Supplementary Material S2).

### 2.2. Calculation of MEMS

To quantify mitochondrial energy metabolism at the individual-sample level in the TCGA-ESCA expression profile, we used GSVA (v1.46.0) in R and computed MEMS for each sample based on MEMRGs using the ssGSEA algorithm applied to the gene expression matrix.

### 2.3. Differential Gene Analysis Related to Mitochondrial Energy Metabolism

To stratify samples by mitochondrial energy metabolism, we first derived MEMS for each sample as described above and then used survival (v3.5-7) and survminer (v0.4.9) in R to identify the optimal cut-off value via the surv_cutpoint function by integrating survival status and survival time. Subsequently, samples were categorized into the high-MEMS and low-MEMS groups based on MEMS.

To quantify how gene expression levels were associated with mitochondrial energy metabolism, differential expression analysis was conducted between the high MEMS and low MEMS groups in the TCGA-ESCA cohort using the limma package (v3.52.4) in R. Therefore, genes were filtered using the thresholds of adjusted *p*-value (adjusted *p*-value) < 0.01 and |log fold change (FC)| > 1, where genes with logFC > 1 and adjusted *p*-value < 0.01 were defined as upregulated mitochondrial energy metabolism-related differentially expressed genes (DEGs), whereas genes with logFC < −1 and adjusted *p*-value < 0.01 were defined as downregulated mitochondrial energy metabolism-related DEGs. The differential analysis results were visualized as a volcano plot and a heatmap generated with ggplot2 (v3.4.4) and pheatmap (v1.0.12) in R, respectively.

To characterize the expression patterns of key genes across clinical groupings, boxplots were generated using ggplot2 in R to compare key gene expression between the ESCA and normal control groups, and between the high and low MEMS groups. In addition, intergroup differences were evaluated using the Wilcoxon rank-sum test. Pearson correlation coefficients among key genes were computed with the corrplot package in R and visualized as a correlation heatmap.

### 2.4. Mutation and CNV Analyses of Key Genes

To delineate the genomic alteration landscape of key genes in the TCGA-ESCA dataset, somatic mutation frequencies were analyzed and visualized using the maftools package in R. In addition, based on TCGA-ESCA CNV data, the frequencies of copy number amplification and deletion for key genes were summarized and presented using a bar chart.

### 2.5. WGCNA

WGCNA was conducted to identify co-expressed gene modules, interrogate the relationships between gene networks and phenotypes, and characterize hub genes within the network. Using the TCGA-ESCA expression profile data as input, the WGCNA package (v1.72-1) in R was applied, and the soft-thresholding power was calculated with the pickSoftThreshold function. Specifically, the optimal power (β) was determined by requiring the scale-free topology fit index (R^2^) to reach 0.9; therefore, a scale-free co-expression network was constructed, followed by the generation of the topological overlap matrix and hierarchical clustering. With 300 set as the minimum module size, gene modules were identified using a dynamic tree-cutting approach, yielding eight modules in total. The Pearson correlation coefficients between each module eigengene (ME) and the MEMS grouping trait were then calculated, with larger absolute coefficients indicating stronger module-trait associations. The two modules with the strongest associations with MEMS grouping were selected, and the genes within them were identified as key module genes significantly associated with the trait. Finally, key genes were obtained as the intersection of the DEGs from the high MEMS versus low MEMS comparison and the key module genes, and a Venn diagram was generated using the venn package (v1.11) in R to display the results.

### 2.6. Construction of Molecular Subtyping Based on MEMS-Related Genes

Consensus clustering is a method to determine the plausible number of clusters and their memberships within a dataset. To better distinguish molecular subtypes of ESCA, the ConsensusClusterPlus package (v1.60.0) in R was used to perform consensus clustering of TCGA-ESCA expression profile data using key genes. During this procedure, the number of clusters was set from 2 to 8, and 80% of the total samples were resampled for 1000 iterations; the clustering algorithm was set to PAM (Partitioning Around Medoids), and Spearman correlation distance was used as the distance metric. For the molecular subtypes obtained from consensus clustering, principal component analysis (PCA) was performed to evaluate subtype separability, and a PCA scatter plot was generated using the ggplot2 package in R.

### 2.7. Construction of a Risk Model Based on MEMS-Related Genes

Least absolute shrinkage and selection operator (LASSO) regression is widely used for prognostic model development because it introduces an L1 regularization penalty into linear regression to shrink coefficients, thereby reducing overfitting and improving generalizability. To evaluate the predictive value of key genes for survival in patients with ESCA, the TCGA-ESCA dataset was used as the training cohort, and prognostic genes were further screened using univariate Cox regression, LASSO regression, and multivariate Cox regression analyses to construct a prognostic model. First, a univariate Cox regression analysis was conducted to assess the association between each key gene’s expression and OS; genes with *p* < 0.05 were retained. Subsequently, the LASSO algorithm was applied to mitigate multicollinearity and select informative variables from the univariate Cox regression results. To identify more accurate independent prognostic factors (prognostic signature genes), multivariate Cox regression analysis was then performed, and the final set of genes was determined using stepwise regression. Finally, the risk score was calculated based on the expression levels of the selected genes and their multivariate Cox regression coefficients, using the following formula:

$$\mathrm{riskScore}=\sum_{i}\mathrm{Coefficient}\left({\mathrm{gene}}_{i}\right)\times \mathrm{mRNA}\;\mathrm{Expression}({\mathrm{gene}}_{i})$$

To stratify patients by the optimal risk score threshold, the optimal cut-off value for risk score grouping was determined using the surv_cutpoint function in R, and patients in the TCGA-ESCA cohort were subsequently classified into high- and low-risk groups. The R survival package was then used to conduct Kaplan–Meier analysis and the log-rank test to evaluate OS in the TCGA cohort. In addition, risk scatter plots were generated using the R ggplot2 package to illustrate the proportions of patients in the high- and low-risk groups, as well as survival time and survival status. Furthermore, time-dependent receiver operating characteristic (ROC) curves were applied to assess survival-prediction performance, and the timeROC package (v0.4) in R was used to calculate the area under the curve (AUC) as a measure of prognostic prediction accuracy.

To externally validate the model, risk scores for each sample were calculated in the GSE53625 expression profile dataset, serving as the validation set, using the formula described above, and risk stratification of the validation cohort was performed using the cut-off value derived from the training set. The R survival package was used to perform Kaplan–Meier analysis and the log-rank test to evaluate OS in the GSE53625 cohort. In parallel, the R ggplot2 package was used to generate risk scatter plots, and the R timeROC package was used to compute the AUC to quantify the accuracy of prognostic prediction in the external validation dataset.

### 2.8. DEG Analysis of the MEMS Model

To identify genes associated with the MEMS risk model, differential expression analysis between the high- and low-risk groups defined in the TCGA-ESCA expression profile dataset was conducted using the limma package in R (v3.52.4). Genes were screened using adjusted *p*-value < 0.01 and |logFC| > 0.5 as thresholds; specifically, genes with logFC > 0.5 and adjusted *p*-value < 0.01 were defined as upregulated DEGs, whereas genes with logFC < −0.5 and adjusted *p*-value < 0.01 were defined as downregulated DEGs. The differential analysis results were visualized using volcano plots generated with the R ggplot2 package, and heatmaps were produced using the pheatmap package.

### 2.9. Functional Enrichment Analysis and Gene Set Enrichment Analysis (GSEA)

Gene Ontology (GO) analysis is a commonly used method for large-scale functional enrichment studies, encompassing biological process (BP), molecular function (MF), and cellular component (CC). The Kyoto Encyclopedia of Genes and Genomes (KEGG) is a widely used database that stores information on genomes, biological pathways, diseases, and drugs. We performed GO annotation and KEGG pathway enrichment analyses for DEGs using the clusterProfiler package (version 4.7.1.003) in R, with term selection criteria of adjusted *p*-value < 0.05 and *q*-value < 0.05, and *p*-values were adjusted using the Benjamini–Hochberg (BH) method.

To further investigate differences in BP between the high- and low-risk groups, GSEA was performed using gene expression profiles from TCGA-ESCA patients. GSEA is a computational method that evaluates whether a predefined gene set shows statistically significant differences between two biological states and is commonly used to estimate changes in pathway and BP activities within expression datasets. We downloaded the “h.all.v7.5.1.symbols” Hallmark gene sets from the MSigDB database and performed GSEA for the TCGA-ESCA high- and low-risk groups. An adjusted *p*-value < 0.05 was considered statistically significant.

### 2.10. Construction of the Protein–Protein Interaction (PPI) Network and Identification of Hub Genes

PPI networks consist of proteins connected through their interactions and are involved in BPs, such as signal transduction, regulation of gene expression, energy and material metabolism, and cell cycle control. Systematic analysis of protein interactions in biological systems is of great significance for elucidating signal transduction and metabolic regulatory mechanisms under special physiological states, such as diseases. The STRING (https://string-db.org/) database is a protein interaction search platform that integrates evidence from experimentally validated data, text mining, multi-database integration, and bioinformatic predictions. We used the STRING database to construct a PPI network for DEGs between the high- and low-risk groups, with a minimum interaction confidence threshold of 0.4 (medium confidence). The PPI results were exported from the STRING database and visualized in Cytoscape, and the CytoHubba plugin was used to identify hub genes in the PPI network by ranking nodes by degree. The corrplot package in R was used to calculate Pearson correlation coefficients among hub genes and between hub genes and the risk score, and to generate a correlation heatmap. Finally, the GOSemSim package in R was used to conduct gene functional semantic similarity analysis (Friends analysis) of hub genes to assess the extent of functional relatedness among them.

### 2.11. Construction of an mRNA-miRNA Regulatory Network

The miRTarBase database (https://mirtarbase.cuhk.edu.cn/, accessed on 21 April 2026) is dedicated to curating microRNA–mRNA targeting relationships (microRNA–target interactions; MTI) with experimental evidence. To characterize the regulatory relationships between hub genes and miRNAs, we downloaded miRNA–mRNA interaction information from the miRTarBase database. Based on the hub genes identified from the PPI analysis, we queried miRTarBase to retrieve experimentally validated targeting miRNAs and constructed an miRNA–mRNA regulatory network. Cytoscape (version 3.10.1) was used to visualize the miRNA–mRNA regulatory network.

### 2.12. Tumor Immune Infiltration and Correlation Analysis

The TME is a complex, integrated system composed primarily of tumor tissues, surrounding immune and inflammatory cells, cancer-associated fibroblasts, stromal tissues, and diverse cytokines and chemokines. The analysis of immune cell infiltration in cancer tissues provides significant guidance for disease research and the prediction of treatment prognosis.

The immune-related gene set was obtained from the study by Charoentong et al. [24], which includes 782 genes and 28 immune cell types, spanning various human immune cell subtypes, including activated CD8 T cells, activated dendritic cells, macrophages, natural killer T cells, and regulatory T cells. The GSVA package (version 1.46.0) in R was used to estimate the infiltration levels of each immune cell subtype in the TCGA-ESCA expression profiles using the ssGSEA algorithm. Boxplots were generated using the ggplot2 package in R to illustrate the differences in the infiltration levels of each immune cell subtype between the high- and low-risk groups.

The ESTIMATE package (version 1.0.13) in R was used to evaluate tumor immune activity. The ESTIMATE method quantitatively evaluates the immune infiltration level in tumor samples based on gene expression profiles and calculates the Immune Score, Stromal Score (reflecting the abundance of stromal components in tumor tissues), and ESTIMATE Score (used to infer tumor purity) for each tumor sample.

To characterize immune associations, we calculated Pearson correlation coefficients between hub gene expression levels and infiltration scores for each immune cell subtype, and then visualized these correlations as a heatmap using the ggplot2 package in R. In addition, we calculated Pearson correlation coefficients between hub genes and immune-related genes and displayed the results as a heatmap using the pheatmap package in R. We also used the Wilcoxon rank-sum test to compare the expression levels of immune-related genes between the high- and low-risk groups.

### 2.13. Construction of a Clinical Prognostic Model Based on the MEMS Model

A nomogram is a visualization tool derived from multivariable regression analysis that assigns scales to independent variables and sums the corresponding points to estimate the probability of an event. To evaluate the individualized prognostic performance of risk scores combined with clinicopathological characteristics in ESCA patients, we analyzed the TCGA-ESCA dataset by first screening clinicopathological features significantly associated with OS (*p* < 0.05) using univariable Cox regression and then performing multivariable Cox regression to identify independent prognostic factors. Variables that remained statistically significant in the multivariable Cox regression (*p* < 0.05) were integrated with the risk score to construct a clinical predictive nomogram using the rms package (version 6.7-1) in R. Finally, calibration curves were generated by comparing nomogram-predicted versus observed 1-, 2-, and 3-year OS to assess the predictive performance of the nomogram. Cox models used complete cases for the variables included in each analysis, and the effective sample size and number of deaths were reported for every model. The final two-variable model was internally validated using 1000 bootstrap resamples; Harrell C-index and calibration slope were used to quantify optimism and potential overfitting.

### 2.14. TIDE-Based Evaluation of Immunotherapy Response

To predict potential responsiveness to immune checkpoint inhibitors, we applied the TIDE algorithm to samples in the TCGA-ESCA dataset. Specifically, each sample’s gene expression profile was submitted to the TIDE online platform to obtain TIDE scores and the predicted immunotherapy response status. The Wilcoxon rank-sum test was used to compare TIDE scores between the high- and low-risk groups, and ROC curves were plotted, and AUC values were calculated using the timeROC package in R to evaluate the risk score’s sensitivity in discriminating immunotherapy response. In addition, a proportional bar chart of immunotherapy response status between the high- and low-risk groups was generated using the ggplot2 package in R.

### 2.15. Cell Line Sources, Culture Conditions, and Quality Control

To ensure the authenticity and biosafety of the experimental materials, the human esophageal squamous cell carcinoma cell lines KYSE-150 and KYSE-450 were purchased from Procell (Wuhan, China; KYSE-150: Cat# CL-0638; KYSE-450: Cat# CL-0990), and the TE-1 cell line was obtained from Ubigene (Guangzhou, China; Cat# YC-B086). All cell lines were accompanied by vendor-provided STR authentication reports to confirm cell identity. In addition, mycoplasma contamination was assessed using a mycoplasma PCR detection kit (Beyotime, Shanghai, China; Cat# C0301S), and all results were negative.

According to the manufacturers’ recommended protocols, KYSE-150 and KYSE-450 were maintained in a 1:1 mixture of RPMI-1640 medium (Gibco, Thermo Fisher Scientific, Waltham, MA, USA; Cat# 11875093) and Ham’s F-12 medium (Gibco, Thermo Fisher Scientific, Waltham, MA, USA; Cat# 11765054) as the basal medium, whereas TE-1 was cultured in RPMI-1640 medium (Gibco, Thermo Fisher Scientific, Waltham, MA, USA; Cat# 11875093). All media were supplemented with 10% fetal bovine serum (FBS; Gibco, Thermo Fisher Scientific, Waltham, MA, USA; Cat# 10099141) and 1% penicillin-streptomycin (Gibco, Thermo Fisher Scientific, Waltham, MA, USA; Cat# 15140122), and cells were incubated at 37 °C in a humidified atmosphere containing 5% CO_2_. For passaging, cells were dissociated with 0.25% Trypsin-EDTA (Gibco, Thermo Fisher Scientific, Waltham, MA, USA; Cat# 25200056). The cells used in all experiments were in the logarithmic growth phase.

### 2.16. Construction of Cell Models with INHBA Knockdown and Overexpression

Based on the regression coefficients, correlation with the risk score, the magnitude of differential expression between high- and low-risk groups, and previous literature evidence for the four key genes in the prognostic model (COL11A1, INHBA, TNFAIP6, and POSTN), INHBA was selected as the subject for subsequent in vitro functional and mechanistic validation after comprehensive screening.

First, quantitative real-time polymerase chain reaction (qRT-PCR) and Western blot (WB) were used to detect the basal expression level of INHBA in KYSE-150, KYSE-450, and TE-1 cells. Based on the detection results, cells with high INHBA expression were selected for knockdown experiments, and cells with low INHBA expression were selected for overexpression experiments. INHBA siRNA (target sequences are provided in Supplementary Material S1) and negative control siRNA were synthesized by GenePharma (Shanghai, China); the INHBA overexpression plasmid and empty vector control plasmid were constructed by GeneChem (Shanghai, China). Cell transfection was performed using Lipofectamine™ 3000 transfection reagent (Invitrogen, Thermo Fisher Scientific, USA, Cat# L3000008) according to the manufacturer’s instructions. Cells were seeded in 6-well plates 24 h before transfection to reach 60–70% confluence on the day of transfection; 2.5 μg of plasmid DNA or 50 nM siRNA was used per well, and P3000 Reagent and Lipofectamine 3000 were added as instructed. At 48 h post-transfection, RNA and proteins were extracted, and qRT-PCR and WB were used to verify the efficiency of INHBA intervention.

### 2.17. RNA Extraction and qRT-PCR Analysis

Total RNA of cells in each group was extracted using TRIzol™ Reagent (Invitrogen, Thermo Fisher Scientific, USA, Cat# 15596026), and reverse transcription was conducted with the PrimeScript™ RT Reagent Kit with gDNA Eraser (Takara Bio, Shiga, Japan, Cat# RR047A). qRT-PCR was performed using TB Green^®^ Premix Ex Taq™ II (Takara Bio, Japan, Cat# RR820A). The reaction mixture was prepared according to the kit instructions with a total volume of 20 μL, including 10 μL of 2× TB Green Premix, 0.4 μL of forward primer, 0.4 μL of reverse primer, 2 μL of cDNA template, and nuclease-free water up to the volume. The amplification conditions were: 95 °C for 30 s, followed by 40 cycles of 95 °C for 5 s and 60 °C for 30 s; melt-curve analysis was performed after amplification. GAPDH was used as the internal reference gene, and relative expression was calculated using the 2^−ΔΔCt^ method. Primers were synthesized by Sangon Biotech (Shanghai, China), and primer sequences are provided in Supplementary Material S1. Each group had three technical replicates, and the experiments were independently repeated three times.

### 2.18. Protein Extraction and WB Analysis

Cells from each group were lysed using strong RIPA lysis buffer containing protease and phosphatase inhibitors (Beyotime, Shanghai, China, Cat# P0013B). Protein concentration was determined using an enhanced BCA protein assay kit (Beyotime, Shanghai, China, Cat# P0010). Equal amounts of protein (20–30 μg/lane) were separated by SDS-PAGE and transferred onto PVDF membranes; after blocking with 5% non-fat milk at room temperature for 1 h, the membranes were incubated with primary antibodies at 4 °C overnight. Subsequently, the membranes were washed three times with TBST, incubated with HRP-conjugated secondary antibodies at room temperature for 1 h, and visualized using the BeyoECL Plus chemiluminescence kit (Beyotime, Shanghai, China, Cat# P0018M). ImageJ (version 1.54t) was used for densitometric analysis.

The primary antibodies used in this study included INHBA (Abcam, Cambridge, UK; Cat# ab97705; 1:1000), E-cadherin (Cell Signaling Technology, Danvers, MA, USA; Cat# 3195; 1:1000), N-cadherin (CST; Cat# 13116; 1:1000), Vimentin (CST; Cat# 5741; 1:1000), Snail (CST; Cat# 3879; 1:1000), Phospho-Smad2 (Ser465/467) (CST; Cat# 3108; 1:1000), Smad2/3 (CST; Cat# 8685; 1:1000), Phospho-Smad3 (Ser423/425) (CST; Cat# 9520; 1:1000), and GAPDH (CST; Cat# 5174; 1:5000). The secondary antibody was HRP-conjugated anti-rabbit IgG (ZSGB-BIO, Beijing, China; Cat# ZB-2301).

### 2.19. Cell Counting Kit-8 (CCK-8) Cell Proliferation Assay

Cell proliferation capacity was evaluated using CCK-8 (Beyotime, Shanghai, China; Cat# C0037). Specifically, cells from each group were seeded into 96-well plates at 2 × 10^3^ to 3 × 10^3^ cells per well, with five replicate wells established per group. Then, 10 μL of CCK-8 working solution was added to each well at 0, 24, 48, 72, and 96 h after seeding; after incubation for 2 h at 37 °C in the dark, absorbance at 450 nm (OD_450_) was measured using a microplate reader, and growth curves were generated. The experiment was independently repeated three times.

### 2.20. Colony Formation Assay

Colony-forming capacity was assessed by seeding cells from each group into 6-well plates at 500–1000 cells per well. Subsequently, cells were cultured under standard conditions for 10 to 14 d, with medium replaced every 3 d. When visible colonies had formed, colonies were fixed with 4% paraformaldehyde for 15 min and stained with 0.1% crystal violet for 20 min. After gentle washing with phosphate-buffered saline (PBS) and air-drying, images were captured. Colonies containing ≥50 cells were counted, thereby enabling comparisons of colony formation capacity across groups. The experiment was independently repeated three times.

### 2.21. Wound Healing Assay

Cells were seeded into 6-well plates and, upon reaching 90–100% confluence, a vertical scratch was created using a sterile 200 μL pipette tip. After gently rinsing with PBS to remove detached cells, serum-free medium was added for further incubation, and images were captured at 0 h and 24 h. The scratch width was measured using ImageJ software, and the healing rate was calculated using the following formula:

Healing rate = (scratch width at 0 h–scratch width at 24 h)/scratch width at 0 h × 100%.

In each group, at least three fields were analyzed, and the experiment was independently repeated three times.

### 2.22. Transwell Migration and Invasion Assays

To quantify cell motility, migration and invasion assays were conducted using Corning Transwell 24-well inserts with an 8.0 μm pore polycarbonate membrane (Corning Inc., Corning, NY, USA, Cat# 3422). For the invasion assay, the inserts were pre-coated with Matrigel Basement Membrane Matrix (Corning Inc., Corning, NY, USA, Cat# 356234), diluted 1:8, and incubated at 37 °C for 30 min to form a gelled matrix layer. After resuspension in serum-free medium, cells from each group were seeded into the upper chamber at 5 × 10^4^ cells/well (migration) or 1 × 10^5^ cells/well (invasion), whereas the lower chamber was filled with 600 μL complete medium supplemented with 10% FBS. After 24 h of incubation, non-migrated cells on the upper surface were gently removed with a cotton swab; cells on the lower surface were then fixed with 4% paraformaldehyde for 15 min and stained with 0.1% crystal violet for 20 min. Subsequently, five random microscopic fields were counted, and the mean value was used as the readout for each insert. All experiments were independently repeated three times.

### 2.23. Three-Dimensional Collagen Invasion Assay

To model invasion within a three-dimensional extracellular matrix context, a three-dimensional matrix was established using type I collagen. Rat tail collagen I was purchased from Corning (USA, Cat# 354236). On ice, collagen, 10× PBS, sterile water, and 0.1 mol/L NaOH were mixed according to the manufacturer’s instructions and neutralized to pH 7.2–7.4 to obtain a collagen solution at a final concentration of 2.0 mg/mL; subsequently, 400 μL was added to each well of a 24-well plate and incubated at 37 °C for 30 min to allow gelation. Cells from each group were then seeded onto the collagen surface at 5 × 10^4^ cells/well and cultured for 72 h. Z-stack images were acquired using an inverted microscope, and the invasion distance into the collagen matrix and the number of invading cells were quantified to evaluate the invasive capacity of cells in a three-dimensional extracellular matrix environment. All experiments were independently repeated three times.

### 2.24. Intracellular ATP Content Measurement

To assess cellular bioenergetic status, intracellular ATP levels were measured using an ATP assay kit (Beyotime, Shanghai, China, Cat# S0026). Briefly, cells from each group were lysed according to the manufacturer’s instructions and incubated with the luciferase working solution, after which chemiluminescence was measured on a microplate luminometer. The ATP content was normalized to the total protein concentration. Technical variation was controlled by using three parallel wells per group, and the entire experiment was independently repeated three times.

### 2.25. Measurement of Mitochondrial Membrane Potential

Mitochondrial membrane potential alterations were evaluated using a mitochondrial membrane potential assay kit (JC-1) (Beyotime, Shanghai, China; Cat# C2006). After the JC-1 working solution was prepared according to the manufacturer’s instructions, cells from each group were incubated in the dark at 37 °C for 20 min. Subsequently, the cells were washed twice with JC-1 staining buffer. Red fluorescence (JC-1 aggregates, high membrane potential) and green fluorescence (JC-1 monomers, low membrane potential) intensities were captured with a fluorescence microscope, and the red/green fluorescence ratio was calculated to reflect changes in mitochondrial membrane potential. The experiment was independently repeated three times.

### 2.26. Measurement of Intracellular ROS Levels

Total intracellular ROS levels were assessed using an ROS assay kit (Beyotime, Shanghai, China; Cat# S0033M). Cells in each group were incubated with DCFH-DA at a final concentration of 10 μM in the dark at 37 °C for 20 min. Subsequently, the cells were washed three times with serum-free medium to remove probes that had not entered the cells. Fluorescence intensity was recorded by fluorescence microscopy. The experiment was independently repeated three times.

### 2.27. Seahorse Cell Metabolic Analysis

Mitochondrial metabolic function was evaluated using the Agilent Seahorse XF Cell Mito Stress Test Kit (Agilent Technologies, Santa Clara, CA, USA, Cat# 103015-100). One day before measurement, cells were seeded into Seahorse-specific microplates to achieve appropriate confluence on the day of the assay. The medium was then replaced with Seahorse XF base medium, and the plate was equilibrated for 1 h at 37 °C in a CO_2_-free incubator. Subsequently, oligomycin (1.5 μM), FCCP (1.0 μM), and rotenone/antimycin A (0.5 μM) were sequentially injected according to the manufacturer’s instructions to quantify basal respiration, ATP-linked respiration, maximal respiration, and spare respiratory capacity. All readouts were normalized to cell number. Experiments were independently repeated three times.

### 2.28. Smad2/3 Signaling Pathway Blockade Assays

To determine whether the biological effects mediated by INHBA depended on the Smad2/3 signaling pathway, the ALK5 inhibitor SB431542 (Selleck Chemicals, Houston, TX, USA, Cat# S1067) was administered to INHBA-overexpressing cells. SB431542 was used at a working concentration of 10 μM, and cells were pretreated for 2 h before subsequent functional assays and protein analyses; in contrast, control cells received an equal volume of DMSO. After treatment, Transwell invasion assays, WB, ATP content measurement, JC-1 mitochondrial membrane potential assessment, and Seahorse oxygen consumption rate (OCR) analysis were conducted to indicate whether INHBA-related phenotypes were reversed upon Smad2/3 signaling blockade.

### 2.29. Detection of Secreted Activin A, Activin A Rescue/Neutralization, and Smad2/3 siRNA Validation

To measure changes in secreted Activin A after INHBA modulation, KYSE-150 cells were transfected with si-NC or si-INHBA, and TE-1 cells were transfected with Vector or oe-INHBA. At 48 h after transfection, the medium was replaced with serum-free medium for an additional 24 h. Cell culture supernatants were collected and centrifuged at 1000× *g* for 10 min to remove cell debris. Activin A concentrations in the supernatants were measured using a Human/Mouse/Rat Activin A Quantikine ELISA Kit (R&D Systems, Bio-Techne, USA; Cat# DAC00B) according to the manufacturer’s instructions. Absorbance was measured at 450 nm, and Activin A concentrations were calculated from the standard curve.

To evaluate whether the Activin A axis contributed to INHBA-associated phenotypes, si-INHBA-transfected KYSE-150 cells were treated with recombinant human Activin A (R&D Systems, Bio-Techne, USA; Cat# 338-AC; 50 ng/mL), whereas oe-INHBA-transfected TE-1 cells were treated with an Activin A neutralizing antibody (R&D Systems, Bio-Techne, USA; Cat# AF338; 10 μg/mL). After 24 h of treatment, Transwell migration/invasion assays and WB analysis were performed as described above to assess cell motility, invasion, EMT markers, and Smad2/3 phosphorylation.

To further validate the pathway specificity, two independent siRNAs targeting Smad2/3 (si-Smad2/3-1 and si-Smad2/3-2) and the corresponding negative control were transfected into oe-INHBA TE-1 cells. The siRNA transfection procedure, reagent amounts, and transfection duration were the same as those described above. At 48 h after transfection, qRT-PCR and WB were used to confirm Smad2/3 knockdown efficiency and changes in p-Smad2/p-Smad3 levels. WB and Transwell migration/invasion assays were subsequently performed to assess whether Smad2/3 inhibition altered oe-INHBA-associated EMT and migratory/invasive phenotypes.

### 2.30. Migration Assays Under Proliferation-Controlled Conditions

To exclude the possibility that migration differences were mainly driven by short-term changes in cell proliferation, wound-healing and Transwell migration assays were repeated under proliferation-controlled conditions. For the wound-healing assay, scratches were generated when cells reached 90–100% confluence, and cells were then cultured in low-serum medium for 24 h. For the Transwell migration assay, cell seeding density, incubation time, fixation, staining, and counting procedures were the same as those described above. CCK-8 assays were performed in parallel within the same experimental time window to monitor cell viability/proliferation status. Wound closure rate and migrated cell number were used as the main motility readouts. Experiments were independently repeated three times.

### 2.31. Chemosensitivity and IC_50_ Analysis

To evaluate the effect of INHBA knockdown on chemosensitivity in ESCC cells, si-NC- and si-INHBA-transfected cells were seeded into 96-well plates and treated with serial concentrations of cisplatin (Selleck Chemicals, Houston, TX, USA; Cat# S1166), paclitaxel (Selleck Chemicals, Houston, TX, USA; Cat# S1150), 5-fluorouracil (5-FU; Selleck Chemicals, Houston, TX, USA; Cat# S1209), or docetaxel (Selleck Chemicals, Houston, TX, USA; Cat# S1148) after cell attachment. After 48 h of drug exposure, cell viability was measured using the CCK-8 assay, and relative cell viability was normalized to the untreated control. Dose–response curves and IC_50_ values were obtained by nonlinear regression fitting. At least three replicate wells were included for each condition, and the experiments were independently repeated three times.

### 2.32. Activin A Secretion, Rescue/Neutralization, and Smad2/3 siRNA Validation Assays

Secreted Activin A levels in the culture supernatant were measured by ELISA after INHBA knockdown in KYSE-150 cells and INHBA overexpression in TE-1 cells. For Activin A-axis rescue or blockade experiments, KYSE-150 cells were transfected with si-NC, si-INHBA, or si-INHBA followed by recombinant Activin A treatment, whereas TE-1 cells were transfected with Vector or oe-INHBA and then treated with an Activin A-neutralizing/blocking reagent. Transwell migration/invasion assays and WB analyses of EMT- and Smad2/3-related proteins were performed to evaluate whether modulation of the Activin A axis altered INHBA-associated phenotypes. To further verify pathway specificity, two independent siRNAs targeting Smad2/3 (si-Smad2/3-1 and si-Smad2/3-2) were transfected into oe-INHBA TE-1 cells using the same siRNA-transfection procedure described above, followed by qRT-PCR, WB, and Transwell assays.

### 2.33. Proliferation-Controlled Migration Assays

To exclude the possibility that migration differences were driven mainly by short-term changes in cell growth, wound-healing and Transwell migration assays were repeated under proliferation-controlled conditions. Cell viability/proliferation status was monitored in parallel during the same experimental time window. Wound closure rate and migrated cell number were used as the primary motility readouts.

### 2.34. Chemotherapy Sensitivity and IC_50_ Analysis

Chemotherapy sensitivity was assessed by treating si-NC and si-INHBA-transfected esophageal cancer cells with concentration gradients of Cisplatin, Paclitaxel, 5-FU, and Docetaxel. Relative cell viability was measured after drug treatment and used to generate dose–response curves. IC_50_ values were calculated from nonlinear dose–response fitting, and the experiment was independently repeated three times.

### 2.35. Ethics Statement and Biosafety

This study exclusively conducted in vitro experiments using commercially available human ESCA cell lines and, therefore, did not involve recruitment of human participants, collection of human tissue or blood samples, or animal experiments, and it was not subject to human research ethics review or animal ethics review. All experiments were performed in compliance with institutional laboratory biosafety regulations.

### 2.36. Statistical Analysis

All bioinformatics data processing and analyses in this study were conducted using R software (version 4.2.2). For comparisons of continuous variables between two groups, an independent Student’s *t*-test was used to assess statistical significance for normally distributed variables, whereas a Mann–Whitney U test (i.e., the Wilcoxon rank-sum test) was used for non-normally distributed variables. A chi-square test or Fisher’s exact test was performed to compare categorical variables between two groups. The survival package in R was used for survival analyses; Kaplan–Meier curves were generated to visualize survival differences, and the log-rank test was applied to evaluate the significance of differences in survival time between two patient groups. Both univariate and multivariate Cox regression analyses were conducted using the survival package in R, and LASSO regression was performed using the glmnet package in R (version 4.1-8).

All in vitro experiments were independently repeated at least three times. Quantitative data were presented as mean ± SD. An independent-samples *t*-test was used for comparisons between two groups, whereas one-way ANOVA was used for comparisons among multiple groups, followed by Tukey’s test for post hoc analyses. A *p*-value < 0.05 was considered statistically significant. Statistical analyses were completed using GraphPad Prism 10.2.1, and all *p*-values were derived from two-sided tests.

## 3. Results

### 3.1. Prognostic Analysis of MEMS and Identification of Key Genes in ESCA Based on the TCGA-ESCA Dataset

The overall workflow of the bioinformatics analyses in this study is shown in Figure S1. To evaluate the potential impact of mitochondrial energy metabolism on ESCA development, we downloaded gene expression profiles from the TCGA database for the training set and the ESCA-related GSE53625 dataset from GEO for the validation set. Box plots were used to display the gene expression distributions in GSE53625 before correction (Figure S2A) and after correction (Figure S2B), indicating that the uncorrected data exhibited inter-sample variability (Figure S2A); however, after normalization and correction with the limma package, the expression trends across samples became consistent and were suitable for subsequent analyses (Figure S2B).

**Figure 1:**
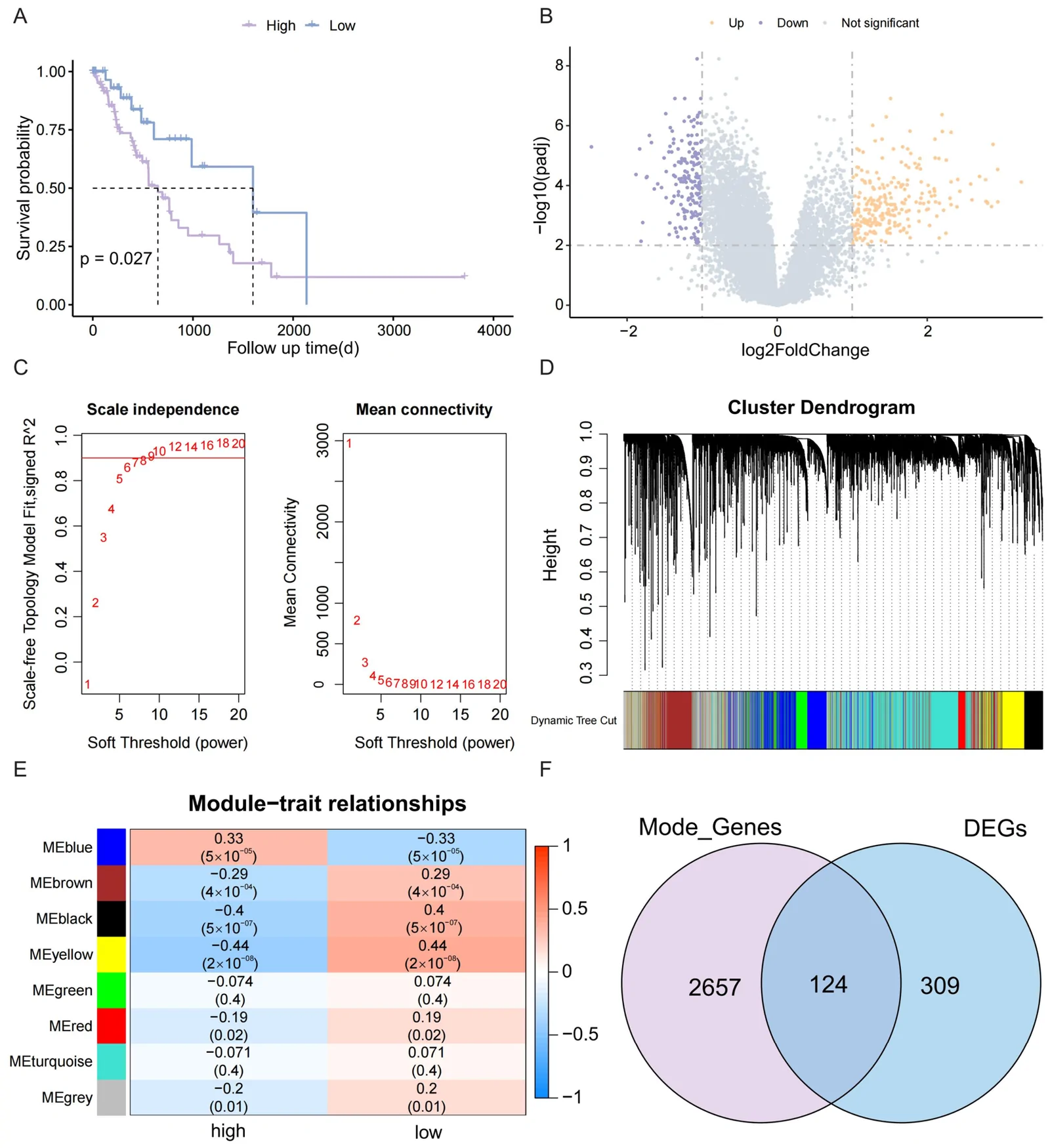
Prognostic analysis related to mitochondrial energy metabolism and screening of key genes based on the TCGA-ESCA dataset. Note: (**A**) Kaplan–Meier survival curves after grouping by MEMS; *p*-values were obtained by the log-rank test. (**B**) Volcano plot of DEGs between the high-MEMS and low-MEMS groups; the *x*-axis shows log_2_FC and the *y*-axis shows −log_10_(adjusted *p*-value); red dots indicate significantly upregulated genes, blue dots indicate significantly downregulated genes, and gray dots indicate genes without significant differences. (**C**) WGCNA soft-threshold selection plots; the left panel shows the scale-free topology fit index (R^2^) and the right panel shows mean connectivity; the red dashed line indicates R^2^ = 0.9. (**D**) Gene hierarchical clustering dendrogram and dynamic tree-cut module assignment; different colors represent different gene modules. (**E**) Heatmap of module–trait correlations; in each cell, the value at the top is the Pearson correlation coefficient and the value in parentheses below is the *p*-value; red indicates positive correlation and blue indicates negative correlation. (**F**) Venn diagram of the intersection between DEGs and WGCNA module hub genes; the intersection represents 124 key genes. TCGA: The Cancer Genome Atlas; ESCA: Esophageal Cancer; MEMS: Mitochondrial Energy Metabolism Score; WGCNA: Weighted Gene Co-Expression Network Analysis; DEGs: Differentially Expressed Genes.

To elucidate the association between mitochondrial energy metabolism and ESCA, we leveraged the TCGA-ESCA cohort and used the ssGSEA algorithm to calculate the MEMS for each ESCA patient, thereby quantifying mitochondrial energy metabolism activity. Based on the optimal MEMS cut-off value (3.730404), patients in the TCGA-ESCA dataset were stratified into high-MEMS and low-MEMS groups. Kaplan–Meier survival analysis indicated that, compared with the high-MEMS group, the low-MEMS group exhibited a relatively favorable prognosis (Log-rank *p* = 0.027; Figure 1A).

To identify key genes associated with mitochondrial energy metabolism in ESCA, we performed differential expression analysis of gene expression profiles between the high-MEMS and low-MEMS groups using the limma package. In total, 433 DEGs were identified in ESCA, including 247 significantly upregulated genes and 186 significantly downregulated genes (Supplementary Material S3; Figure 1B). Meanwhile, to define biologically meaningful gene modules and further prioritize genes closely associated with MEMS, we constructed a weighted gene co-expression network. The optimal soft-thresholding power was selected by requiring a scale-free topology fit index (R^2^) of 0.9 (Figure 1C); therefore, a scale-free co-expression network was established (Figure 1D), yielding eight gene modules (Figure 1E). As shown in Figure 1E, the two modules most strongly correlated with MEMS stratification were the yellow module (cor = −0.44, *p* = 2 × 10^−8^) and the blue module (cor = −0.4, *p* = 5 × 10^−7^), suggesting a close association with MEMS in ESCA patients. We then combined the genes from these two modules, yielding 2781 module key genes (Supplementary Material S4). By intersecting the DEGs with the module key genes, we identified 124 genes as the key genes related to mitochondrial energy metabolism in ESCA (Supplementary Material S5), which were visualized using a Venn diagram (Figure 1F).

### 3.2. Key Gene Analysis and Construction of Molecular Subtyping for MEMS-Related Genes

**Figure 2:**
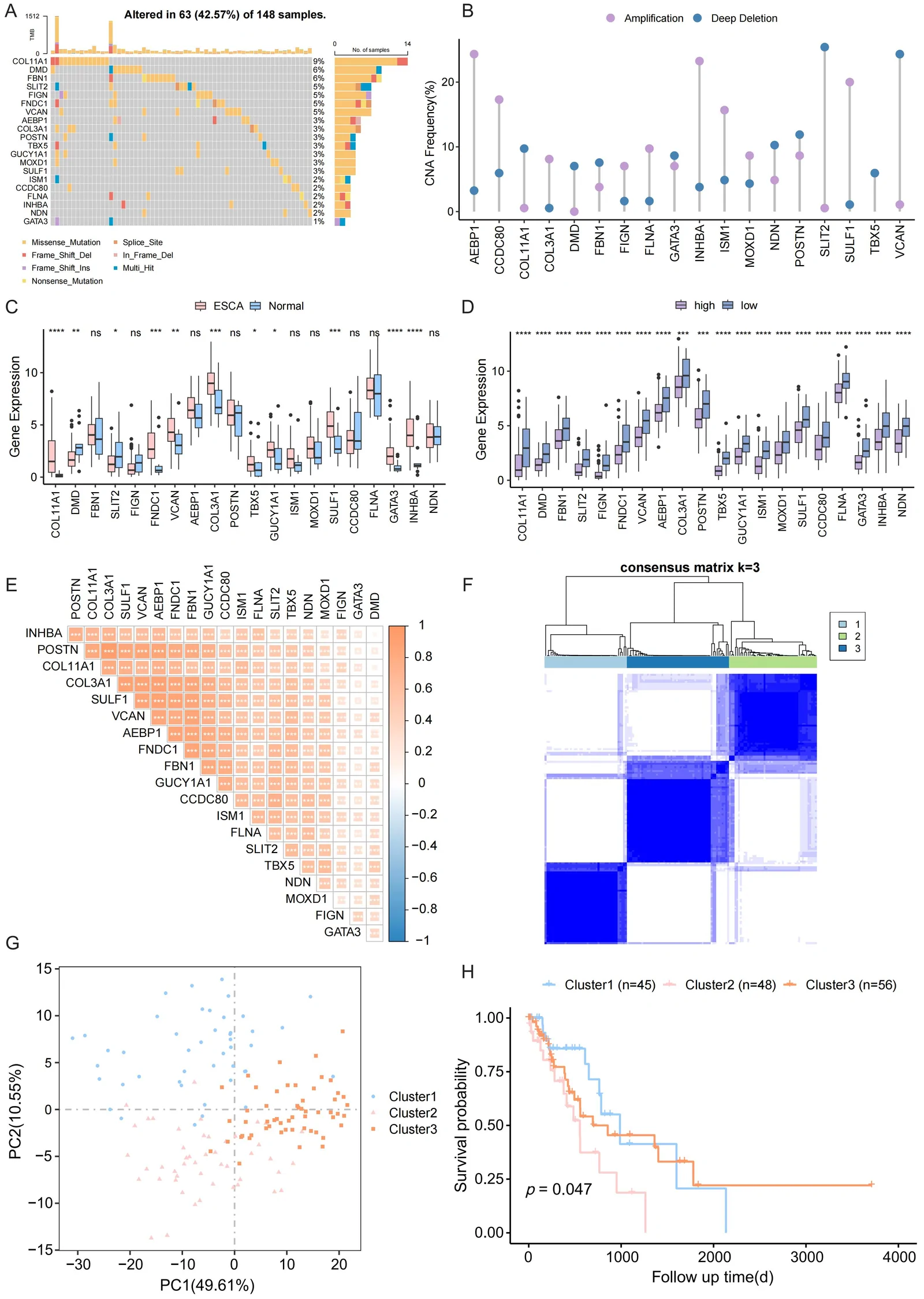
Key gene analysis and construction of MEMS-related molecular subtypes. Note: (**A**) Waterfall plot of somatic mutations in key genes; the bar plot at the top indicates the mutation burden of each sample, the bar plot on the right indicates the mutation frequency of each gene, and different colors represent different mutation types. (**B**) Frequency distribution of copy number alterations in key genes; blue indicates deep deletion and orange indicates amplification. (**C**) Boxplots comparing the expression of the top 20 key genes between ESCA tissues and normal control tissues; between-group comparisons were performed using the Wilcoxon rank-sum test. (**D**) Boxplots comparing the expression of the top 20 key genes between the high-MEMS group and the low-MEMS group; between-group comparisons were performed using the Wilcoxon rank-sum test. (**E**) Pearson correlation heatmap of the top 20 key genes; color intensity indicates the magnitude of the correlation coefficient, red indicates positive correlation, and blue indicates negative correlation. (**F**) Consensus matrix plot of consensus clustering at k = 3; darker color indicates higher clustering consensus among samples. (**G**) PCA scatter plot of the three molecular subtypes; different colors represent different subtypes. (**H**) Kaplan–Meier survival curves for different molecular subtypes; the *p*-value was obtained by the log-rank test. MEMS: Mitochondrial Energy Metabolism Score; ESCA: Esophageal Cancer; PCA: Principal Component Analysis; TCGA: The Cancer Genome Atlas. * *p* < 0.05, ** *p* < 0.01, *** *p* < 0.001, **** *p* < 0.0001; ns indicates no statistically significant difference.

To characterize the impact of key genes on ESCA, we first used the maftools package to profile somatic mutations of key genes in the TCGA-ESCA cohort and displayed the top 20 key genes with the highest mutation frequencies (Figure 2A). The results indicated that key genes were mutated in 63 samples, with an overall mutation frequency of 42.57%, and individual gene mutation rates ranging from 1% to 9%; notably, COL11A1 showed the highest mutation frequency (9%). Furthermore, by analyzing the CNV of key genes in ESCA (top 20 key genes) (Figure 2B), we found that most samples exhibited copy-number alterations, which were predominantly copy-number losses; for example, SLIT2 and VCAN showed relatively high deletion frequencies (20–30%), whereas copy-number gains were less common.

To characterize the overall expression patterns of key genes in ESCA patients, we compared the expression differences of the top 20 key genes between the ESCA and normal control groups (Figure 2C) and between the high MEMS and low MEMS groups (Figure 2D). The results indicated that more than 50% of the key genes differed significantly between the ESCA and normal control groups (*p* < 0.05); specifically, genes such as FNDC1 and COL3A1 were expressed at significantly higher levels in the ESCA group than in the normal control group (*p* < 0.001; Figure 2C), whereas DMD and SLIT2 were expressed at significantly lower levels in the ESCA group (*p* < 0.05; Figure 2C). In addition, all key genes showed significant differences between the high MEMS and low MEMS groups (*p* < 0.01), and most genes were expressed at significantly lower levels in the low MEMS group than in the high MEMS group; for example, CDH11 was expressed at a significantly higher level in the low MEMS group than in the high MEMS group (*p* < 0.001; Figure 2D). We then conducted a Pearson correlation analysis of the top 20 key genes (Figure 2E); the correlation results for all genes are provided in Supplementary Material S6. Overall, key genes generally exhibited significant positive correlations with each other; for example, COL11A1 was significantly and positively correlated with POSTN (cor = 0.837, *p* = 2.936 × 10^−40^).

Based on the above findings, key genes can be used to distinguish samples with different disease states. Therefore, we performed unsupervised clustering of all patient samples using a consensus clustering algorithm based on the expression profiles of 124 key genes. After evaluating different numbers of clusters (Figure 2F), k = 3 yielded the optimal clustering performance, and samples were consequently classified into three molecular subtyping groups: Cluster 1 (*n* = 45), Cluster 2 (*n* = 48), and Cluster 3 (*n* = 56). PCA indicated good separability among the three subtypes (Figure 2G). Furthermore, Kaplan–Meier survival analysis showed significant differences in OS among the three subtypes (Log-rank *p* = 0.047; Figure 2H), with Cluster 2 exhibiting the poorest prognosis, which further verified the biological significance of the clustering results.

### 3.3. Construction and Validation of the MEMS Risk Model in the TCGA-ESCA Dataset

**Figure 3:**
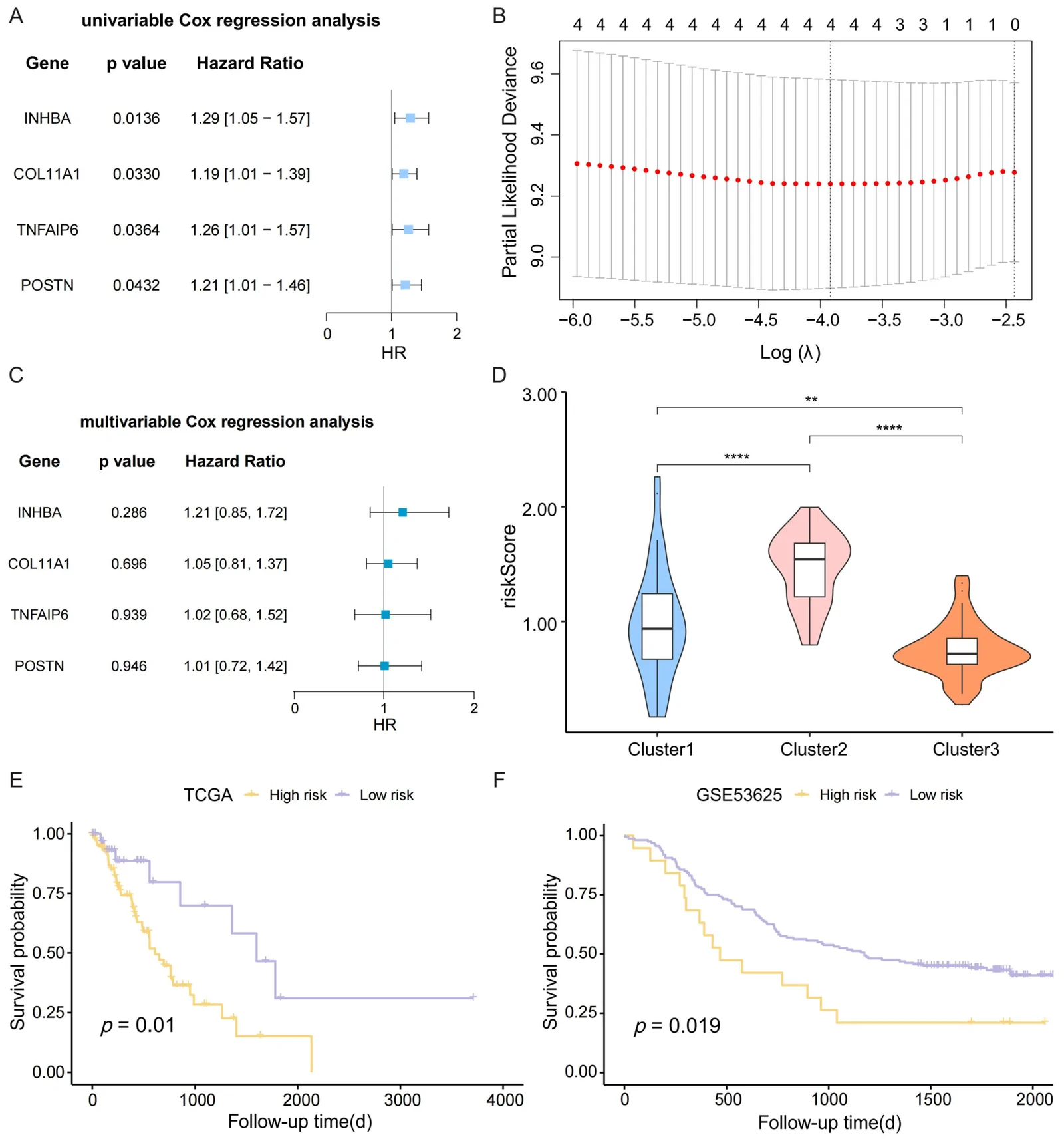
Construction of the MEMS risk model in the TCGA-ESCA dataset. Note: (**A**) Forest plot of univariate Cox regression analysis showing the hazard ratios (HRs) and 95% confidence intervals (CIs) of four key genes significantly associated with prognosis. (**B**) Ten-fold cross-validation plot of LASSO regression, where the red dashed line indicates the optimal penalty parameter (lambda), and the numbers above indicate the corresponding number of genes with non-zero coefficients. (**C**) Forest plot of multivariate Cox regression analysis showing the HRs and 95% CIs of the four genes ultimately included in the model. (**D**) Violin plot comparing risk scores among different MEMS molecular subtypes, with intergroup differences assessed by the Wilcoxon rank-sum test. (**E**) Kaplan–Meier survival curves for high- and low-risk groups in the TCGA cohort, with *p*-values obtained by the log-rank test. (**F**) Kaplan–Meier survival curves for high- and low-risk groups in the GSE53625 validation cohort, with *p*-values obtained by the log-rank test. MEMS: Mitochondrial Energy Metabolism Score; LASSO: Least Absolute Shrinkage and Selection Operator; HR: Hazard Ratio; TCGA: The Cancer Genome Atlas; ESCA: Esophageal Cancer. ** *p* < 0.01, **** *p* < 0.0001.

To quantify the impact of key genes on prognosis in ESCA patients, we constructed a risk score model by integrating the expression levels of 124 key genes. First, we screened the 124 key genes in the TCGA-ESCA dataset using univariate Cox regression and identified four genes significantly associated with prognosis (COL11A1, INHBA, TNFAIP6, and POSTN; *p* < 0.05; Figure 3A). We then applied LASSO regression to eliminate multicollinearity and determined the optimal penalty parameter (lambda) via 10-fold cross-validation, ultimately retaining 4 genes (Figure 3B). Next, these four genes were incorporated into a multivariate Cox regression analysis, and stepwise regression was used to select the optimal combination; finally, the 4 genes (COL11A1, INHBA, TNFAIP6, and POSTN) were used to construct the prognostic risk score model (Figure 3C), with the risk score formula as follows:

$$\mathrm{RiskScore}=\left(0.19\times \mathrm{INHBA}\;\mathrm{expression}\;\mathrm{level}\right)+\left(0.05\times \mathrm{COL}11A1\;\mathrm{expression}\;\mathrm{level}\right)\;+\left(0.02\times \mathrm{TNFAIP}6\;\mathrm{expression}\;\mathrm{level}\right)+\left(0.01\times \mathrm{POSTN}\;\mathrm{expression}\;\mathrm{level}\right)$$

To validate the model’s effectiveness, we compared risk score differences across MEMS molecular subtyping groups. The results indicated that risk scores differed significantly among the three subtypes, with the poorest-prognosis Cluster 2 showing higher risk scores than Clusters 1 and 3 (*p* < 0.001; Figure 3D), suggesting good concordance between the risk score and prognostic stratification by molecular subtyping. Using the cut-off value derived from the TCGA-ESCA training set (0.6686536), patients were stratified into high- and low-risk groups, and Kaplan–Meier survival analysis showed that the low-risk group had significantly better OS than the high-risk group (Log-rank *p* = 0.01; Figure 3E). In the GSE53625 validation set, patients were similarly classified into high- and low-risk groups based on the cut-off value (1.902186), and the survival results were consistent with those in the training set: the low-risk group had significantly better OS than the high-risk group (Log-rank *p* = 0.019; Figure 3F), indicating that the model has robust prognostic predictive ability in an independent validation cohort.

**Figure 4:**
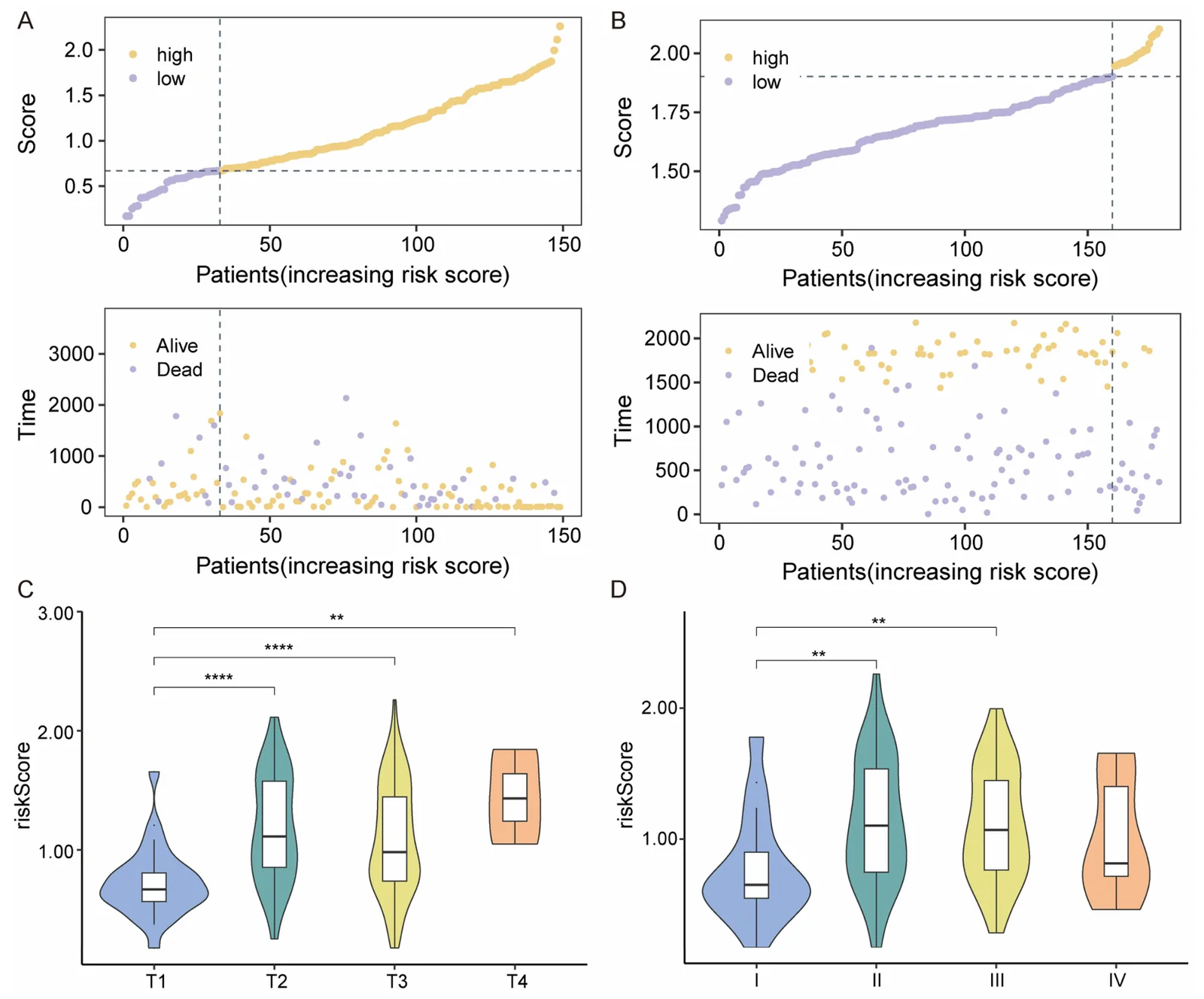
Validation of the MEMS risk model and correlation analysis with clinical characteristics. Note: (**A**) Distribution of risk scores (**top**) and scatter plot of survival status (**bottom**) in the TCGA cohort. Patients were ordered from low to high risk score; yellow dots indicate death, and purple dots indicate survival; the dashed line indicates the cut-off value. (**B**) Distribution of risk scores (**top**) and scatter plot of survival status (**bottom**) in the GSE53625 validation cohort, arranged as in (**A**). (**C**) Violin plot comparing risk scores among patients with different T stages; between-group differences were assessed using the Wilcoxon rank-sum test. (**D**) Violin plot comparing risk scores among patients with different clinical stages (Stage); between-group differences were assessed using the Wilcoxon rank-sum test. TCGA: The Cancer Genome Atlas; ESCA: Esophageal Cancer; MEMS: Mitochondrial Energy Metabolism Score. ** *p* < 0.01, **** *p* < 0.0001.

To further evaluate the model’s discriminative performance, we plotted risk score distributions and survival status maps for the TCGA cohort (Figure 4A) and the GSE53625 cohort (Figure 4B), respectively. The results indicated that, in both cohorts, patients in the high-risk group were more likely to be in the deceased status, suggesting that the model achieved robust prognostic discrimination in both the training and validation sets. Furthermore, we analyzed the relationship between risk scores and clinical stage, and found significant differences across T stages, with risk scores in T1 being markedly lower than those in T2, T3, and T4 (*p* < 0.01, Figure 4C). Similarly, risk scores differed significantly across Stage categories, with Stage I showing significantly lower scores than Stage II and Stage III (*p* < 0.01, Figure 4D), indicating that the risk score may effectively reflect tumor burden and the extent of clinical progression.

### 3.4. Differential Expression Analysis Between the High- and Low-Risk MEMS Risk-Model Subgroups in the TCGA-ESCA Dataset

**Figure 5:**
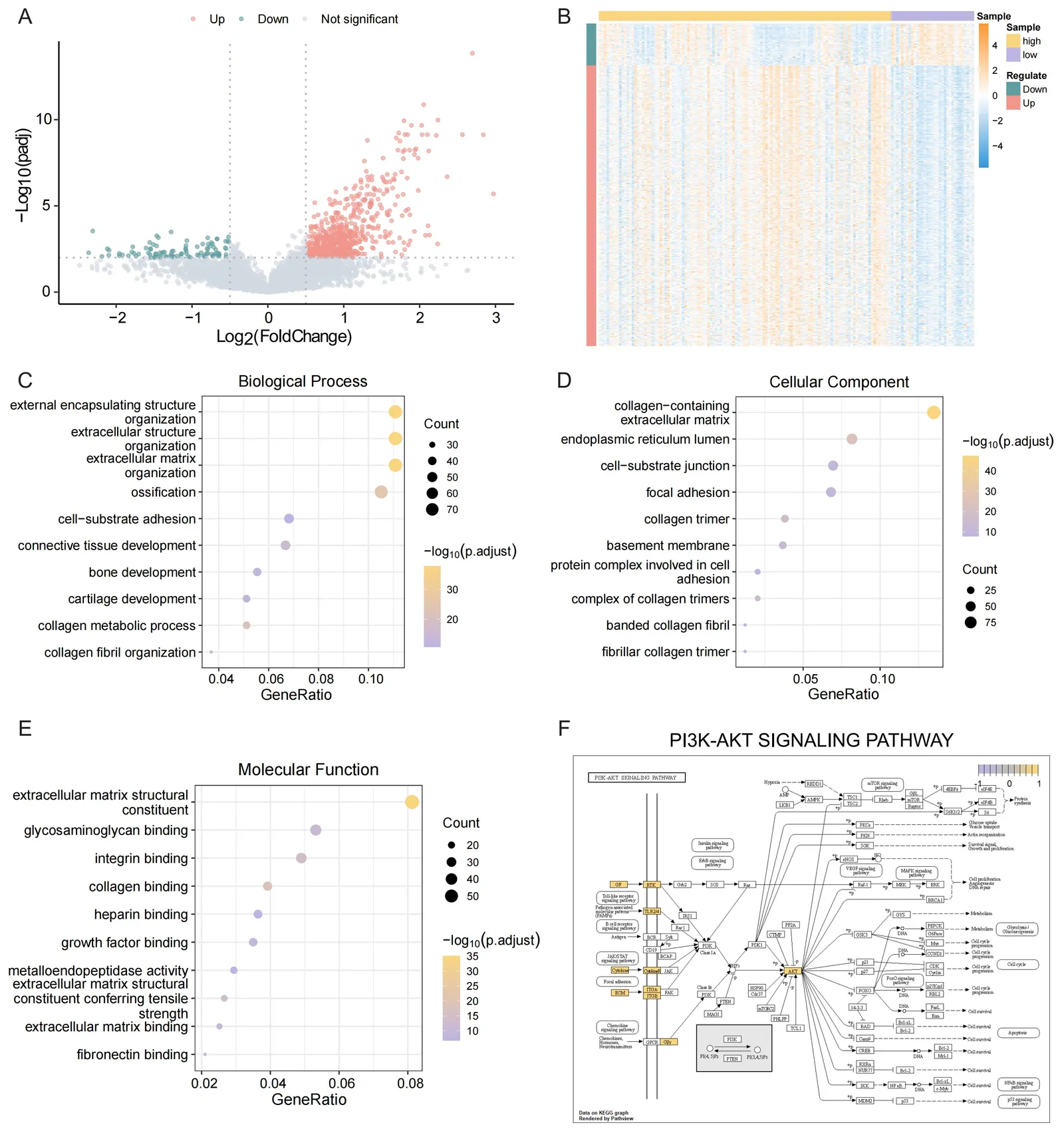
Differential expression and functional enrichment analyses between high- and low-risk groups in the TCGA-ESCA dataset. Note: (**A**) Volcano plot of DEGs between the high- and low-risk groups. The *x*-axis indicates log_2_FC and the *y*-axis indicates −log_10_(adjusted *p*-value). Red dots represent significantly upregulated genes, blue dots represent significantly downregulated genes, and gray dots represent genes with no significant difference. Dashed lines indicate the screening thresholds (|logFC| > 0.5 and adjusted *p*-value < 0.01). (**B**) Heatmap of DEGs. Rows represent genes and columns represent samples; the annotation bar at the top indicates high-/low-risk grouping. Colors from blue to red indicate relative gene expression from low to high. (**C**) Bubble plot of GO BP enrichment. Bubble size indicates the number of enriched genes (Count), and color intensity indicates −log_10_(adjusted *p*-value). (**D**) Bubble plot of GO CC enrichment, labeled as in (**C**). (**E**) Bubble plot of GO MF enrichment, labeled as in (**C**). (**F**) KEGG pathway map of the PI3K-Akt signaling pathway; red marks indicate mapped positions of DEGs. TCGA: The Cancer Genome Atlas; ESCA: Esophageal Cancer; BP: Biological Process; CC: Cellular Component; MF: Molecular Function; GO: Gene Ontology; KEGG: Kyoto Encyclopedia of Genes and Genomes.

To investigate the impact of the MEMS risk model on the initiation and progression of ESCA, patients in the TCGA-ESCA dataset were stratified into high- and low-risk groups using the risk score cut-off (0.6686536), followed by differential expression analysis. In total, 752 significantly DEGs were identified, including 654 upregulated and 98 downregulated genes (Figure 5A, Supplementary Material S7). Consistently, the heatmap of DEGs showed that most genes were expressed at higher levels in the high-risk group, whereas a smaller subset showed lower expression (Figure 5B).

To further explore the biological functions of the DEGs, we conducted GO functional annotation and KEGG pathway enrichment analyses for the 752 genes. The GO analysis results (Table 2) indicated that, within BP, the DEGs were primarily enriched in terms such as extracellular matrix organization, extracellular structure organization, external encapsulating structure organization, ossification, and collagen metabolic process (Figure 5C); within CC, they were mainly enriched in collagen-containing extracellular matrix, endoplasmic reticulum lumen, collagen trimer, complex of collagen trimers, basement membrane, and cell–substrate junction (Figure 5D); within MF, they were predominantly enriched in extracellular matrix structural constituent, collagen binding, integrin binding, extracellular matrix structural constituent conferring tensile strength, glycosaminoglycan binding, and fibronectin binding (Figure 5E). The KEGG enrichment results (Table 3) suggested that these genes were mainly enriched in pathways including protein digestion and absorption, proteoglycans in cancer, ECM-receptor interaction, focal adhesion, and the AGE-RAGE signaling pathway in diabetic complications. The pathway map for the PI3K-Akt signaling pathway is shown in Figure 5F. Collectively, these findings suggest that the DEGs between the high- and low-risk groups were largely involved in extracellular matrix remodeling, cell adhesion, and PI3K-Akt signaling pathways, and may play an important role in the malignant progression of ESCA.

### 3.5. GSEA Enrichment Analysis Based on the TCGA-ESCA Dataset

To further delineate BP differences between the high- and low-risk groups, we conducted GSEA using gene expression profiles from the TCGA-ESCA cohort (Table 4). The results indicated that inflammatory response (Figure S3A, normalized enrichment score [NES] = 2.2, *p* < 0.001, false discovery rate [FDR] < 0.001), apical junction (Figure S3B, NES = 1.969, *p* < 0.001, FDR < 0.001), hypoxia (Figure S3C, NES = 1.959, *p* < 0.001, FDR < 0.001), angiogenesis (Figure S3D, NES = 1.954, *p* < 0.001, FDR < 0.001), and myogenesis (Figure S3E, NES = 1.931, *p* < 0.001, FDR < 0.001) were significantly enriched in high-risk ESCA patients (NES > 0). In contrast, oxidative phosphorylation (Figure S3F, NES = −1.84, *p* < 0.001, FDR < 0.001), xenobiotic metabolism (Figure S3G, NES = −1.743, *p* < 0.001, FDR < 0.001), peroxisome (Figure S3H, NES = −1.528, *p* = 0.004, FDR = 0.009), and bile acid metabolism (Figure S3I, NES = −1.518, *p* = 0.003, FDR = 0.006) were significantly enriched in low-risk patients (NES < 0). Therefore, these findings suggest that pathways associated with malignant tumor progression, including the inflammatory response, hypoxia, and angiogenesis, were more active in the high-risk group, whereas metabolic pathways, such as oxidative phosphorylation, were relatively more active in the low-risk group.

### 3.6. Construction of the PPI Network and Related Regulatory Networks

**Figure 6:**
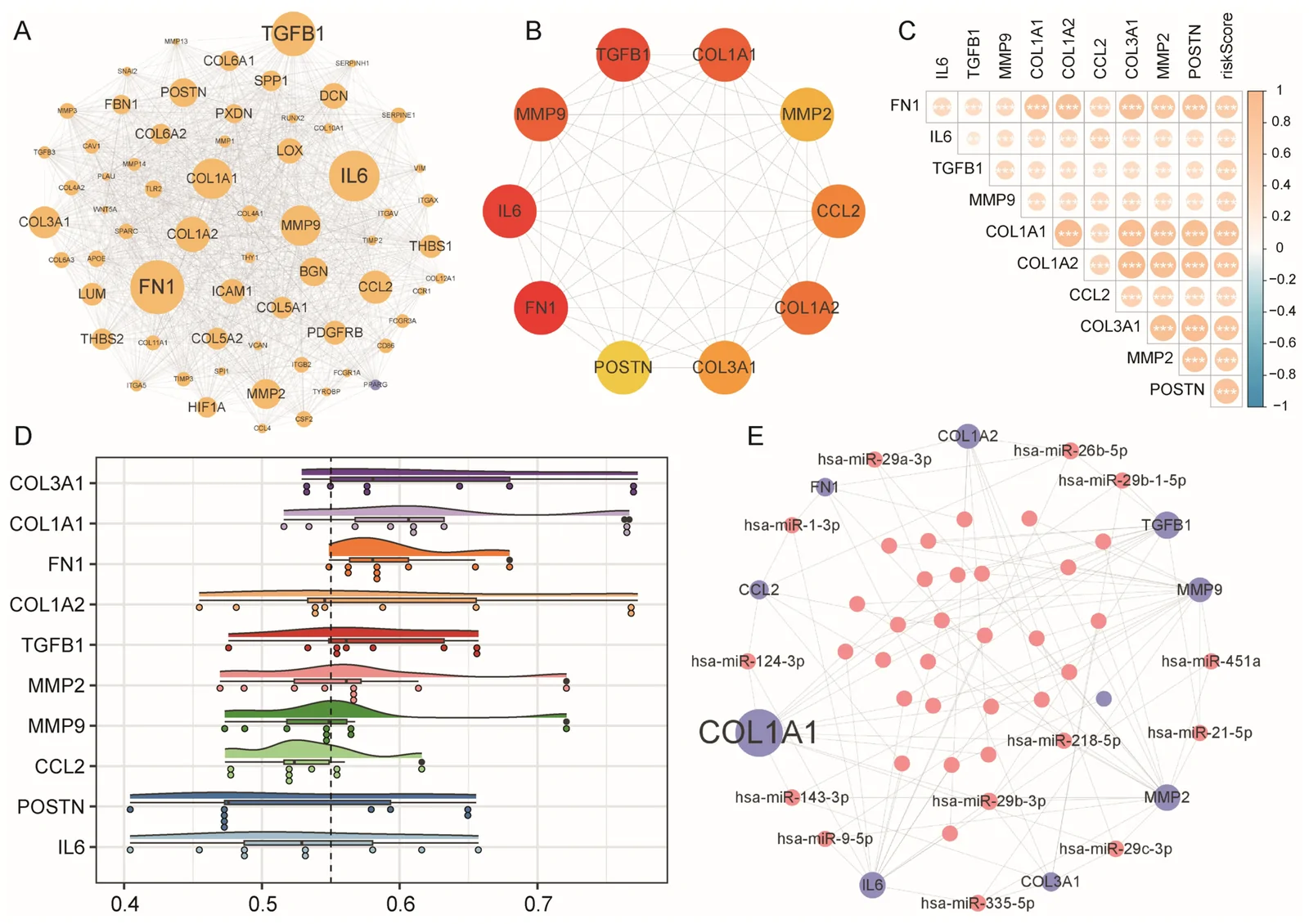
Construction of the PPI network and associated regulatory networks. Note: (**A**) PPI network constructed from DEGs; node size is proportional to connectivity (Degree), the color gradient from light yellow to dark orange indicates increasing connectivity from low to high, and edges represent PPIs. (**B**) Network of the top 10 hub genes ranked by the Degree algorithm using the CytoHubba plug-in; node color from yellow to red indicates increasing connectivity from low to high. (**C**) Pearson correlation heatmap between hub genes and the risk score; circle size represents the absolute value of the correlation coefficient, red indicates positive correlation, and blue indicates negative correlation. (**D**) Functional semantic similarity analysis (Friends analysis) of hub genes; the *x*-axis shows the functional similarity score, and different colors represent different genes. (**E**) miRNA–mRNA regulatory network associated with hub genes; diamond-shaped nodes indicate mRNAs, circular nodes indicate miRNAs, node size represents network connectivity, and edges indicate experimentally validated miRNA–mRNA targeting relationships. PPI: Protein–protein Interaction; TCGA: The Cancer Genome Atlas; ESCA: Esophageal Cancer. ** *p* < 0.01, *** *p* < 0.001.

To characterize interaction patterns among DEGs between the high- and low-risk groups, we performed a protein interaction analysis for 752 DEGs using the STRING database and constructed a PPI network. The resulting network contained 639 nodes and 6006 edges, whereas 113 isolated nodes were excluded from subsequent analyses. We visualized the PPI network using Cytoscape (Figure 6A), where node size was proportional to connectivity, and genes such as IL6, FN1, and MMP9 exhibited relatively high degrees. We then used the CytoHubba plugin to rank nodes by degree and identified the top 10 hub genes: FN1, IL6, TGFB1, MMP9, COL1A1, COL1A2, CCL2, COL3A1, MMP2, and POSTN (Figure 6B). Notably, darker node colors indicated higher connectivity, and FN1, IL6, and TGFB1 showed the darkest colors, suggesting their central regulatory roles within the interaction network.

To further evaluate the importance of the hub genes, we conducted Pearson correlation analyses between hub gene expression levels and the risk score. The results showed a significant co-expression pattern among the hub genes, and all hub genes were positively correlated with the risk score (Figure 6C); for example, FN1 was strongly positively correlated with COL1A2 (cor = 0.855, *p* < 0.001), and COL1A1 was strongly positively correlated with the risk score (cor = 0.847, *p* < 0.001). We subsequently performed a functional semantic similarity analysis of the hub genes (Friends analysis), which indicated that COL3A1 had the highest functional similarity score among the hub genes, suggesting its important role in the functional network of hub genes (Figure 6D).

Finally, to delineate post-transcriptional regulatory relationships, we constructed an miRNA–mRNA regulatory network (Figure 6E, Supplementary Material S8) using experimentally validated miRNA–mRNA interactions from the miRTarBase database, with the hub genes designated as targets. This network comprised 240 miRNAs and 10 mRNAs, in which mRNAs such as COL1A1, COL1A2, and MMP9, as well as miRNAs such as hsa-miR-29b-3p, hsa-miR-26b-5p, hsa-miR-143-3p, and hsa-miR-29c-3p, exhibited relatively high connectivity. Therefore, these molecules may play key roles in post-transcriptional regulation in ESCA.

### 3.7. Immune Cell Infiltration Analysis Based on the TCGA-ESCA Dataset

To evaluate the impact of the MEMS risk score on the overall immune landscape and the infiltration levels of different immune cells in ESCA patients, the stromal and ESTIMATE scores for each patient were calculated using the ESTIMATE algorithm. The results showed that the risk score exhibited a significant positive correlation with the stromal score (cor = 0.55, *p* = 3.3 × 10^−13^, Figure S4A) and the ESTIMATE score (cor = 0.37, *p* = 2.8 × 10^−6^, Figure S4B), indicating that patients in the high-risk group had a higher abundance of stromal components in the TME and relatively lower tumor purity.

Subsequently, the ssGSEA algorithm was used to quantify the infiltration abundance of each immune cell subtype, and the differences between the high- and low-risk groups were compared. The results showed that more than 70% of the immune cell subtypes differed significantly between the high- and low-risk groups (*p* < 0.05), and the infiltration levels of most immune cells were significantly higher in the high-risk group than in the low-risk group (Figure S4C).

To further analyze the relationship between hub genes and immune infiltration, correlation analyses were performed between hub gene expression levels and infiltration scores for various immune cell subtypes. The results showed that most hub genes were significantly positively correlated with immune cell infiltration, including natural killer T cells and regulatory T cells. For example, MMP9 was significantly positively correlated with regulatory T cell infiltration (cor = 0.715, *p* < 2.2 × 10^−16^), and CCL2 was significantly positively correlated with natural killer T cell infiltration (cor = 0.618, *p* < 2.2 × 10^−16^). In contrast, most hub genes were negatively correlated with the infiltration of immune cells, including CD56^bright^ natural killer cells and neutrophils. For instance, TGFB1 showed a significant negative correlation with CD56^dim^ natural killer cell infiltration (cor = −0.439, *p* < 2.2 × 10^−16^) (Figure S4D).

In addition, the expression correlations between hub genes and immune-related genes were analyzed. The results indicated that genes such as TGFB1 and COL1A1 were negatively correlated with most immune-related genes; for example, TGFB1 was significantly negatively correlated with BTNL9 (cor = −0.384, *p* = 1.335 × 10^−6^), and COL1A1 was significantly negatively correlated with CEACAM1 (cor = −0.467, *p* = 1.845 × 10^−9^). Conversely, genes such as CCL2 and MMP9 were positively correlated with most immune-related genes; for instance, CCL2 was significantly positively correlated with LAG3 (cor = 0.437, *p* = 2.525 × 10^−8^), and MMP9 was significantly positively correlated with HLA-DMA (cor = 0.201, *p* = 0.0003) (Figure S4E). Furthermore, the expression differences of immune-related genes between the high- and low-risk groups were compared. The results showed that all immune-related genes differed significantly between the two groups (*p* < 0.05), and the expression of most immune-related genes was significantly higher in the low-risk group than in the high-risk group, such as the significantly high expression of BTNL9 and BTNL3 in the low-risk group (*p* < 0.05, Figure S4F).

### 3.8. Impact of the MEMS Risk Score on Genomic Alterations in Patients with TCGA-ESCA

To characterize the impact of the MEMS risk score on the landscape of genetic alterations in patients with TCGA-ESCA, we compared somatic mutations and CNV profiles between the high- and low-risk groups. The somatic mutation analysis indicated that the overall mutation frequency was higher in the high-risk group than in the low-risk group (high-risk: 99.13%, Figure S5A; low-risk: 93.94%, Figure S5B). In both groups, TP53 was the most frequently mutated gene, with mutation rates of 83% in the high-risk group and 79% in the low-risk group; however, TTN showed a slightly higher mutation rate in the low-risk group (42%) than in the high-risk group (41%). The CNV analysis further suggested that, compared with the low-risk group, patients in the high-risk group exhibited a markedly increased burden of copy-number alterations, with deletions being particularly prominent (high-risk: Figure S5C; low-risk: Figure S5D).

To further estimate potential responses to immune checkpoint inhibitors in patients with TCGA-ESCA, we applied the TIDE algorithm (Supplementary Material S9). The results showed that the TIDE score was significantly higher in the high-risk group than in the low-risk group (*p* < 0.001, Figure S5E), suggesting that patients in the high-risk group might respond less favorably to immunotherapy. In addition, ROC analysis indicated that the risk score had good predictive performance for immunotherapy sensitivity in ESCA (AUC = 0.804, Figure S5F). Therefore, the distribution of predicted response status further demonstrated that the proportion of predicted non-responders was substantially higher in the high-risk group (69%) than in the low-risk group (21.2%) (Figure S5G), supporting a lower likelihood of immunotherapy benefit in the high-risk group.

### 3.9. Construction of a Clinical Prognostic Model Based on the Risk Score in the TCGA-ESCA Dataset

To determine whether the risk score and clinicopathological characteristics were independent prognostic factors, we conducted univariate and multivariate Cox regression analyses using complete cases for the variables included in each model. The univariate Cox analysis showed that the high-risk group (HR = 2.22, 95% CI 1.07–4.60, *p* = 0.032), N1 versus N0 (HR = 2.22, 95% CI 1.09–4.51, *p* = 0.028), M1 versus M0 (HR = 3.28, 95% CI 1.42–7.57, *p* = 0.005), Stage III versus Stage I (HR = 5.58, 95% CI 1.45–21.50, *p* = 0.012), and Stage IV versus Stage I (HR = 9.04, 95% CI 1.99–41.04, *p* = 0.004) were significantly associated with OS (Figure S6A, Table 5). The multivariable model included the risk group and M stage and was fitted in the 127 patients with complete data for both variables (42 deaths). The multivariate Cox analysis further indicated that the high-risk group was not independently associated with OS (HR = 1.81, 95% CI 0.85–3.83, *p* = 0.122), whereas M1 versus M0 remained significantly associated with OS (HR = 2.87, 95% CI 1.23–6.69, *p* = 0.015) (Figure S6B, Table 6).

Based on the foregoing results, we integrated the risk score with M stage to develop a predictive nomogram for individualized assessment of OS status in ESCA patients (Figure S6C). This nomogram enables the calculation of a total score from each patient’s risk score and M stage, thereby predicting 1-, 2-, and 3-year survival probabilities. Calibration curve analyses indicated good agreement between the nomogram-predicted and actual observed OS at 1 year (Figure S6D), 2 years (Figure S6E), and 3 years (Figure S6F),whereas bootstrap internal validation yielded an apparent Harrell C-index of 0.613 and an optimism-corrected C-index of 0.605 (95% empirical interval, 0.514–0.690), with a mean calibration slope of 0.922 (95% empirical interval, 0.477–1.837), indicating limited discrimination and appreciable uncertainty.

### 3.10. Expression Characteristics of INHBA in Esophageal Squamous Cell Carcinoma Cells and Construction of an Intervention Model

In the prognostic model established in this study, four key genes (COL11A1, INHBA, TNFAIP6, and POSTN) were identified. To define the optimal target for subsequent in vitro functional experiments, we performed an integrated prioritization of these four key genes.

First, INHBA was significantly associated with prognosis in the univariate Cox regression analysis and had the highest regression coefficient among the four genes (coefficient = 0.19), indicating the strongest contribution to the risk score model. Second, INHBA expression was significantly and positively correlated with the risk score and was markedly upregulated in the high-risk group, suggesting that it may play a critical role in the development of high-risk phenotypes. Furthermore, combined with functional enrichment analysis, the DEGs in this study were mainly enriched in pathways such as extracellular matrix organization, focal adhesion, and the PI3K-Akt signaling pathway, whereas prior studies have demonstrated that INHBA/Activin A can participate in tumor cell migration, invasion, and EMT by activating Smad2/3-related signaling, thereby exerting pro-tumorigenic effects across multiple cancers [18,25]. In addition, previous studies in esophageal squamous cell carcinoma have confirmed that high INHBA expression is significantly associated with poor prognosis, and that Activin A/INHBA-related signaling can promote invasive phenotypes of esophageal squamous cell carcinoma cells; together with its canonical downstream Smad2/3 signaling mechanism, these findings suggest that INHBA may be involved in esophageal squamous cell carcinoma progression via the Activin A–Smad axis [17,25,26]. Therefore, integrating our bioinformatics analysis results with existing literature evidence, we selected INHBA as the representative key gene for subsequent in vitro functional and mechanistic validation experiments.

To screen a suitable cell model for subsequent functional experiments, we first quantified INHBA expression across different esophageal squamous cell carcinoma cell lines using qRT-PCR and WB. The results indicated that there were significant differences in INHBA expression among KYSE-150, KYSE-450, and TE-1 cells, with KYSE-150 cells showing the highest mRNA and protein levels, whereas TE-1 cells showed the lowest (Figure S7A,B). Based on these results, we selected KYSE-150 cells with high INHBA expression for knockdown experiments and TE-1 cells with low INHBA expression for overexpression experiments.

To validate the intervention efficiency 48 h after transfection, qRT-PCR was conducted. The results indicated that, compared with the corresponding control groups, INHBA mRNA expression was significantly decreased in the INHBA knockdown group (si-INHBA), whereas it was significantly increased in the INHBA overexpression group (oe-INHBA) (Figure S7C,D). Therefore, WB further corroborated that the direction of change in INHBA protein expression was consistent with that at the mRNA level (Figure S7E,F). Collectively, these findings suggest that the INHBA knockdown and overexpression cell models were successfully established and could be used for subsequent functional experiments.

### 3.11. INHBA Promotes Malignant Phenotypes of Esophageal Squamous Cell Carcinoma Cells and Enhances Three-Dimensional Matrix Invasion Capacity

**Figure 7:**
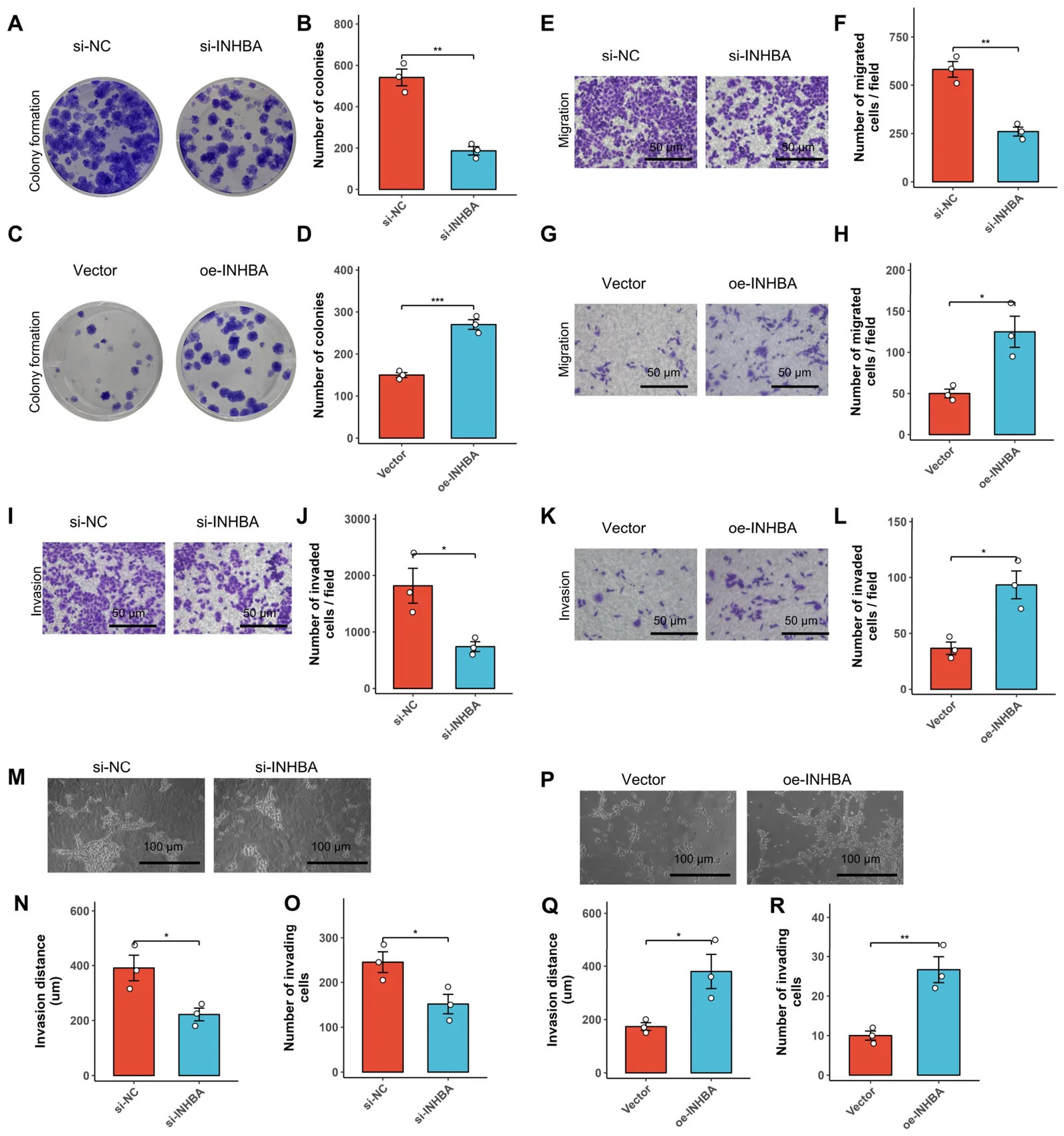
INHBA promotes malignant phenotypes of esophageal squamous cell carcinoma cells and enhances 3D matrix invasion. Note: (**A**,**B**) Representative images (**A**) and quantification of colony numbers (**B**) of colony formation assays in KYSE-150 cells after si-INHBA transfection. (**C**,**D**) Representative images (**C**) and quantification of colony numbers (**D**) of colony formation assays in TE-1 cells after oe-INHBA transfection. (**E**,**F**) Representative images (**E**) and quantification of migrated cells (**F**) of Transwell migration assays in KYSE-150 cells after si-INHBA transfection. (**G**,**H**) Representative images (**G**) and quantification of migrated cells (**H**) of Transwell migration assays in TE-1 cells after oe-INHBA transfection. (**I**,**J**) Representative images (**I**) and quantification of invaded cells (**J**) of Transwell invasion assays in KYSE-150 cells after si-INHBA transfection. (**K**,**L**) Representative images (**K**) and quantification of invaded cells (**L**) of Transwell invasion assays in TE-1 cells after oe-INHBA transfection. (**M**–**O**) Representative images (**M**), quantification of invasion distance (**N**), and quantification of invading cell numbers (**O**) of 3D collagen invasion assays in KYSE-150 cells after si-INHBA transfection. (**P**–**R**) Representative images (**P**), quantification of invasion distance (**Q**), and quantification of invading cell numbers (**R**) of 3D collagen invasion assays in TE-1 cells after oe-INHBA transfection. Colonies with ≥50 cells were counted in the colony formation assay; in Transwell assays, cells that migrated through the membrane were counted in five randomly selected fields (scale bar: 50 μm). The 3D collagen invasion assay used type I collagen to construct a 3D matrix model (scale bar: 100 μm). Data are presented as mean ± SD; experiments were independently repeated three times. * *p* < 0.05, ** *p* < 0.01, *** *p* < 0.001.

To elucidate the effects of INHBA on malignant biological behaviors in esophageal squamous cell carcinoma cells, CCK-8 assays were first conducted to evaluate short-term proliferative capacity. The results indicated that, compared with the corresponding control groups, INHBA knockdown markedly reduced the proliferative activity of KYSE-150 cells at 48 h, 72 h, and 96 h, whereas INHBA overexpression significantly increased the proliferation rate of TE-1 cells (Figure S8A,B). Moreover, colony formation assays showed that INHBA knockdown substantially reduced the colony-forming ability of KYSE-150 cells, whereas INHBA overexpression significantly increased the number of colonies formed by TE-1 cells (Figure 7A–D).

To further characterize the effects of INHBA on motility-related phenotypes, wound-healing and Transwell assays were performed to assess cell migration and invasion. The wound-healing assay indicated that INHBA knockdown markedly decreased the migratory capacity of KYSE-150 cells; in contrast, INHBA overexpression significantly promoted TE-1 cell migration (Figure S9A,B). Consistently, Transwell assays further suggested that the numbers of migrating and invading cells were significantly reduced after INHBA knockdown, whereas the numbers of cells traversing the membrane were markedly increased after INHBA overexpression (Figure 7E–L).

To more closely mimic invasive behavior within an extracellular matrix environment, a three-dimensional collagen invasion assay was conducted for validation. The results indicated that INHBA knockdown markedly decreased both the invasion distance into the collagen matrix and the number of invading cells, whereas INHBA overexpression significantly enhanced invasion ability within the three-dimensional matrix (Figure 7M–R). Taken together, these findings suggest that INHBA not only promotes proliferation, migration, and invasion of esophageal squamous cell carcinoma cells under two-dimensional culture conditions but also enhances their invasive phenotype in a three-dimensional matrix environment, thereby indicating a pro-tumorigenic role of INHBA during the malignant progression of esophageal squamous cell carcinoma.

### 3.12. INHBA Promotes EMT and Activates the Smad2/3 Signaling Pathway

**Figure 8:**
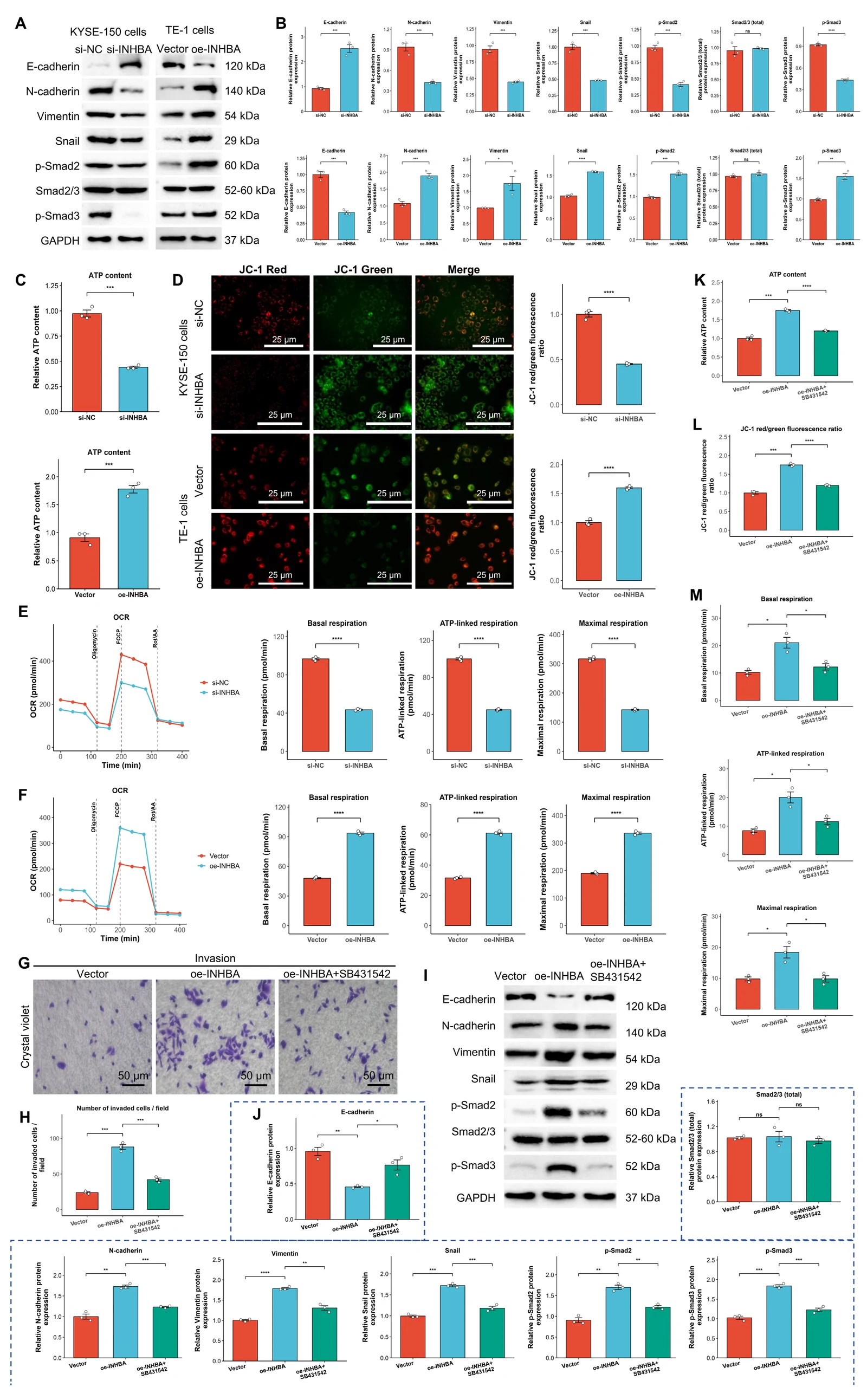
INHBA regulates EMT, mitochondrial metabolic reprogramming, and Smad2/3-related malignant phenotypes in esophageal squamous cell carcinoma cells. Note: (**A**) Representative WB images of EMT-related proteins and Smad2/3 pathway-related proteins after INHBA knockdown and overexpression; (**B**) quantification of band intensities for proteins in (**A**), normalized to GAPDH; (**C**) cellular ATP levels after INHBA knockdown and overexpression; (**D**) representative JC-1 staining images and red/green fluorescence ratio; (**E**,**F**) Seahorse OCR curves and mitochondrial respiration parameters in KYSE-150 cells after si-INHBA transfection (**E**) and TE-1 cells after oe-INHBA transfection (**F**); (**G**,**H**) representative Transwell invasion images and invaded-cell quantification in INHBA-overexpressing TE-1 cells treated with SB431542; (**I**,**J**) representative WB images and quantification of EMT- and Smad2/3-related proteins after SB431542 treatment; and (**K**–**M**) ATP content, JC-1 red/green fluorescence ratio, and Seahorse OCR parameters after SB431542 treatment. Data are presented as mean ± SD from n = 3 independent experiments. * *p* < 0.05, ** *p* < 0.01, *** *p* < 0.001, **** *p* < 0.0001; ns, not significant. The original Western blot images are all included in the Supplementary Materials S10.

To elucidate the molecular mechanism by which INHBA promotes the malignant progression of esophageal squamous cell carcinoma cells, WB was performed to quantify changes in EMT-related and Smad2/3 signaling pathway proteins. The results indicated that, relative to the corresponding control groups, INHBA knockdown increased the expression of the epithelial marker e-cadherin, whereas it decreased the expression of the mesenchymal markers n-cadherin and vimentin as well as the transcription factor snail; in contrast, INHBA overexpression decreased e-cadherin expression while increasing n-cadherin, vimentin, and snail expression (Figure 8A,B). Furthermore, INHBA knockdown markedly reduced the phosphorylation levels of p-Smad2 and p-Smad3, whereas total Smad2/3 protein levels showed no evident change; conversely, INHBA overexpression significantly increased the phosphorylation levels of p-Smad2 and p-Smad3 (Figure 8A,B). Collectively, these findings suggest that INHBA may promote the EMT process in esophageal squamous cell carcinoma cells by activating the Smad2/3 signaling pathway.

### 3.13. INHBA Maintains Mitochondrial Function and Promotes Oxidative Phosphorylation Metabolism

To further clarify the association between INHBA and mitochondrial energy metabolism, we measured ATP content, mitochondrial membrane potential, and mitochondrial respiratory function across groups. The results showed that INHBA knockdown significantly decreased cellular ATP levels, whereas INHBA overexpression markedly increased ATP levels (Figure 8C). JC-1 assays indicated that INHBA knockdown reduced the red/green fluorescence ratio, suggesting a decrease in mitochondrial membrane potential; however, INHBA overexpression significantly increased the red/green fluorescence ratio, indicating an increase in mitochondrial membrane potential (Figure 8D). Seahorse metabolic analysis further demonstrated that INHBA knockdown reduced basal respiration, ATP-linked respiration, and maximal respiratory capacity, whereas INHBA overexpression significantly increased these metrics (Figure 8E,F). In addition, ROS assays showed that intracellular ROS levels increased after INHBA knockdown but decreased after INHBA overexpression (Figure S10A,B), indicating that INHBA modulation may alter intracellular oxidative stress status. Therefore, these results indicate that INHBA helps maintain mitochondrial function and oxidative phosphorylation metabolic activity in esophageal squamous cell carcinoma cells.

### 3.14. Blockade of the Smad Pathway Partially Reverses INHBA-Mediated EMT and Mitochondrial Metabolic Reprogramming

To determine whether the functional effects of INHBA depend on the Smad2/3 signaling pathway, we treated INHBA-overexpressing TE-1 cells with the ALK5 inhibitor SB431542 (10 μM). The Transwell invasion assay showed that SB431542 markedly reduced the number of invading cells compared with the INHBA overexpression group (Figure 8G,H). WB analysis indicated that SB431542 substantially suppressed the INHBA overexpression-induced increases in p-Smad2 and p-Smad3, restored E-cadherin expression, and decreased N-cadherin, Vimentin, and Snail expression (Figure 8I,J). Moreover, SB431542 partially reversed the INHBA overexpression-associated increases in ATP levels (Figure 8K), mitochondrial membrane potential (Figure 8L), and OCR (Figure 8M). Therefore, these findings suggest that INHBA promotes EMT and mitochondrial metabolic reprogramming in esophageal squamous cell carcinoma cells, at least in part through the Smad2/3 signaling pathway.

**Figure 9:**
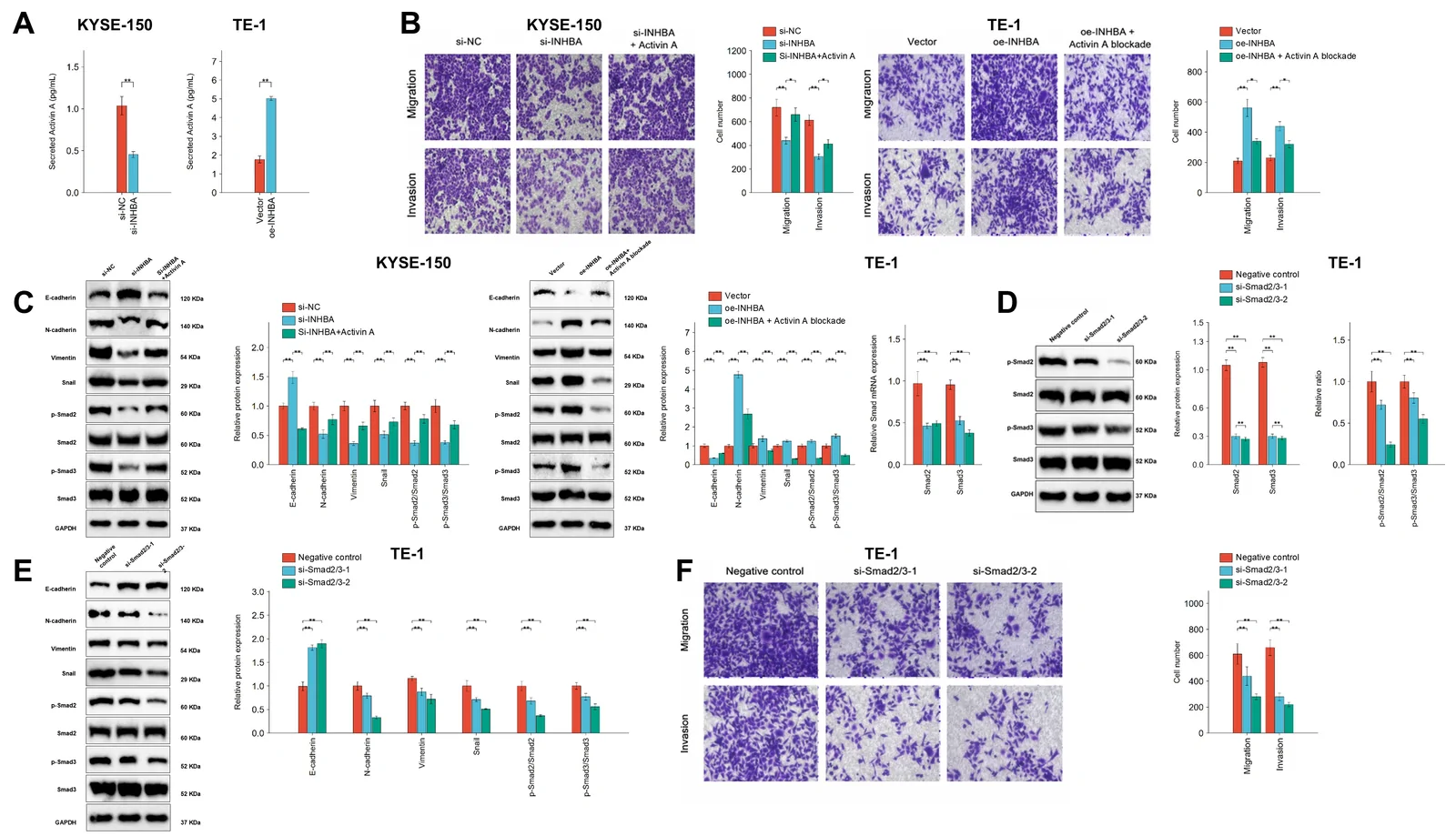
Activin A-axis rescue/blockade and genetic Smad2/3 inhibition support INHBA-associated malignant phenotypes in esophageal squamous cell carcinoma cells. Note: (**A**) ELISA analysis of secreted Activin A levels after INHBA knockdown in KYSE-150 cells and INHBA overexpression in TE-1 cells; (**B**) representative Transwell migration/invasion images and quantification after Activin A rescue in KYSE-150 cells and Activin A blockade in TE-1 cells; (**C**) representative WB images and quantification of EMT-related proteins and p-Smad2/Smad2 and p-Smad3/Smad3 after Activin A-axis modulation; (**D**) qRT-PCR and WB validation of Smad2/3 knockdown efficiency and Smad2/3 pathway activity in oe-INHBA TE-1 cells transfected with si-Smad2/3-1 or si-Smad2/3-2; (**E**) representative WB images and quantification of EMT-related proteins and Smad2/3 phosphorylation after Smad2/3 knockdown; (**F**) representative Transwell migration/invasion images and quantification after Smad2/3 knockdown. Data are presented as mean ± SD from n = 3 independent experiments. * *p* < 0.05, ** *p* < 0.01. The original Western blot images are all included in the Supplementary Materials S10.

To strengthen the proposed INHBA/Activin A mechanism, we measured secreted Activin A in cell-culture supernatants and performed Activin A-axis rescue/blockade assays. INHBA knockdown reduced secreted Activin A in KYSE-150 cells, whereas INHBA overexpression increased secreted Activin A in TE-1 cells (Figure 9A). Functionally, recombinant Activin A partially restored the migration and invasion suppressed by si-INHBA in KYSE-150 cells, whereas Activin A blockade attenuated the migration and invasion enhanced by oe-INHBA in TE-1 cells (Figure 9B). Consistently, Activin A rescue partially reversed the si-INHBA-induced suppression of mesenchymal markers and Smad2/3 phosphorylation, whereas Activin A blockade partially reversed oe-INHBA-induced EMT activation and Smad2/3 phosphorylation (Figure 9C).

To address the specificity of Smad2/3 signaling, we next performed genetic Smad2/3 inhibition in oe-INHBA TE-1 cells. Both si-Smad2/3-1 and si-Smad2/3-2 reduced Smad2/3 mRNA and protein expression and decreased p-Smad2/Smad2 and p-Smad3/Smad3 ratios (Figure 9D). Smad2/3 inhibition increased E-cadherin while decreasing N-cadherin, Vimentin, Snail, p-Smad2/Smad2, and p-Smad3/Smad3 (Figure 9E), and it also reduced migration and invasion in the oe-INHBA background (Figure 9F). These results further support that Smad2/3-related signaling contributes to INHBA-mediated EMT and malignant phenotypes.

Because INHBA also affected cell proliferation, we repeated the migration assays under proliferation-controlled conditions. INHBA knockdown still reduced KYSE-150 wound closure and Transwell migration, whereas INHBA overexpression still enhanced TE-1 wound closure and Transwell migration (Figure S11A–D). Parallel cell-viability monitoring showed no significant short-term viability difference during the migration assay window (Figure S11E), supporting that the observed migration changes were not solely attributable to cell-growth differences.

Finally, we examined whether INHBA knockdown altered chemotherapy sensitivity. Compared with si-NC cells, si-INHBA-transfected cells showed left-shifted dose–response curves and lower viability after treatment with Cisplatin, Paclitaxel, 5-FU, and Docetaxel (Figure S12A,C). IC_50_ values were reduced from 5.23 to 1.21 μM for Cisplatin, from 18.6 to 5.12 nM for Paclitaxel, from 642.3 to 172.4 μM for 5-FU, and from 22.7 to 6.38 nM for Docetaxel (Figure S12B), indicating that INHBA knockdown increased chemotherapy sensitivity.

## 4. Discussion

This study evaluated the prognostic stratification value of MEMRGs in ESCA using the TCGA-ESCA cohort and the GSE53625 validation cohort. The results showed that patients in the high MEMS group had poorer OS; differential expression analysis combined with WGCNA identified 124 key genes, which were further used to construct MEMS-related molecular subtyping. A prior TCGA integrative analysis reported substantial molecular differences between esophageal squamous cell carcinoma and esophageal adenocarcinoma, indicating that prognostic research in ESCA should account for tissue origin and molecular heterogeneity [3]. Consistent with previous work, our findings support the notion that molecular features can complement conventional clinicopathological stratification; however, unlike approaches based solely on mutation spectra, histological types, or immune features, we used the MEMS as the entry point for classification. Because the TCGA-ESCA dataset comprises samples with distinct histological origins, the association between MEMS and prognosis may still be influenced by cohort composition and platform heterogeneity; therefore, the conclusion is more appropriately presented as an indicative observation.

This study identified three molecular subtypes via consensus clustering, among which Cluster 2 exhibited relatively poor prognosis, suggesting that MEMRGs may help explain intratumoral heterogeneity in ESCA. Previous evidence has linked mitochondrial energy metabolism to an immunosuppressive TME and unfavorable outcomes in esophageal squamous cell carcinoma [11], and our findings are directionally consistent with this report. However, unlike prior studies, we integrated ssGSEA, differential expression analysis, and WGCNA to select genes associated with both MEMS grouping and co-expression modules, and then used these genes for molecular subtyping. Although this strategy may improve alignment between candidate genes and the research theme, it may also overlook non-MEMRGs, non-coding RNAs, or epigenetic regulatory factors. Because this subtyping scheme has not yet been replicated in an independent cohort, its stability and clinical applicability require further evaluation.

Regarding the prognostic model, this study ultimately included four genes (COL11A1, INHBA, TNFAIP6, and POSTN) and consistently observed survival differences between the high- and low-risk groups in both the training and validation sets. A previous study constructed an 8-gene prognostic signature based on energy metabolism-related genes in ESCA and combined it with a nomogram to predict OS [10]. Similar to that work, we used Cox regression and LASSO regression to select prognosis-related genes and performed external validation in a GEO cohort. However, the genes in our model include multiple molecules related to the extracellular matrix, adhesion, and tumor stroma, suggesting that the risk score may not solely reflect the metabolic status within tumor cells but may also be associated with extracellular matrix remodeling and an invasive phenotype. Notably, differences across public databases in detection platforms, sample processing, and completeness of clinical annotations mean that, although external validation enhances credibility, batch effects and selection bias cannot be fully excluded.

Functional enrichment analyses indicated that DEGs between the high- and low-risk groups were mainly enriched in extracellular matrix remodeling, cell adhesion, ECM-receptor interaction, focal adhesion, and the PI3K-Akt signaling pathway; GSEA further suggested increased activity of inflammatory response, hypoxia, and angiogenesis pathways in the high-risk group, whereas oxidative phosphorylation was relatively active in the low-risk group. These findings are broadly consistent with prior understanding that tumor stroma, inflammation, and hypoxia contribute to the malignant progression of ESCA [27,28]. Notably, oxidative phosphorylation was relatively enriched in the low-risk group at the cohort level, whereas in vitro experiments showed that INHBA overexpression maintained mitochondrial membrane potential and oxidative phosphorylation activity; however, these observations are not necessarily contradictory. Specifically, the former derives from risk stratification based on bulk RNA-seq cohorts, whereas the latter reflects a post-single-gene perturbation cellular model, and therefore represents different biological levels. Therefore, our results support an association between the MEMS risk score and extracellular matrix remodeling, inflammatory/hypoxic states, and metabolic status, but do not justify inferring a direct causal relationship.

Immune infiltration and TIDE analyses suggest that the high-risk group may exhibit greater stromal abundance and a poorer response to immunotherapy. Consistent with this observation, prior single-cell studies reported immunosuppression-associated states in the TME of esophageal squamous cell carcinoma, including exhausted T/NK cells, regulatory T cells, alternatively activated macrophages, and tolerogenic dendritic cells [29]. Therefore, TIDE has been applied to predict the potential response to immune checkpoint inhibitors from the perspectives of T-cell dysfunction and exclusion [8]. In this study, TIDE was used to estimate immunotherapy responsiveness in the high- and low-risk groups, which aligns with the prevailing transcriptome-based strategy for inferring immune sensitivity. However, because no real-world immunotherapy cohort was included, the TIDE results only indicate a potential trend and cannot substitute for clinical efficacy validation. In addition, immune infiltration estimated from bulk RNA-seq cannot reliably distinguish whether signals originate from tumor cells, immune cells, or stromal cells; therefore, single-cell or spatial transcriptomics will be required to address this limitation in future work.

To further support the bioinformatic findings, this study selected INHBA for in vitro validation; results indicated that INHBA knockdown suppressed proliferation, migration, invasion, and three-dimensional matrix invasion capacity in esophageal squamous cell carcinoma cells, whereas overexpression showed the opposite trend. In line with previous evidence that INHBA is highly expressed in esophageal squamous cell carcinoma and is associated with poor prognosis [17], the present findings are largely consistent with earlier reports. In contrast, this study prioritized INHBA within the MEMS risk model and further examined its associations with EMT, the Smad2/3 pathway, and mitochondrial functional readouts. SB431542 partially reversed the phenotypes associated with INHBA overexpression, suggesting that the Smad2/3 pathway may contribute; however, the “partial reversal” also implies that additional factors are likely involved. The limitations of this study include a limited sample size in public datasets and platform heterogeneity, the lack of independent validation for molecular subtyping, the reliance on in vitro experiments primarily using cell lines that may not fully represent patient tumors, and the fact that TIDE reflects computational prediction rather than actual treatment response. Therefore, large multicenter cohort validation, clinical tissue assessment, single-cell localization, and animal model studies are still warranted to evaluate further the robustness and translational value of the model and the INHBA/Smad2/3 axis.

The additional Activin A rescue/blockade and Smad2/3 siRNA experiments further refine the mechanistic interpretation of this study. These data support a model in which INHBA promotes ESCC cell motility and EMT at least partly through an Activin A-related Smad2/3 signaling context, rather than solely through the broad pharmacological effects of SB431542. Moreover, the proliferation-controlled migration assays indicate that INHBA-associated motility changes are not simply a secondary consequence of short-term growth differences. The chemotherapy-sensitivity assays further suggest that INHBA knockdown increases the response of ESCC cells to Cisplatin, Paclitaxel, 5-FU, and Docetaxel, providing additional functional relevance for the INHBA-associated EMT phenotype.

## 5. Conclusions

Through systematic bioinformatic analyses, this study established a prognostic risk score model and a molecular subtyping framework for ESCA based on MEMRGs. The results revealed that patients with higher MEMS exhibited significantly poorer prognosis. A four-gene risk score model comprising COL11A1, INHBA, TNFAIP6, and POSTN demonstrated robust prognostic stratification in both the TCGA training and GEO validation cohorts. Moreover, the risk score was significantly associated with tumor T stage and clinical stage, and a nomogram integrating risk group and M stage showed limited optimism-corrected discrimination for 1- to 3-year survival. Functional enrichment and immune infiltration analyses further revealed that the high-risk group was characterized by enhanced activities in pathways such as extracellular matrix remodeling, inflammatory responses, and hypoxia, accompanied by increased stromal abundance in the TME and reduced immunotherapy responsiveness. This provides a reference for selecting immunotherapy strategies for ESCA. More importantly, in vitro functional assays and mechanistic studies confirmed the tumor-promoting role of the key gene INHBA in the malignant progression of esophageal squamous cell carcinoma. INHBA promotes the EMT process by activating the Smad2/3 signaling pathway while maintaining mitochondrial membrane potential and oxidative phosphorylation, thereby driving mitochondrial metabolic reprogramming. Furthermore, the ALK5 inhibitor SB431542 partially reversed the EMT phenotypic changes, enhanced invasiveness, and mitochondrial functional alterations induced by INHBA overexpression. This establishes an “INHBA-Smad2/3-EMT/mitochondrial metabolism” molecular regulatory axis, offering a new perspective for a deeper understanding of the intrinsic link between aberrant mitochondrial energy metabolism and malignant phenotypes in ESCA, as well as providing an important theoretical basis and candidate molecular targets for prognostic assessment and the development of potential targeted therapeutic strategies.

## Figures and Tables

**Table 1:** Clinicopathological characteristics of TCGA-ESCA patients.

	Alive	Dead	Total
	(101)	(48)	(149)
Age			
Mean	62	63.7	62.5
Median	60	63.5	61
≤61	54 (53%)	23 (48%)	77 (52%)
>61	47 (47%)	25 (52%)	72 (48%)
Gender			
Female	17 (17%)	4 (8.3%)	21 (14%)
Male	84 (83%)	44 (92%)	128 (86%)
T			
T1	19 (19%)	10 (21%)	29 (19%)
T2	22 (22%)	11 (23%)	33 (22%)
T3	59 (58%)	24 (50%)	83 (56%)
T4	1 (1.0%)	2 (4%)	3 (2%)
TX	0 (0%)	1 (2%)	1 (1%)
N			
N0	45 (45%)	13 (27%)	58 (39%)
N1	38 (38%)	29 (61%)	67 (45%)
N2	10 (9%)	3 (6%)	13 (9%)
N3	5 (5.0%)	2 (4%)	7 (5%)
TX	3 (3.0%)	1 (2%)	4 (2%)
M			
M0	82 (81%)	35 (73%)	117 (79%)
M1	3 (3%)	7 (14%)	10 (6%)
MX	16 (16%)	6 (13%)	22 (15%)
Stage			
I	15 (15%)	3 (6%)	18 (12%)
II	45 (44%)	15 (31%)	60 (40%)
III	29 (29%)	20 (42%)	49 (33%)
IV	2 (2%)	6 (13%)	8 (5%)
X	10 (10%)	4 (8%)	14 (10%)

Note: TCGA: The Cancer Genome Atlas.

**Table 2:** GO enrichment analysis of differentially expressed genes between high- and low-risk groups.

ONTOLOGY	ID	Description	*p*.Adjust
BP	GO:0030198	extracellular matrix organization	2.181 × 10^−38^
BP	GO:0043062	extracellular structure organization	2.181 × 10^−38^
BP	GO:0045229	external encapsulating structure organization	2.374 × 10^−38^
BP	GO:0001503	ossification	6.934 × 10^−26^
BP	GO:0032963	collagen metabolic process	4.756 × 10^−22^
BP	GO:0030199	collagen fibril organization	5.818 × 10^−18^
BP	GO:0061448	connective tissue development	8.628 × 10^−16^
BP	GO:0060348	bone development	1.623 × 10^−12^
BP	GO:0051216	cartilage development	1.967 × 10^−12^
BP	GO:0031589	cell–substrate adhesion	1.951 × 10^−11^
CC	GO:0062023	collagen-containing extracellular matrix	8.137 × 10^−48^
CC	GO:0005788	endoplasmic reticulum lumen	4.498 × 10^−24^
CC	GO:0005581	collagen trimer	2.734 × 10^−17^
CC	GO:0098644	complex of collagen trimers	3.721 × 10^−15^
CC	GO:0005604	basement membrane	1.168 × 10^−14^
CC	GO:0030055	cell–substrate junction	1.484 × 10^−11^
CC	GO:0005925	focal adhesion	1.918 × 10^−11^
CC	GO:0005583	fibrillar collagen trimer	1.059 × 10^−9^
CC	GO:0098643	banded collagen fibril	1.059 × 10^−9^
CC	GO:0098636	protein complex involved in cell adhesion	1.391 × 10^−8^
MF	GO:0005201	extracellular matrix structural constituent	8.639 × 10^−36^
MF	GO:0005518	collagen binding	2.472 × 10^−19^
MF	GO:0005178	integrin binding	4.572 × 10^−15^
MF	GO:0030020	extracellular matrix structural constituent conferring tensile strength	2.454 × 10^−14^
MF	GO:0005539	glycosaminoglycan binding	1.221 × 10^−11^
MF	GO:0001968	fibronectin binding	1.221 × 10^−11^
MF	GO:0050840	extracellular matrix binding	1.800 × 10^−10^
MF	GO:0019838	growth factor binding	3.696 × 10^−9^
MF	GO:0004222	metalloendopeptidase activity	1.456 × 10^−7^
MF	GO:0008201	heparin binding	1.788 × 10^−7^

Note: GO: Gene Ontology; BP: Biological Process; MF: Molecular Function; CC: Cellular Component.

**Table 3:** KEGG pathway enrichment analysis of differentially expressed genes between high- and low-risk groups.

ID	Description	*p*.Adjust
hsa04974	Protein digestion and absorption	1.339 × 10^−9^
hsa05205	Proteoglycans in cancer	3.547 × 10^−9^
hsa04512	ECM-receptor interaction	7.759 × 10^−9^
hsa04510	Focal adhesion	1.255 × 10^−7^
hsa04933	AGE-RAGE signaling pathway in diabetic complications	2.973 × 10^−7^
hsa05146	Amoebiasis	1.881 × 10^−6^
hsa04380	Osteoclast differentiation	1.881 × 10^−6^
hsa05323	Rheumatoid arthritis	1.881 × 10^−6^
hsa05144	Malaria	0.0002
hsa04145	Phagosome	0.0002

Note: KEGG: Kyoto Encyclopedia of Genes and Genomes.

**Table 4:** GSEA results between high- and low-risk groups.

Description	Score	NES	*p*.Adjust
HALLMARK_EPITHELIAL_MESENCHYMAL_TRANSITION	0.829	2.754	1.14 × 10^−9^
HALLMARK_TNFA_SIGNALING_VIA_NFKB	0.679	2.259	1.14 × 10^−9^
HALLMARK_INFLAMMATORY_RESPONSE	0.662	2.200	1.14 × 10^−9^
HALLMARK_APICAL_JUNCTION	0.592	1.969	1.14 × 10^−9^
HALLMARK_HYPOXIA	0.589	1.959	1.14 × 10^−9^
HALLMARK_MYOGENESIS	0.581	1.931	5.38 × 10^−9^
HALLMARK_ALLOGRAFT_REJECTION	0.570	1.893	1.21 × 10^−8^
HALLMARK_OXIDATIVE_PHOSPHORYLATION	−0.455	−1.840	3.66 × 10^−7^
HALLMARK_INTERFERON_GAMMA_RESPONSE	0.545	1.812	3.66 × 10^−7^
HALLMARK_IL2_STAT5_SIGNALING	0.536	1.781	1.21 × 10^−6^

Note: GSEA: Gene Set Enrichment Analysis.

**Table 5:** Univariate Cox regression analysis of overall survival in TCGA-ESCA patients.

	Hazard Ratio (HR)	Lower 95%CI	Upper 95%CI	*p* Value
Stage	2.14	0.579	7.89	0.00308
TNM_M	3.28	1.42	7.57	0.00541
riskScore	2.72	1.26	5.89	0.0112
TNM_N	2.22	1.09	4.51	0.156
TNM_T	1.2	0.508	2.85	0.331
Age	0.997	0.975	1.02	0.789

Note: TNM_M: Tumor Node Metastasis_Metastasis; TNM_N: Tumor Node Metastasis_Node; TNM_T: Tumor Node Metastasis_Tumor.

**Table 6:** Multivariate Cox regression analysis of independent prognostic factors.

	Hazard Ratio (HR)	Lower 95%CI	Upper 95%CI	*p* Value
riskScore	3.02	1.34	6.82	0.008
TNM_MM1	2.94	1.26	6.89	0.013

## Data Availability

All data generated or analyzed during this study are included in this article and/or its Supplementary Material files. Further enquiries can be directed to the corresponding author.

## Supplementary Materials

The following supporting information can be downloaded at https://www.mdpi.com/article/10.3390/biom16091367/s1, Supplementary Material S1: mitochondrial energy metabolism-related genes; Supplementary Material S2: MEMRGs retained after intersection with the TCGA-ESCA expression profile; Supplementary Material S3: DEGs between the high- and low-MEMS groups; Supplementary Material S4: WGCNA module genes associated with MEMS; Supplementary Material S5: key genes related to mitochondrial energy metabolism; Supplementary Material S6: correlation analysis of key genes; Supplementary Material S7: DEGs between the high- and low-risk groups; Supplementary Material S8: miRNA–mRNA regulatory-network data; Supplementary Material S9: TIDE prediction results. Supplementary Material S10: Western Blot Source Images. Figure S1. Overall workflow of the bioinformatics analyses in this study. Figure S2. Boxplots of expression profiles before and after normalization correction in the GSE53625 dataset. Figure S3. GSEA enrichment analysis plots based on the TCGA-ESCA dataset. Figure S4. Immune infiltration analysis based on the TCGA-ESCA dataset. Figure S5. MEMS risk score and genomic alterations and immunotherapy response assessment in TCGA-ESCA patients. Figure S6. Construction and evaluation of a risk score-based clinical prediction model. Figure S7. INHBA expression levels in esophageal squamous cell carcinoma cells and construction of intervention models. Figure S8. Effects of INHBA on the short-term proliferative capacity of esophageal squamous cell carcinoma cells. Figure S9. Effects of INHBA on the wound-healing migration ability of esophageal squamous cell carcinoma cells. Figure S10. Effects of INHBA on oxidative stress levels in esophageal squamous cell carcinoma cells. Figure S11. Proliferation-controlled migration assays validate the effect of INHBA on esophageal squamous cell carcinoma cell motility. Figure S12. INHBA knockdown alters chemotherapy sensitivity in esophageal cancer cells.

## Abbreviations

AUC	Area Under the Curve
ATP	Adenosine Triphosphate
BP	Biological Process
CC	Cellular Component
CCK-8	Cell Counting Kit-8
CNV	Copy Number Variation
DEGs	Differentially Expressed Genes
EMT	Epithelial–Mesenchymal Transition
ESCA	Esophageal Cancer
FBS	Fetal Bovine Serum
FC	Fold Change
FDR	False Discovery Rate
GEO	Gene Expression Omnibus
GO	Gene Ontology
GSEA	Gene Set Enrichment Analysis
HR	Hazard Ratio
KEGG	Kyoto Encyclopedia of Genes and Genomes
LASSO	Least Absolute Shrinkage and Selection Operator
MEMRGs	Mitochondrial Energy Metabolism-Related Genes
MEMS	Mitochondrial Energy Metabolism Score
MEs	Module Eigengenes
MF	Molecular Function
NES	Normalized Enrichment Score
OCR	Oxygen Consumption Rate
OS	Overall Survival
PBS	Phosphate-Buffered Saline
PCA	Principal Component Analysis
PPI	Protein–protein Interaction
qRT-PCR	Quantitative Real-Time Polymerase Chain Reaction
ROC	Receiver Operating Characteristic
ROS	Reactive Oxygen Species
ssGSEA	Single-Sample Gene Set Enrichment Analysis
TCGA	The Cancer Genome Atlas
TIDE	Tumor Immune Dysfunction and Exclusion
TME	Tumor Microenvironment
WGCNA	Weighted Gene Co-Expression Network Analysis
