# Supplementary material for: Construction of an Esophageal Cancer Prognostic Model Based on Mitochondrial Energy Metabolism-Related Genes and Mechanism of INHBA in Promoting Malignant Progression via the Smad2/3 Pathway
Source: Biomolecules. 2026 Sep 20;16(9):1367. doi: 10.3390/biom16091367 (PMC13604558; doi:10.3390/biom16091367)
Supplement: Supplementary file 1 [file biomolecules-16-01367-s001.zip › Supplementary Material S10. Western Blot Source Images.pdf]

Western Blot Source Images

Figure 8A — E-cadherin — condition 1

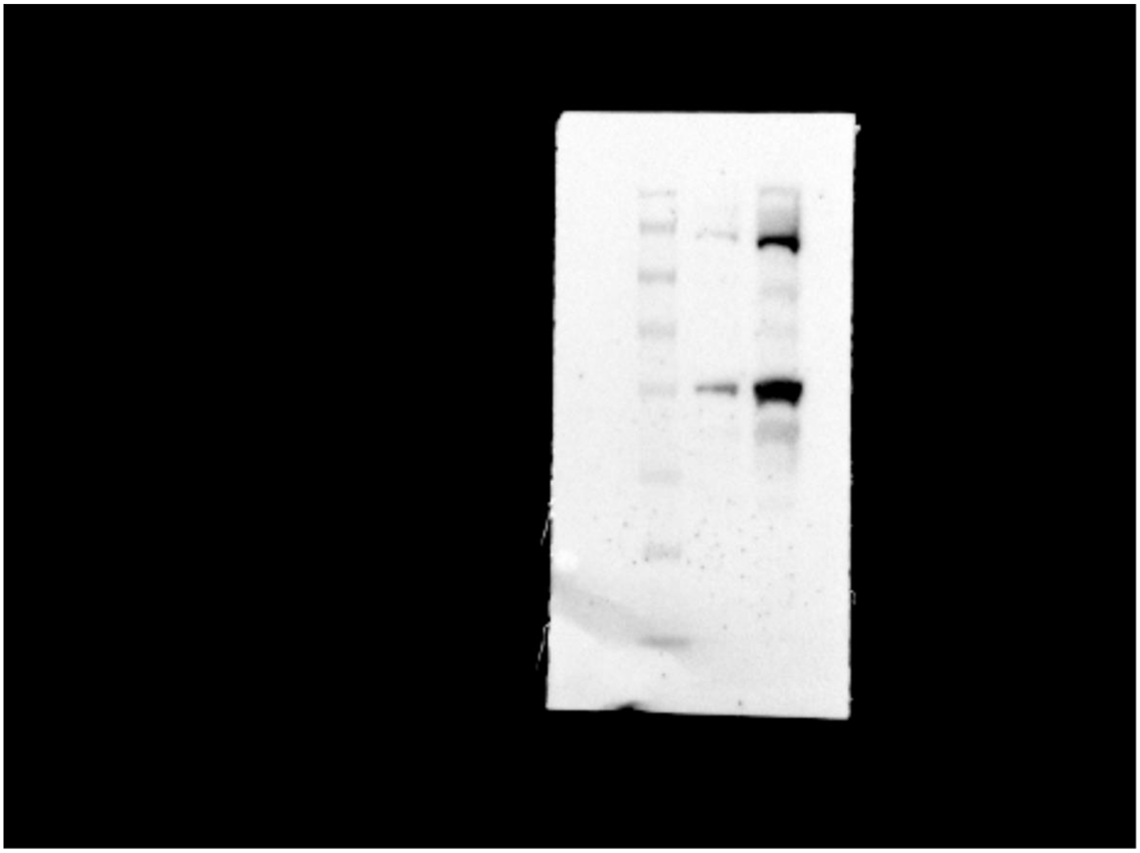

Figure 8A — E-cadherin — condition 2

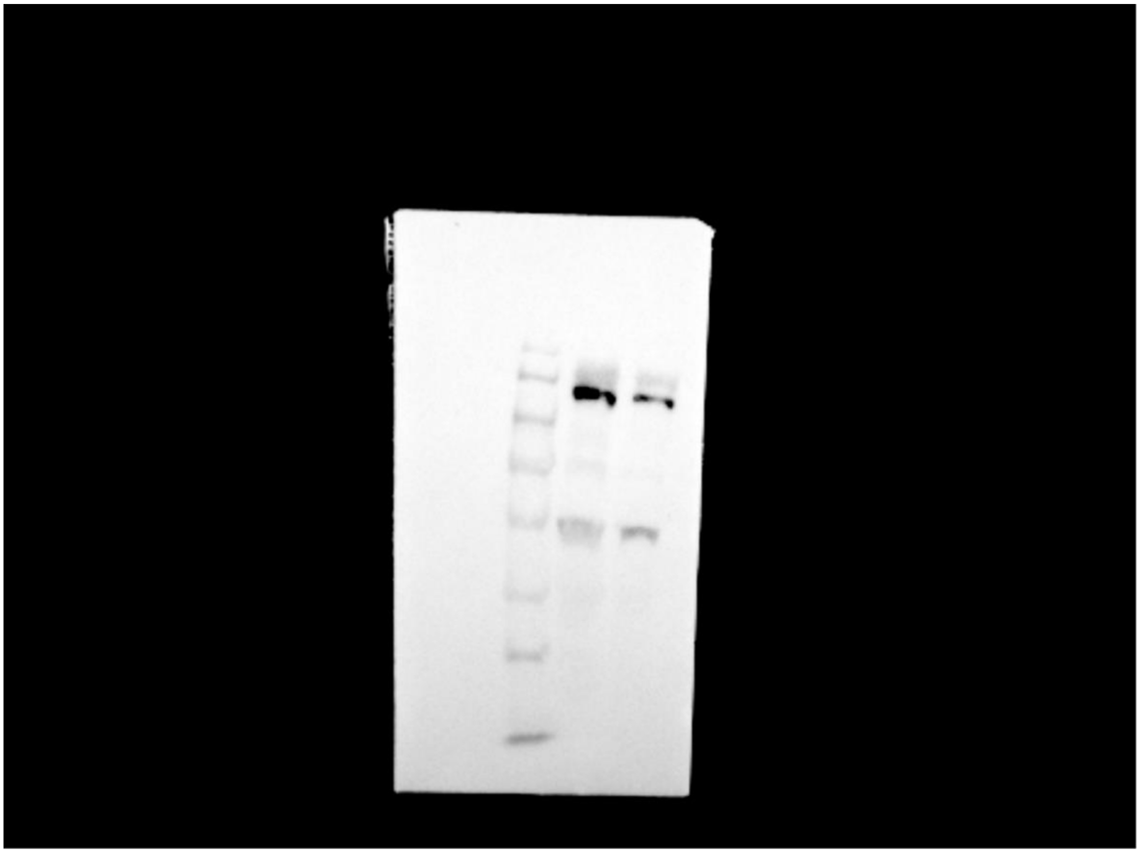

Figure 8A — N-cadherin — condition 1

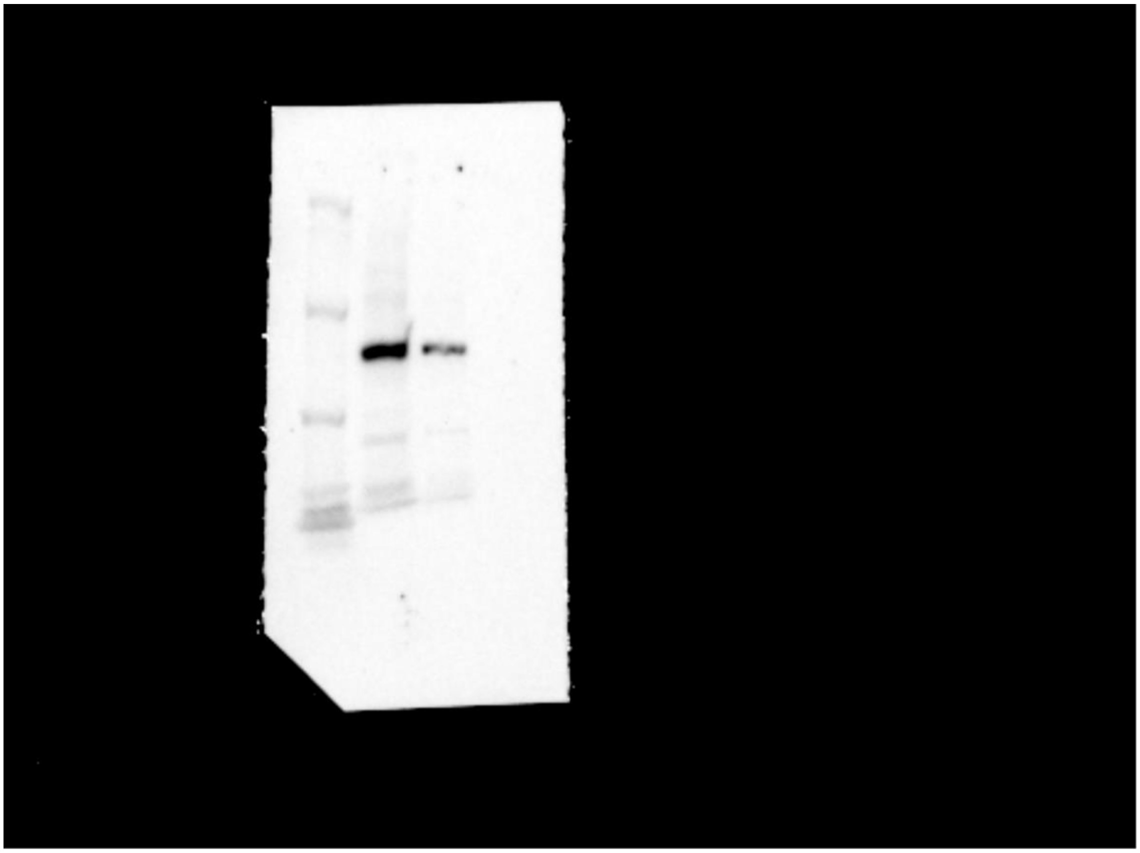

Figure 8A — N-cadherin — condition 2

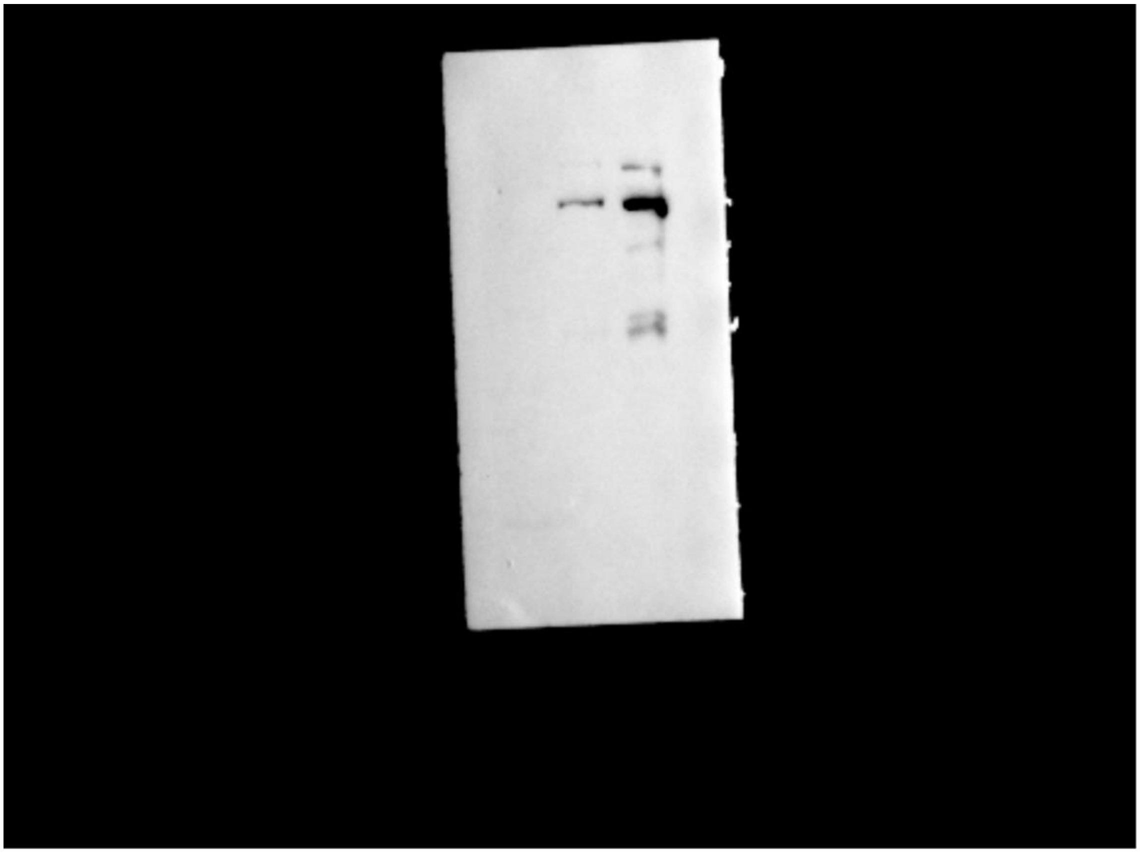

Figure 8A — Vimentin — condition 1

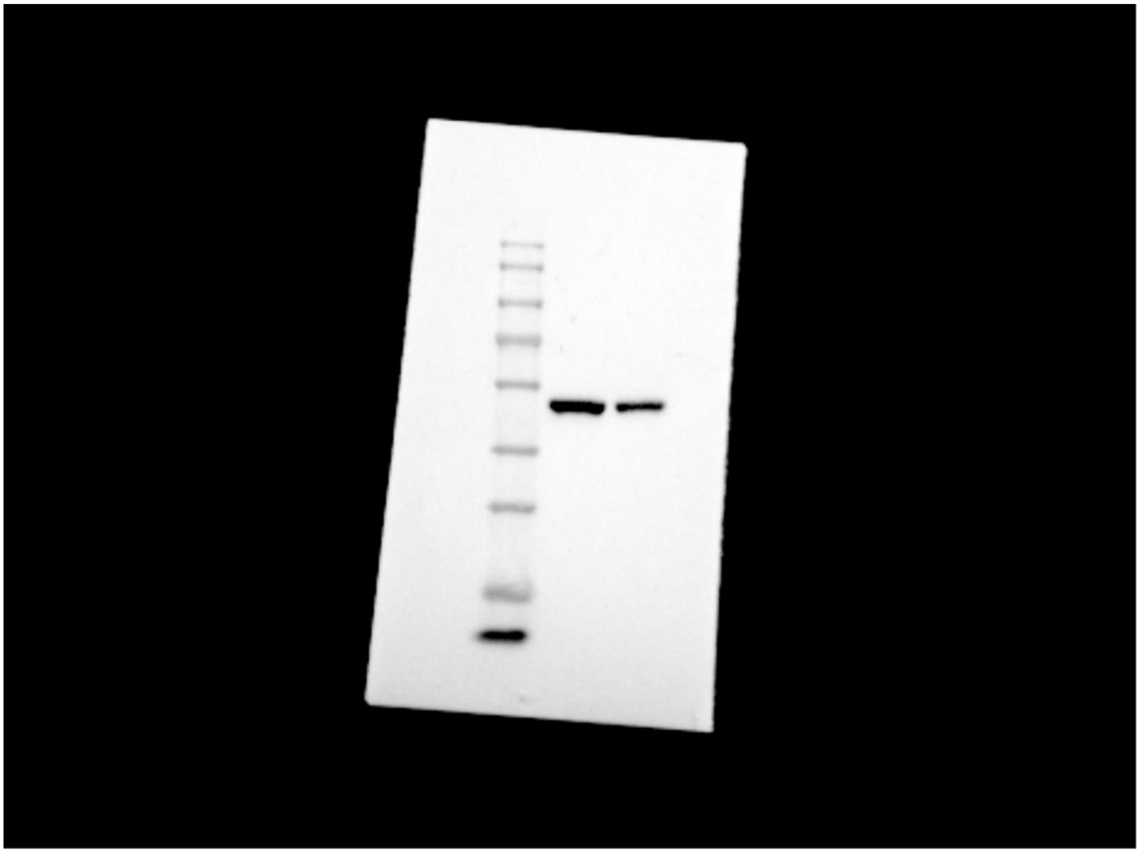

Figure 8A — Vimentin — condition 2

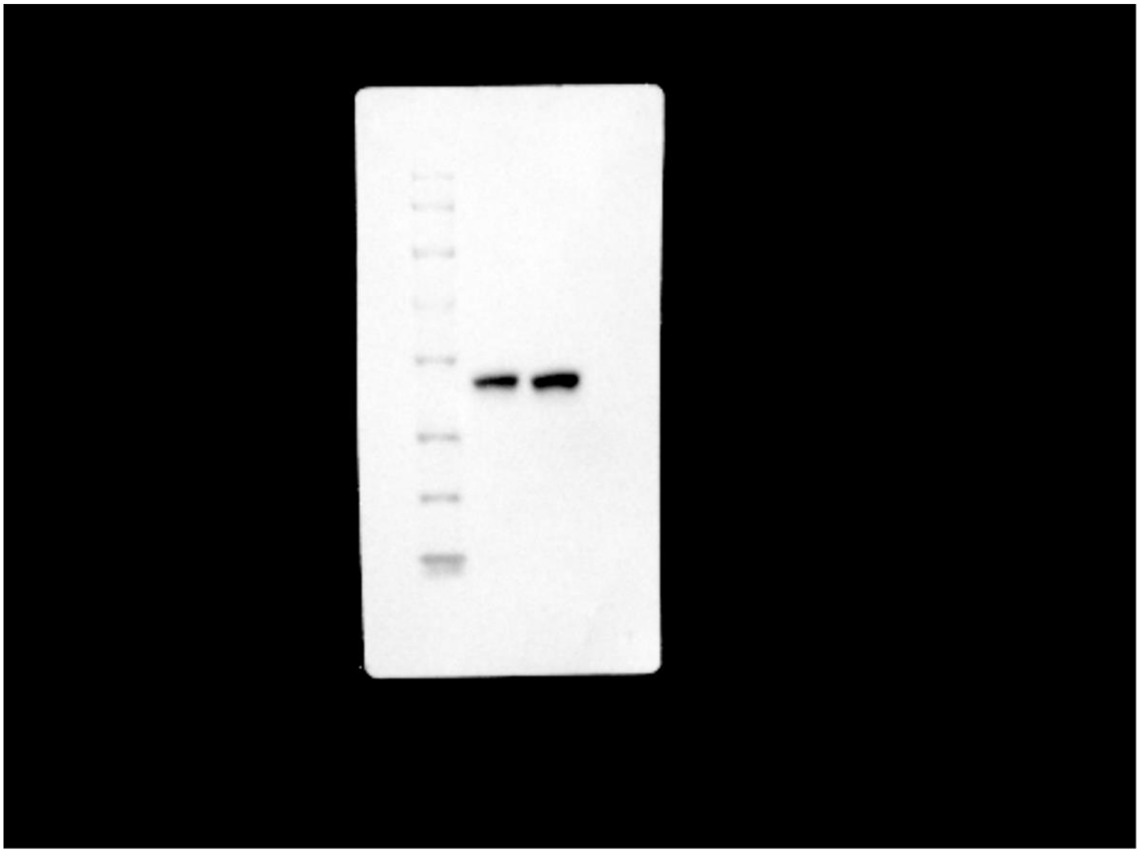

Figure 8A — Snail — condition 1

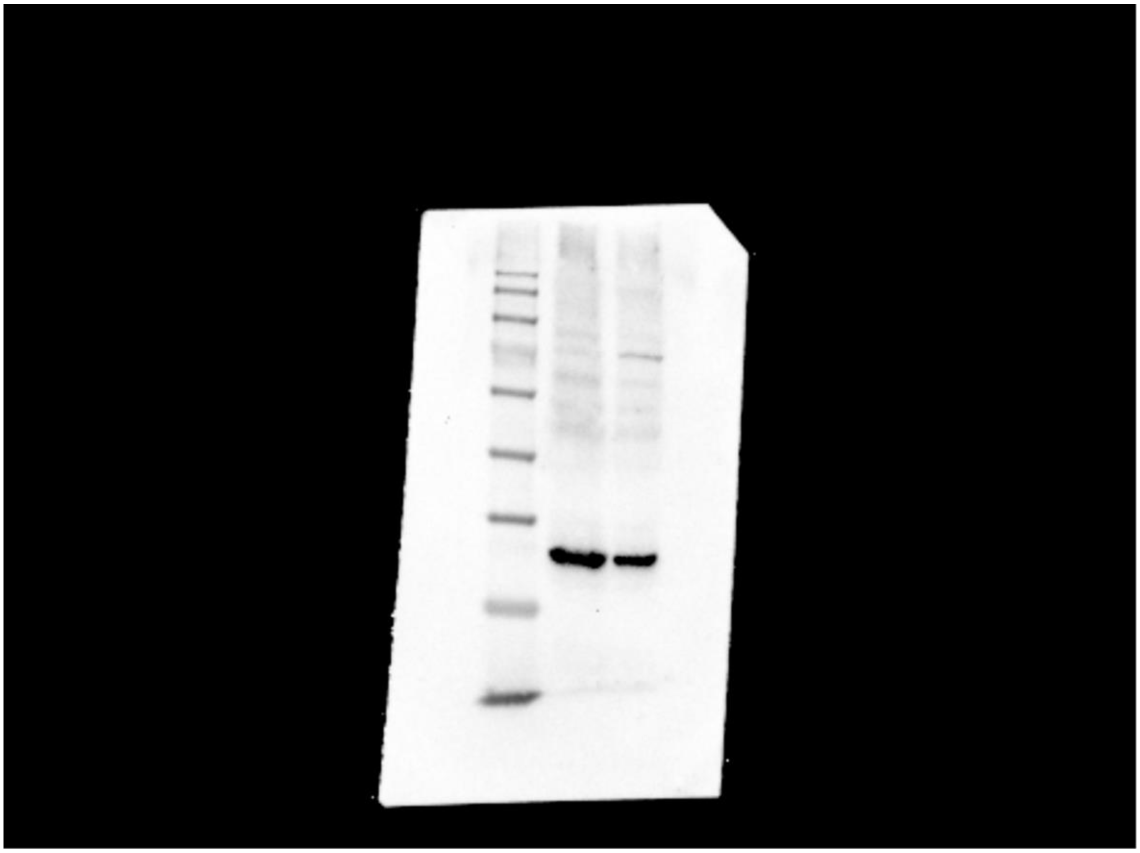

Figure 8A — Snail — condition 2

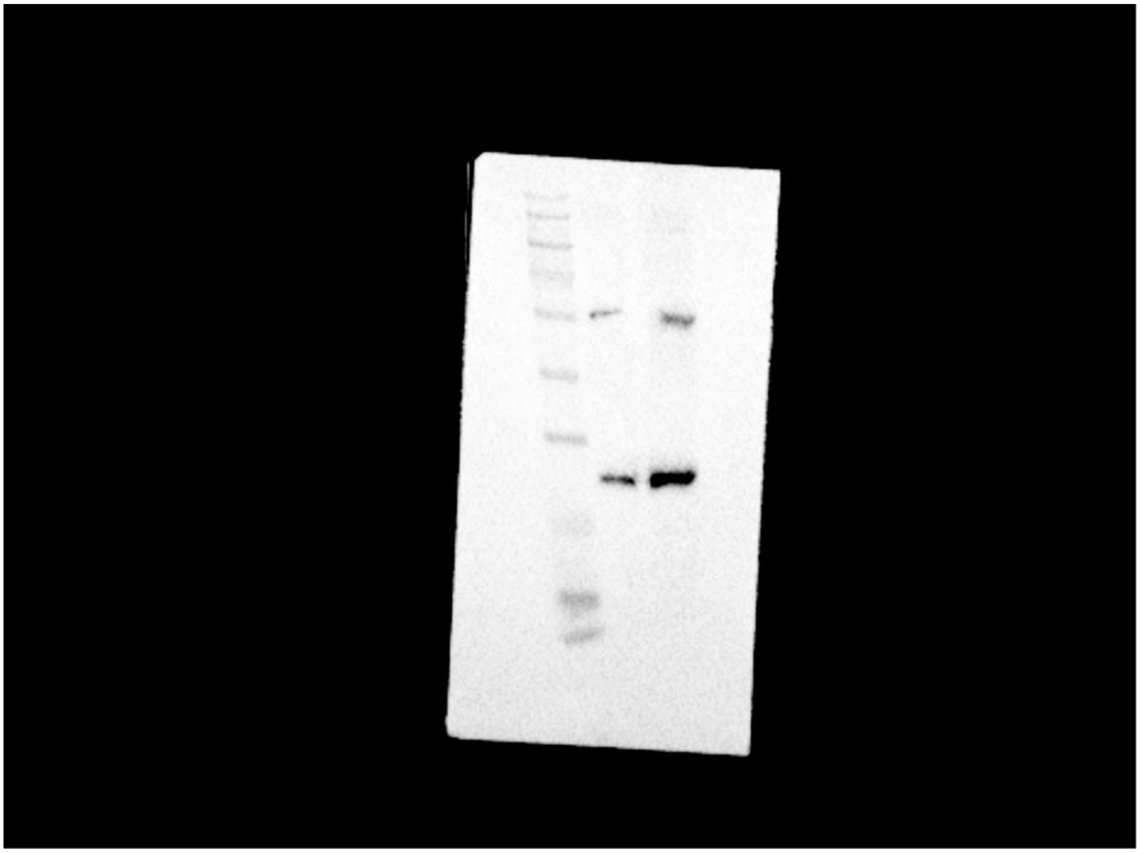

Figure 8A — p-Smad2 — condition 1

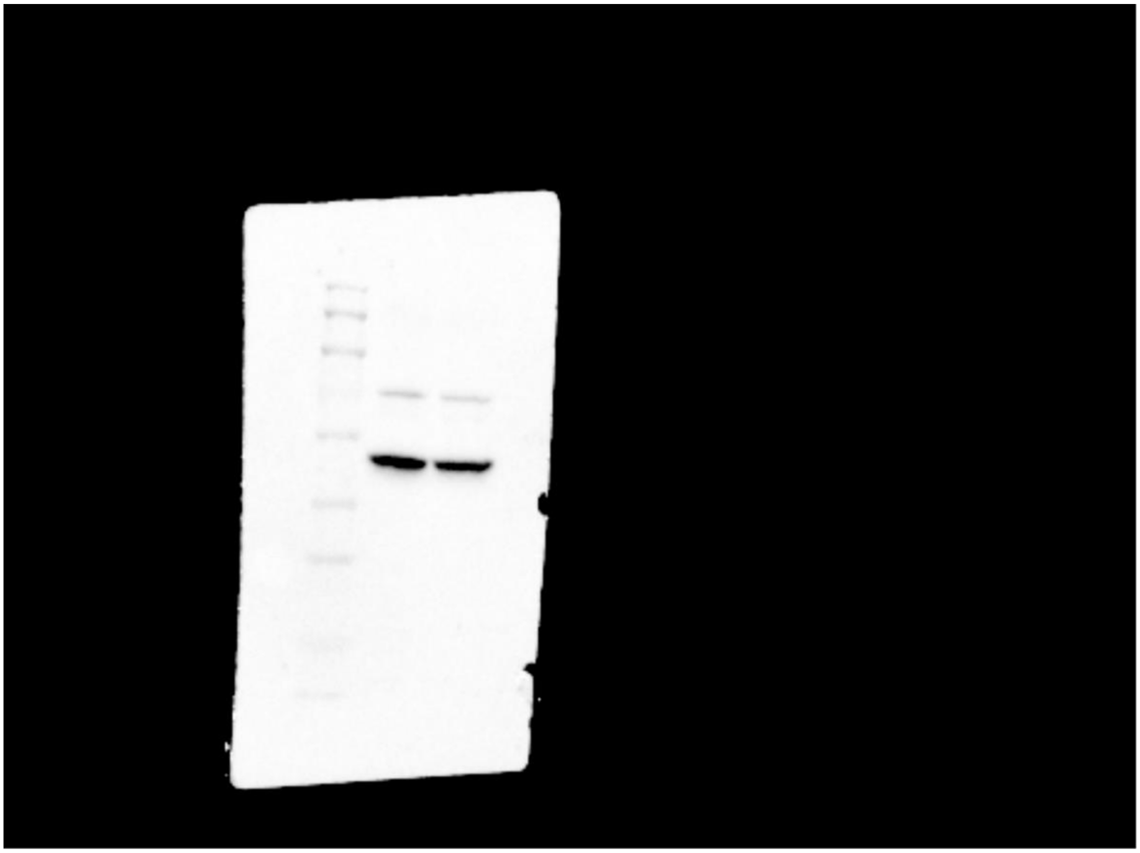

Figure 8A — p-Smad2 — condition 2

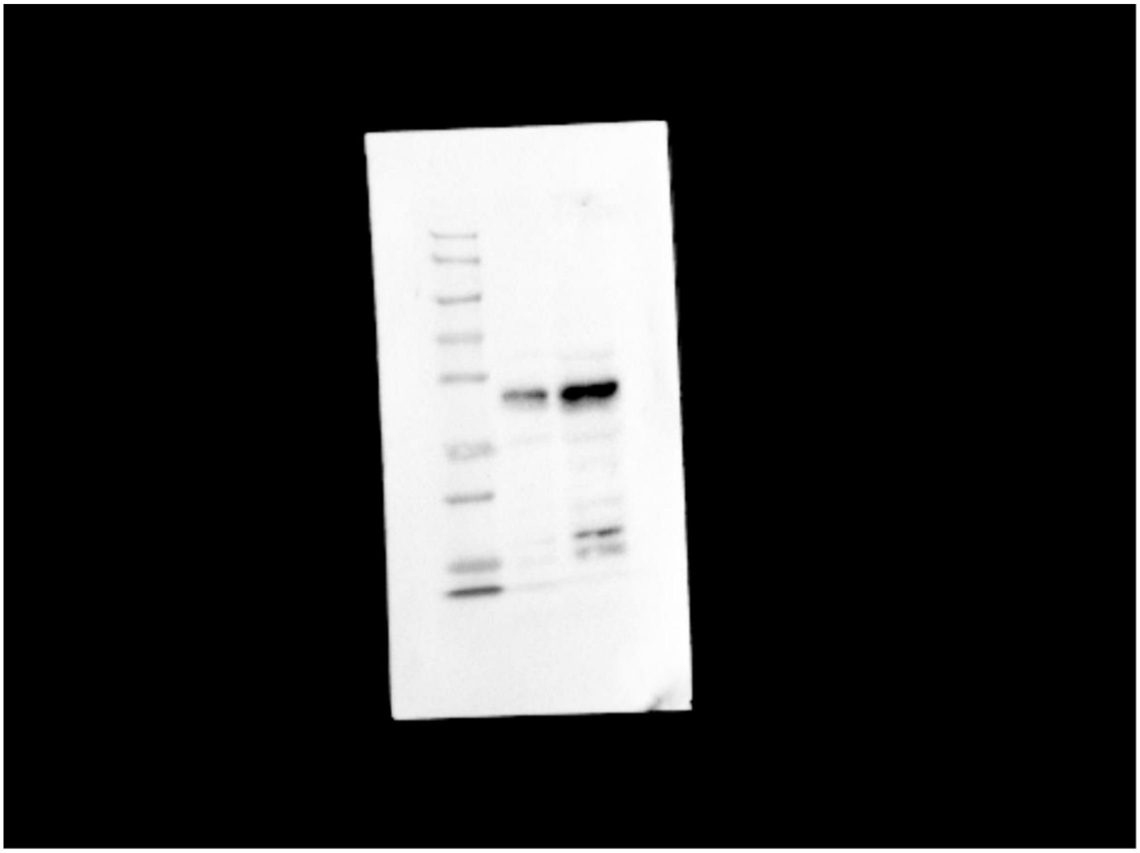

Figure 8A — Smad2/3 — condition 1

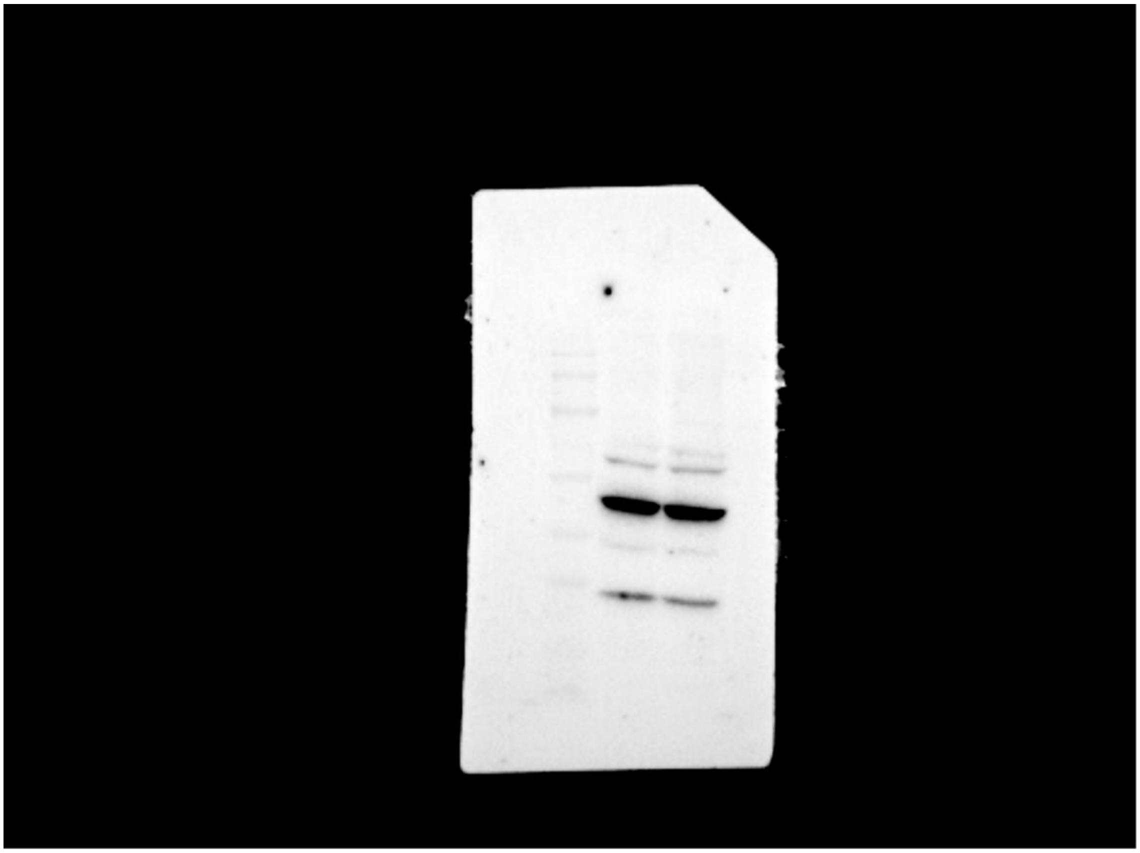

Figure 8A — Smad2/3 — condition 2

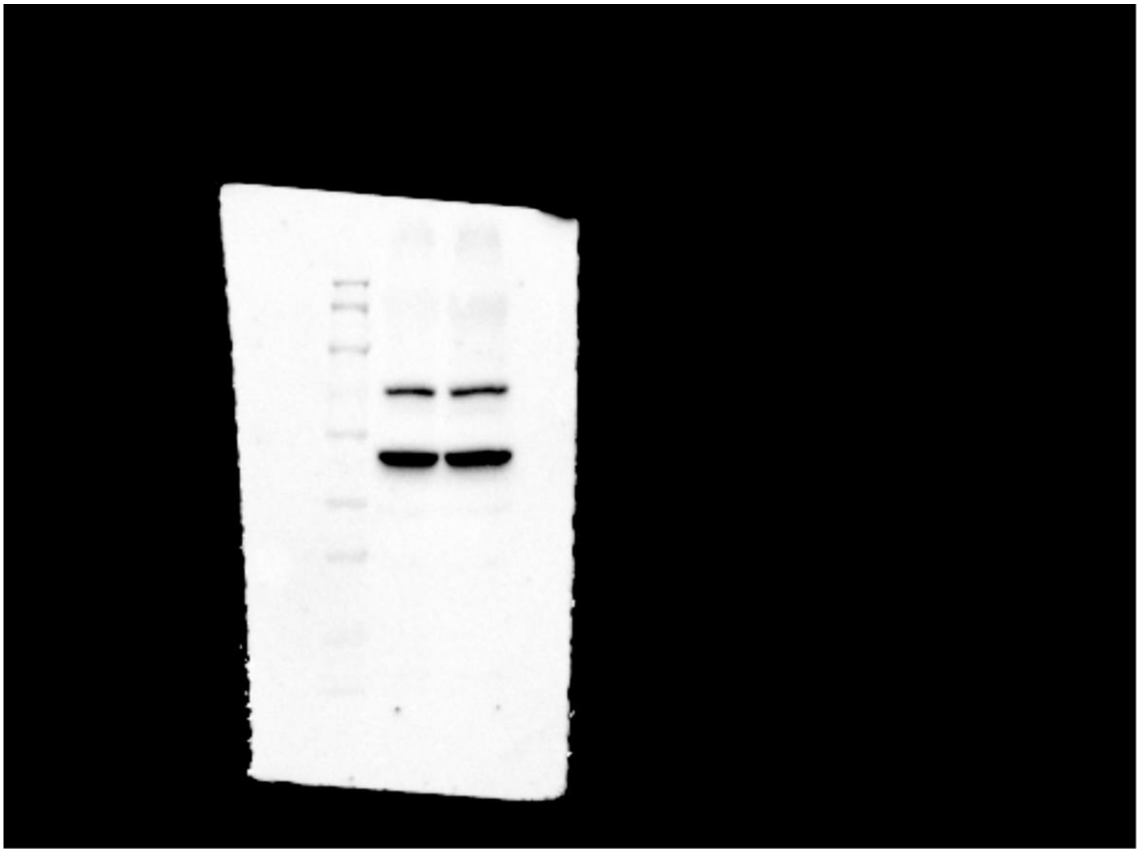

Figure 8A — p-Smad3 — condition 1

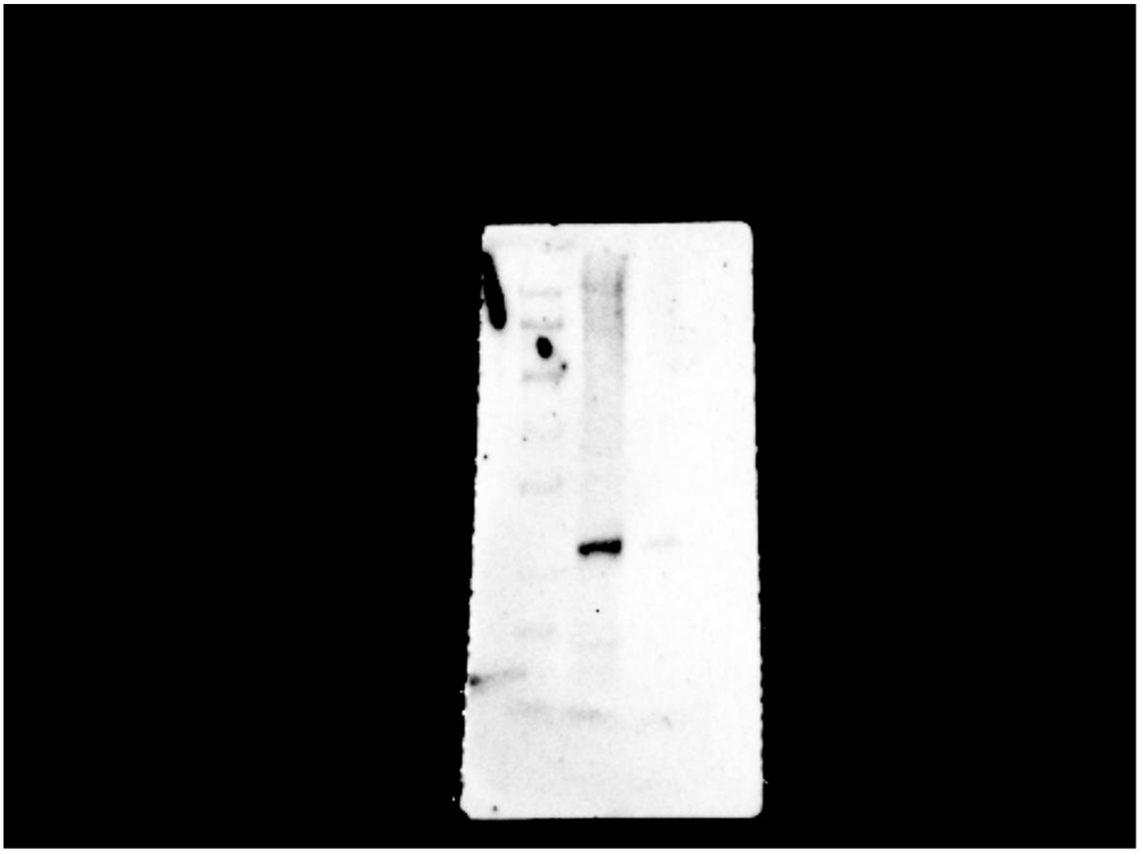

Figure 8A — p-Smad3 — condition 2

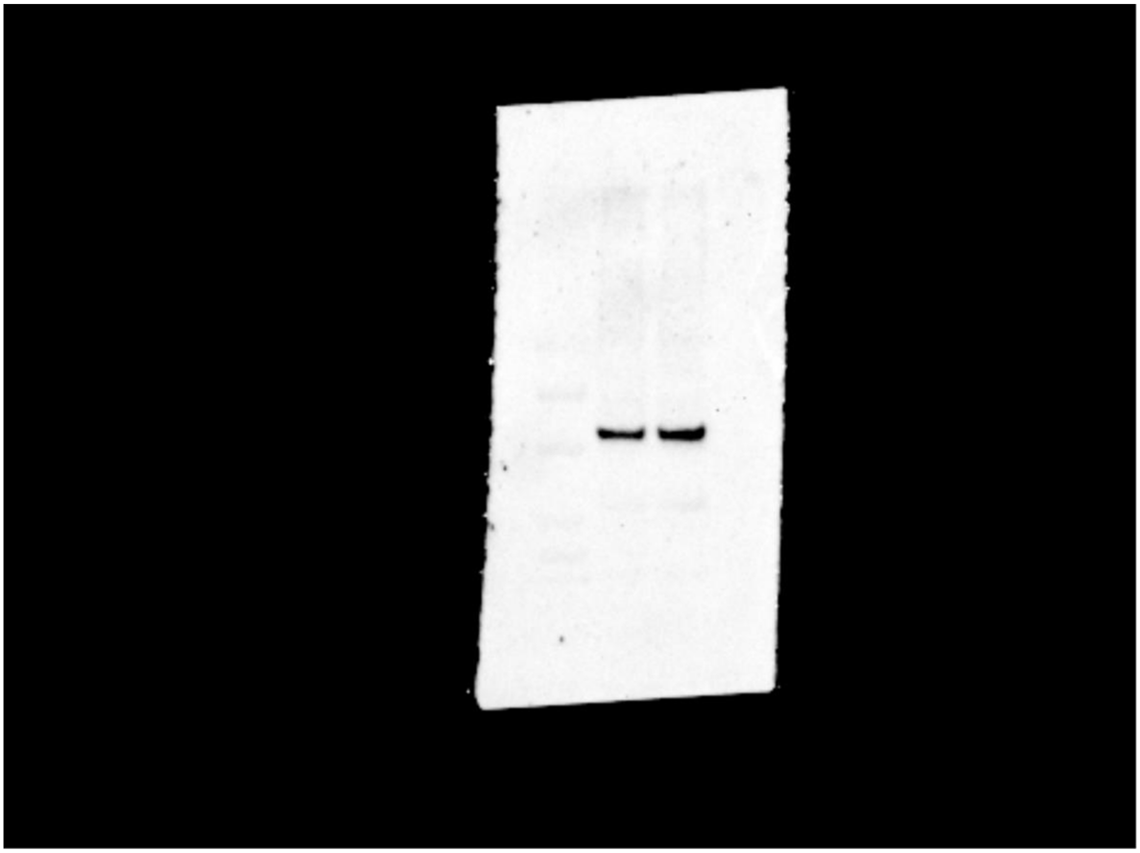

Figure 8A — GAPDH — condition 1

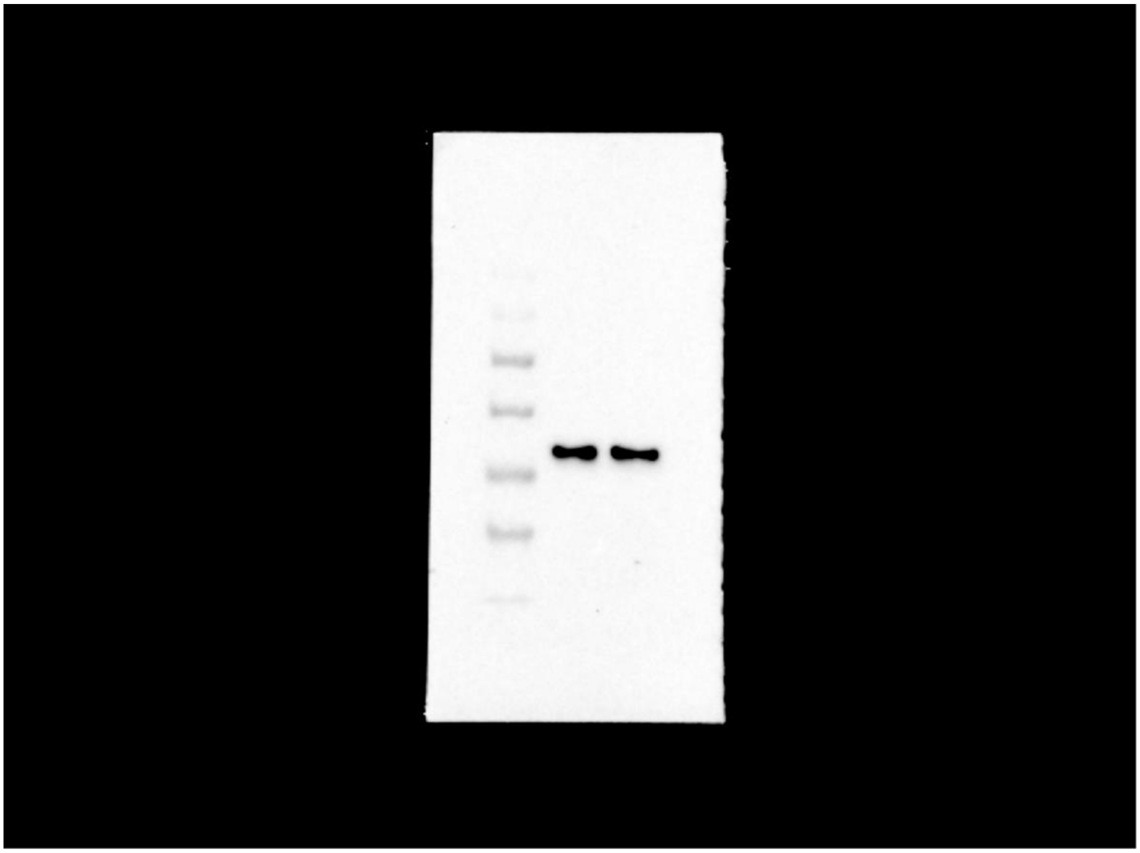

Figure 8A — GAPDH — condition 2

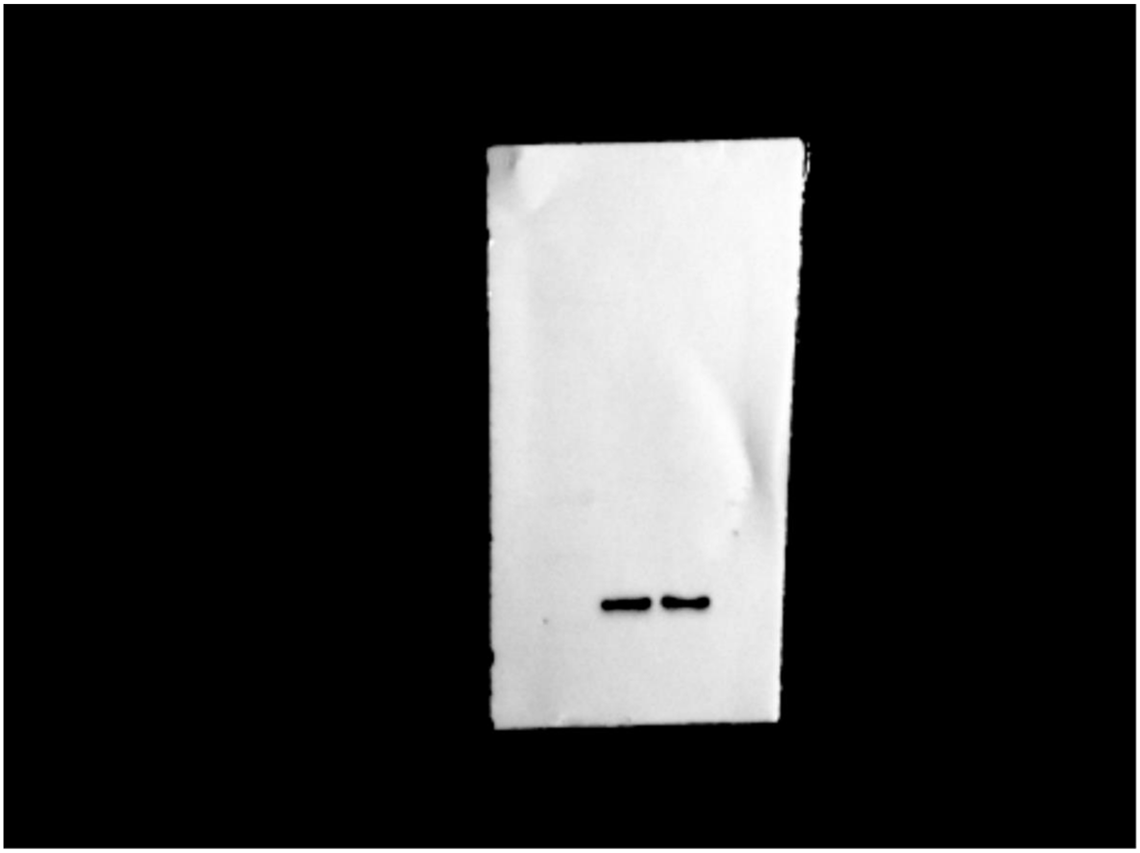

Figure 8I — E-cadherin — source image

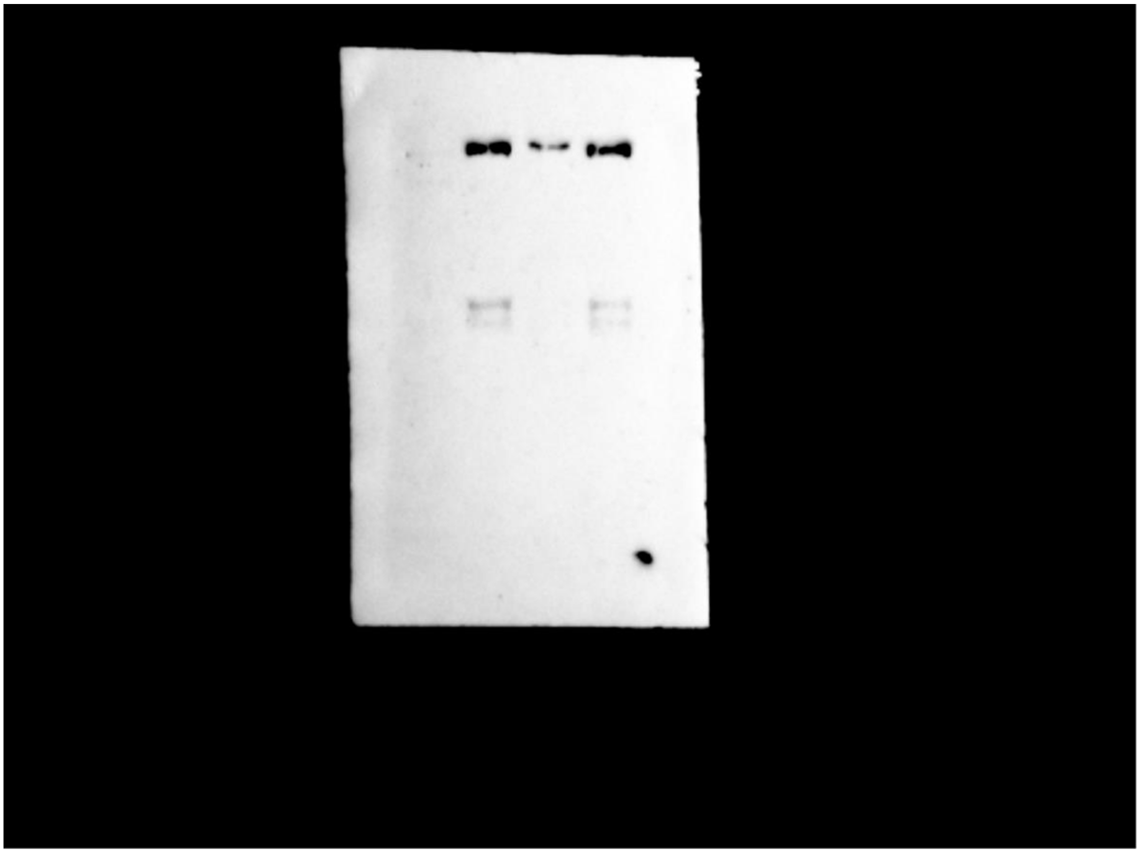

Figure 8I — N-cadherin — source image

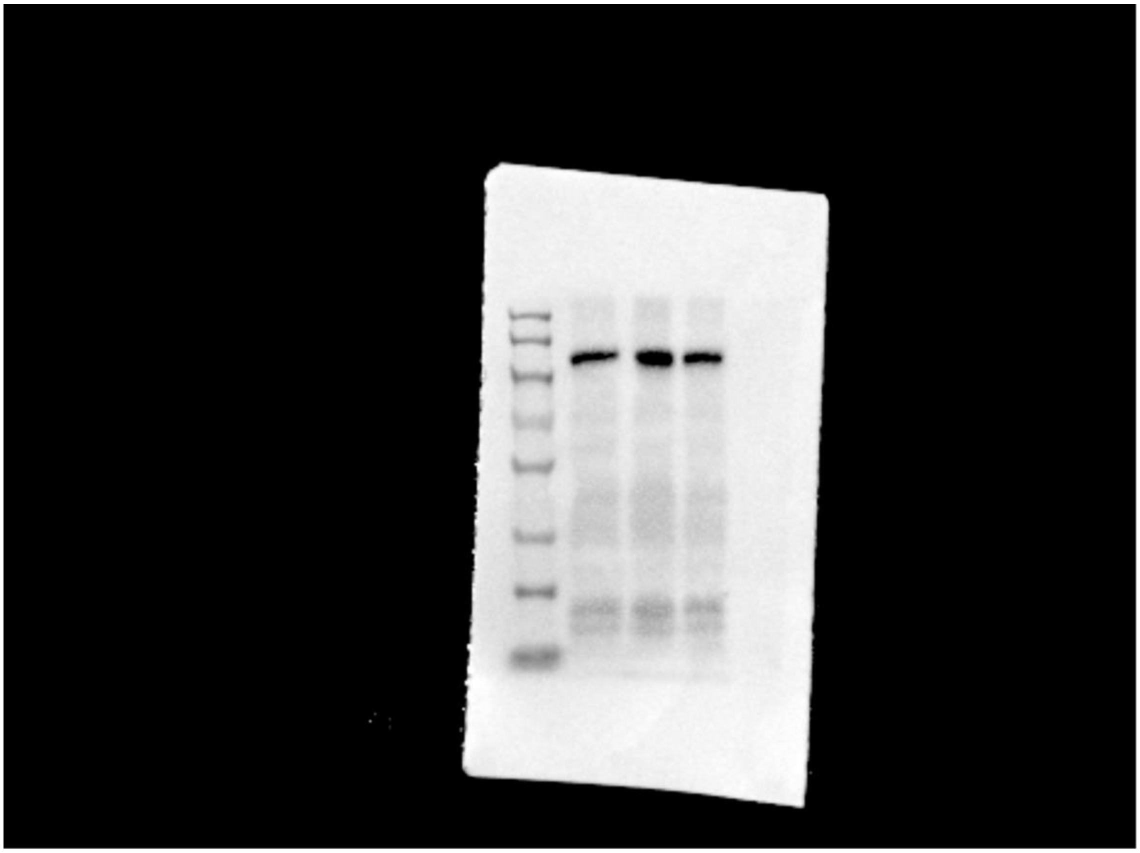

Figure 8I — Vimentin — source image

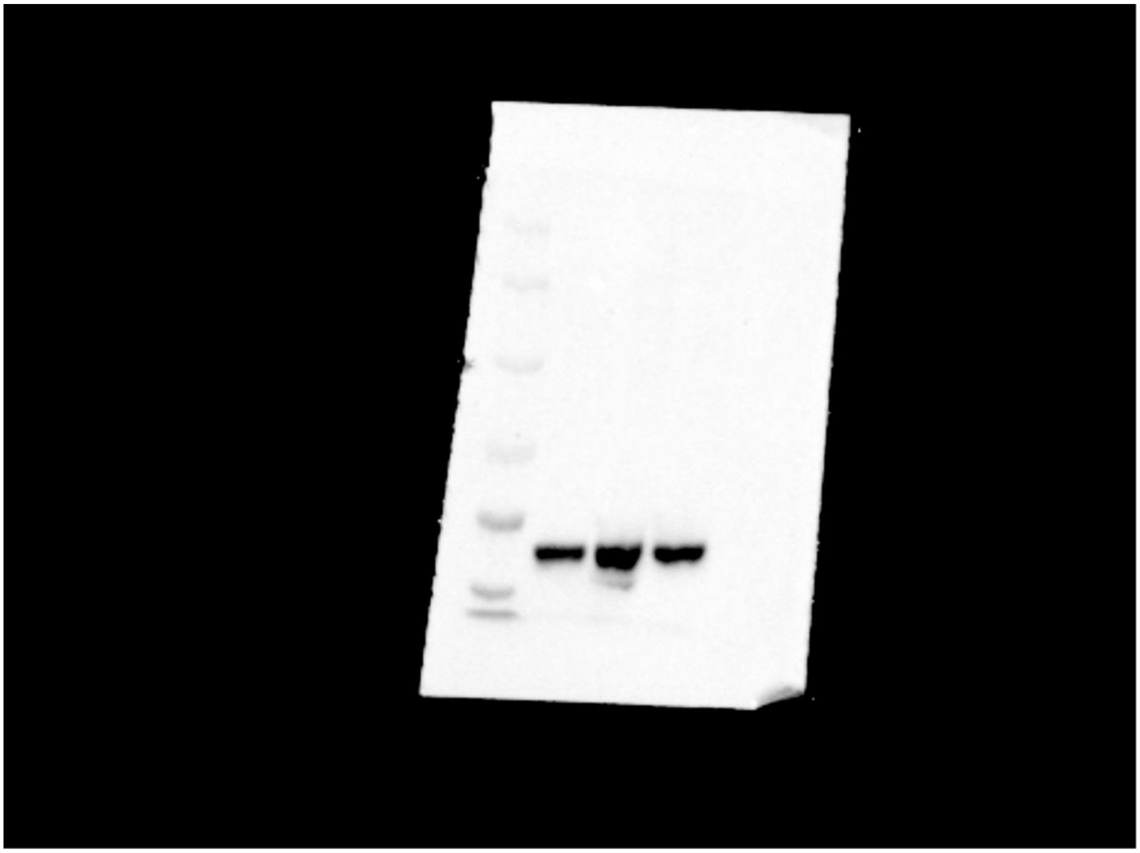

Figure 8I — Snail — source image

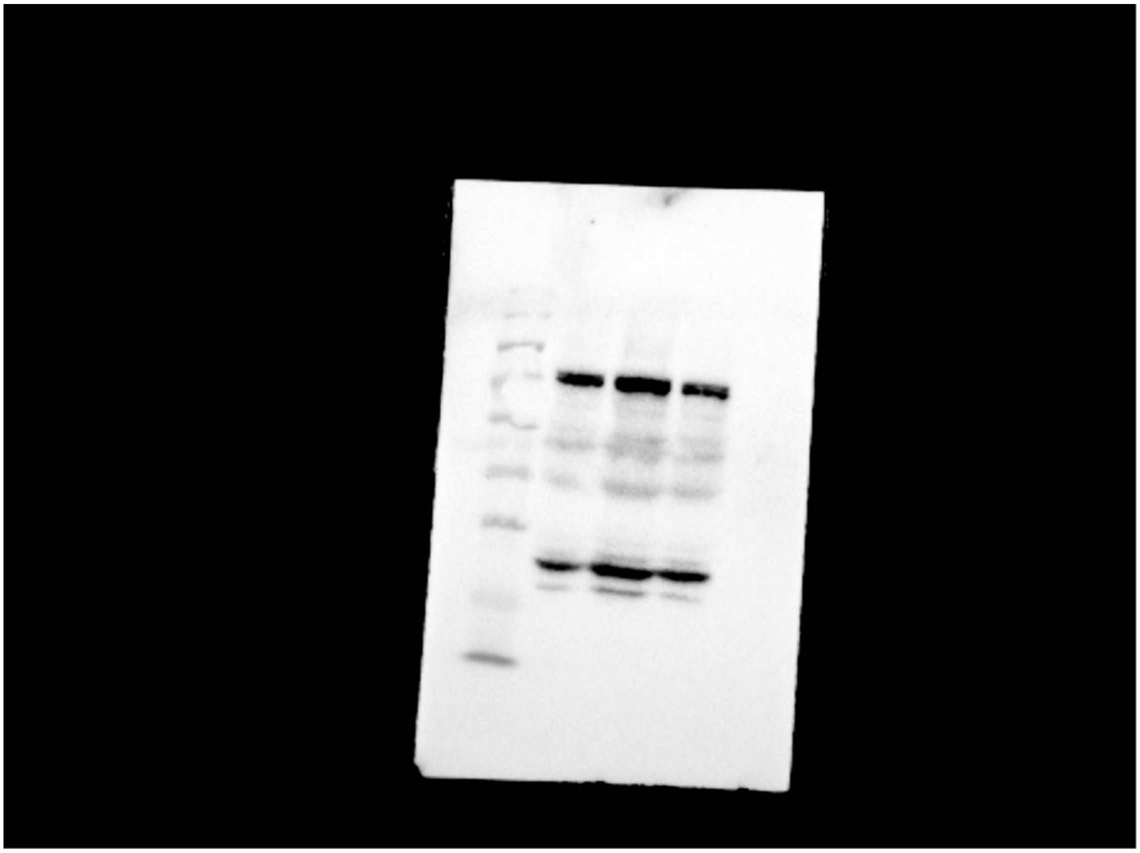

Figure 8I — p-Smad2 — source image

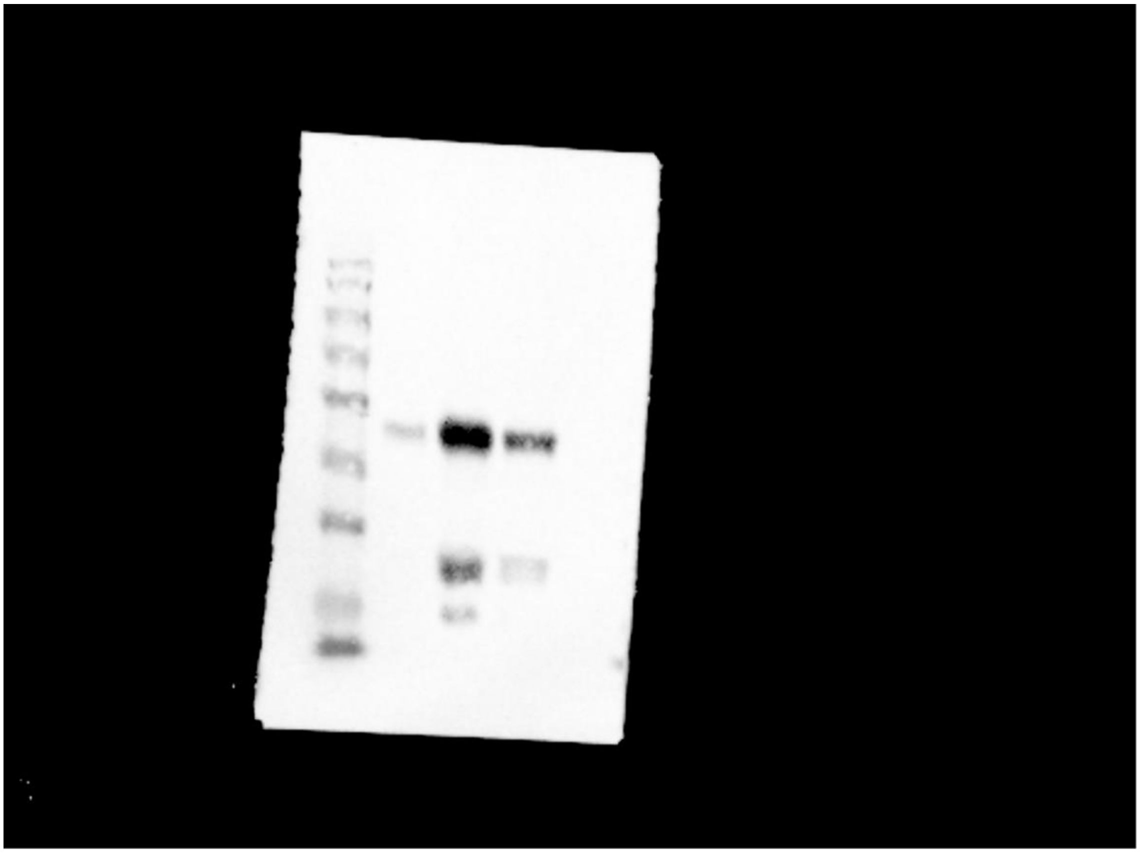

Figure 8I — Smad2/3 — source image

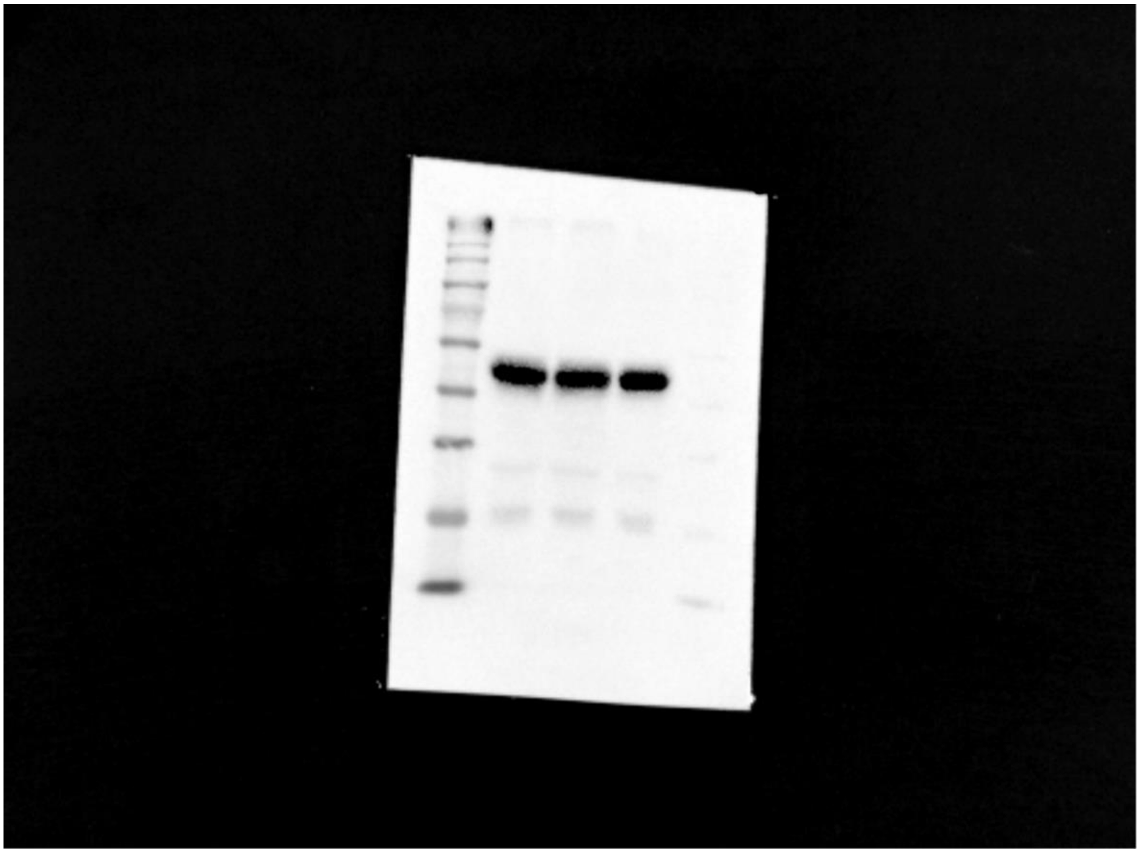

Figure 8I — p-Smad3 — source image

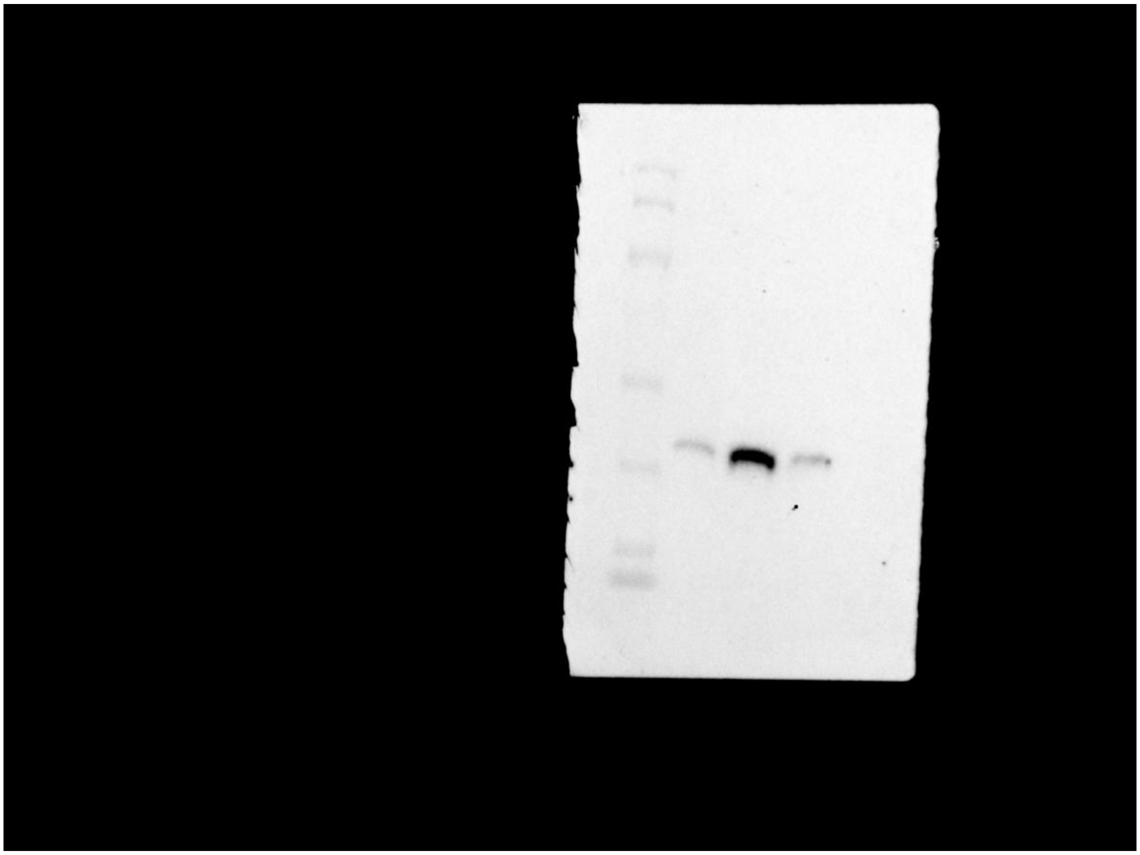

Figure 8I — GAPDH — source image

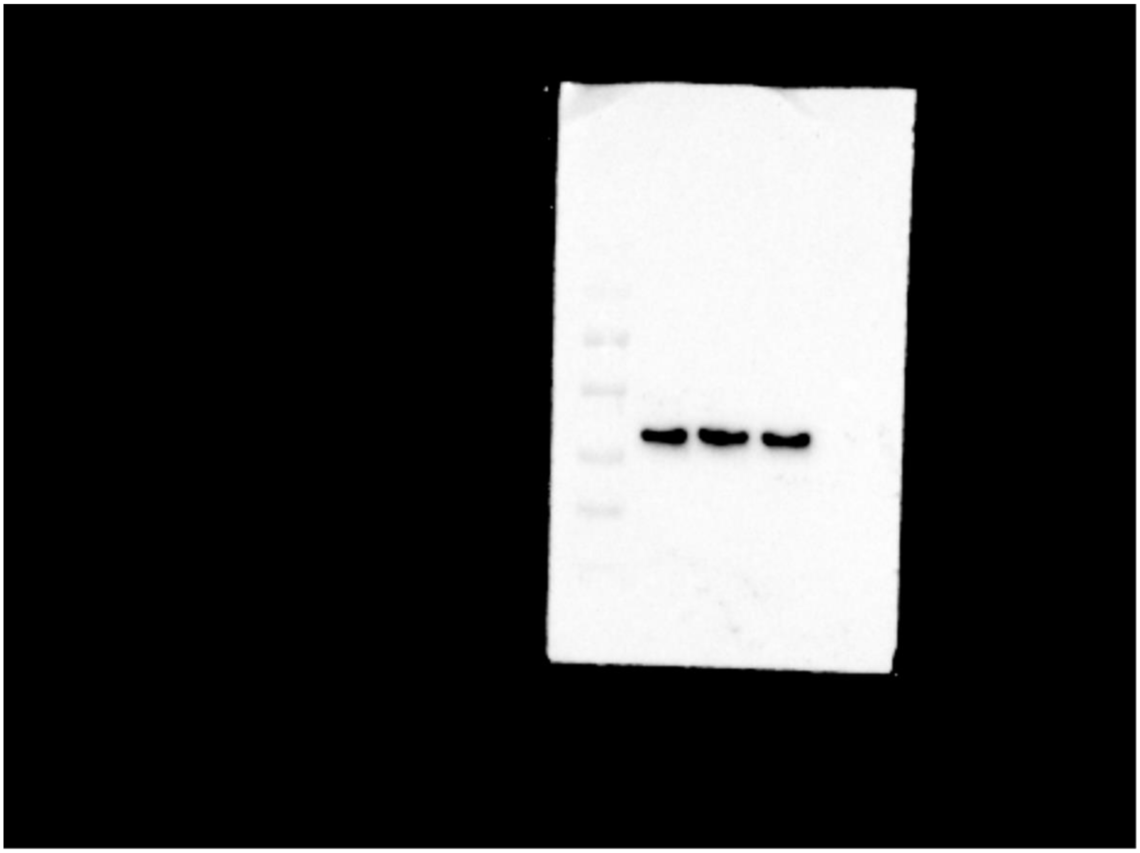

Western Blot Source Images

Figure 9C

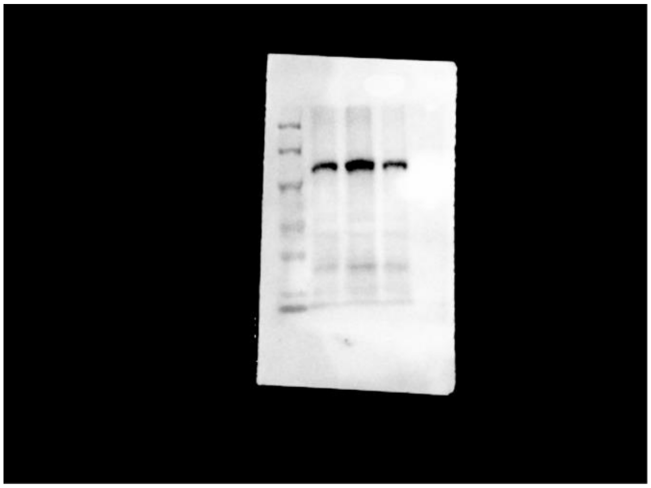

Figure 9C-1(1)

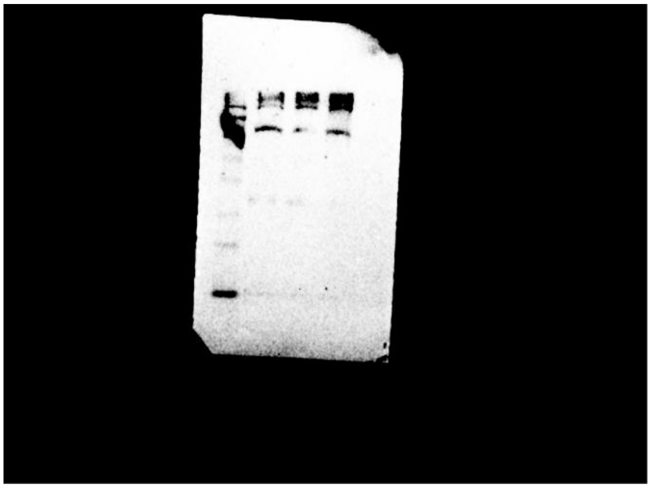

Figure 9C-1(2)

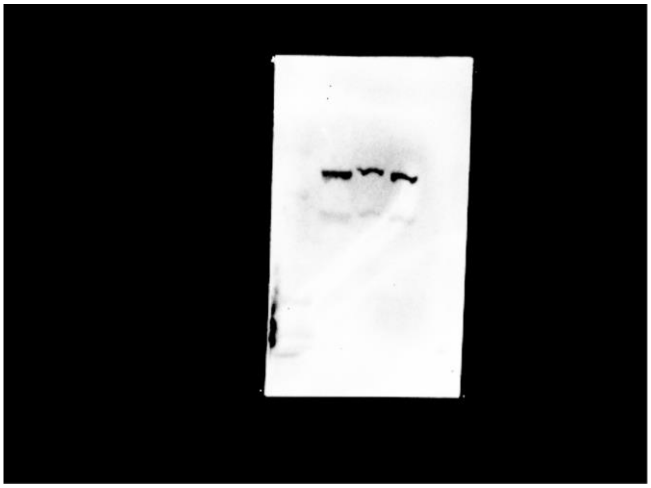

Figure 9C-2(1)

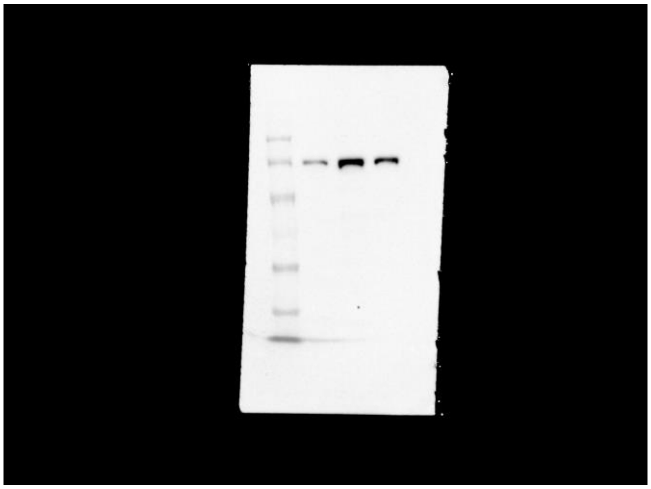

Figure 9C-2(2)

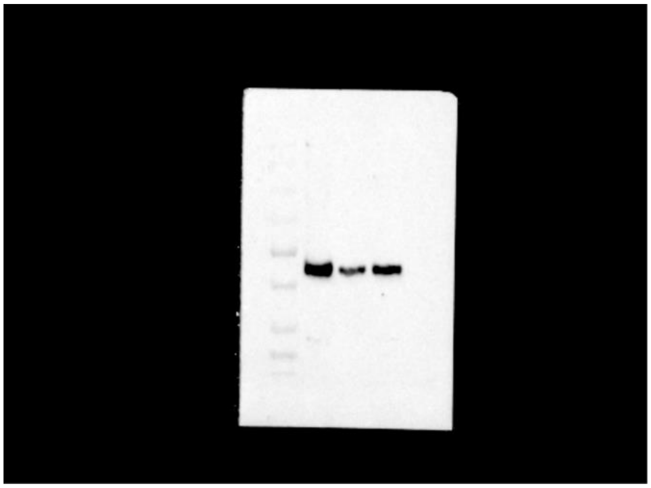

Figure 9C-3(1)

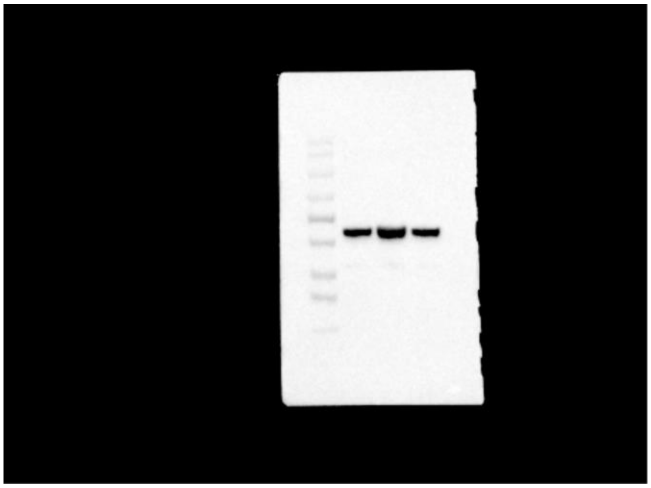

Figure 9C-3(2)

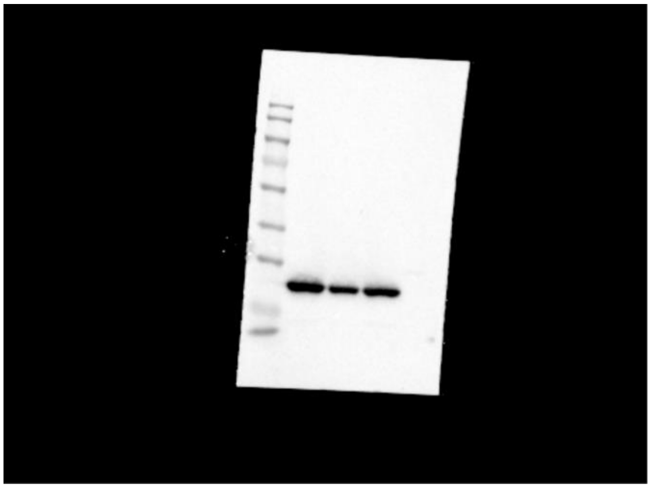

Figure 9C-4(1)

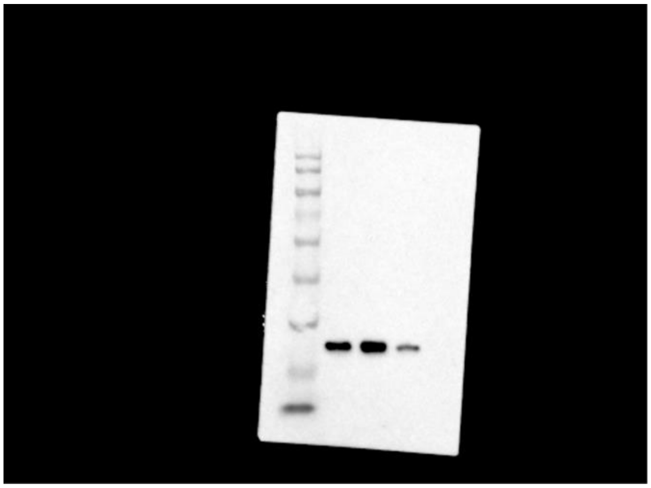

Figure 9C-4(2)

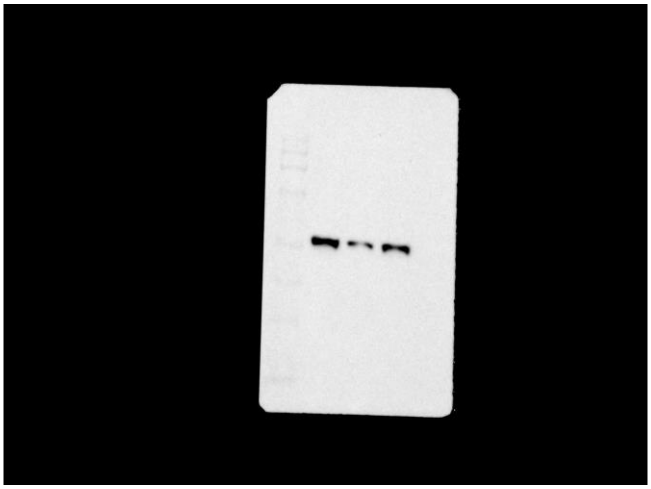

Figure 9C-5(1)

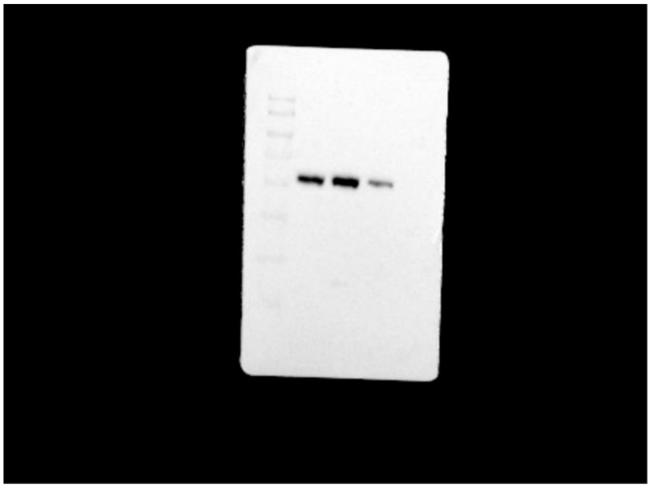

Figure 9C-5(2)

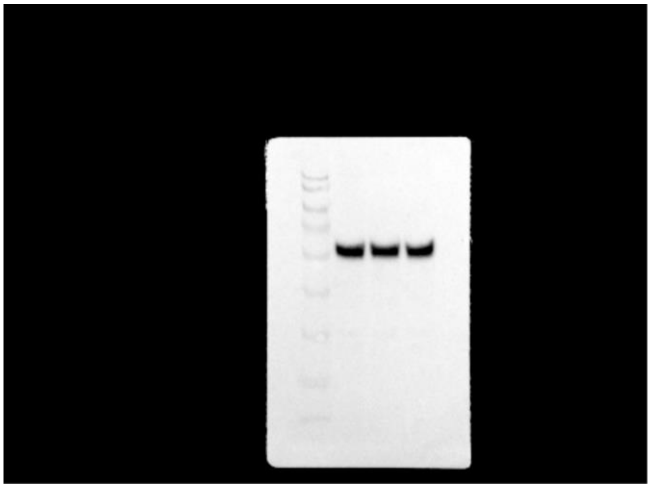

Figure 9C-6(1)

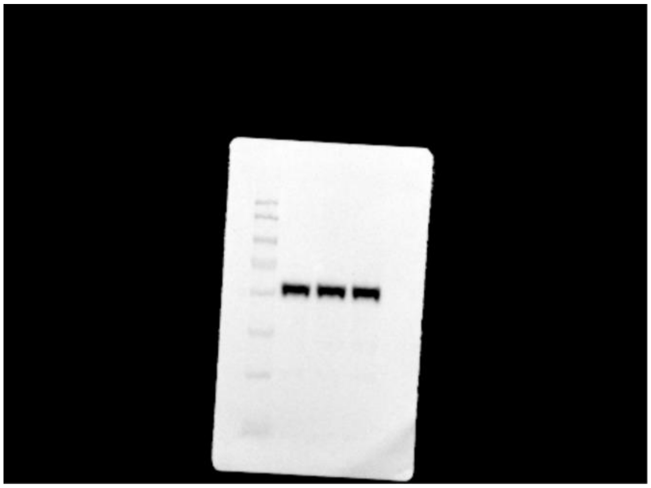

Figure 9C-6(2)

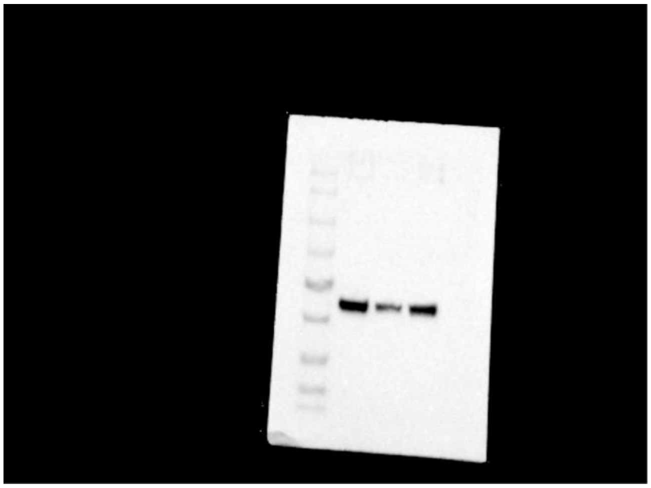

Figure 9C-7(1)

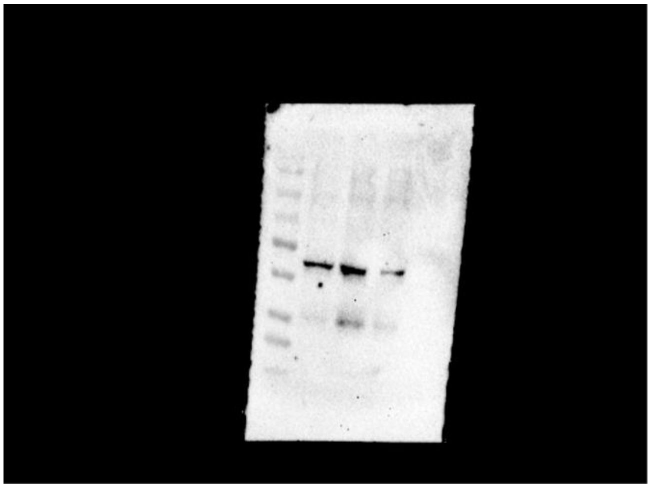

Figure 9C-7(2)

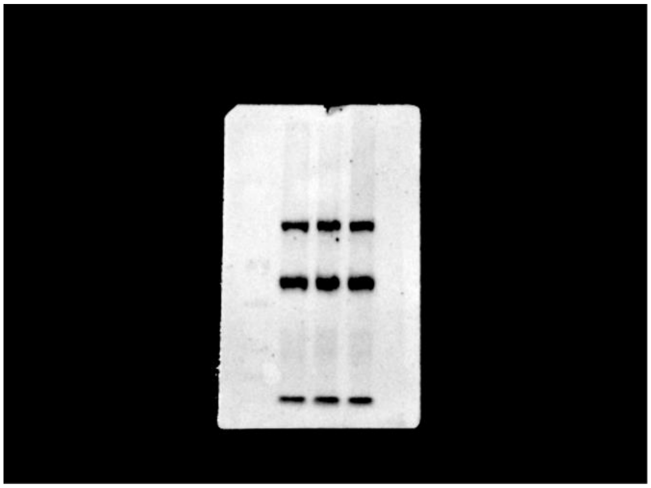

Figure 9C-8(1)

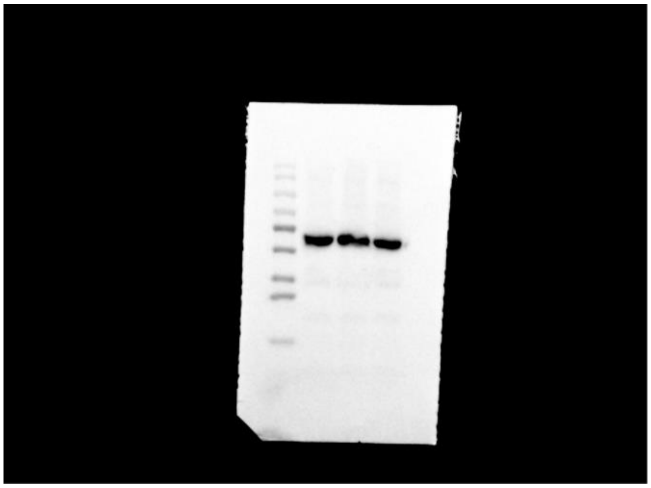

Figure 9C-8(2)

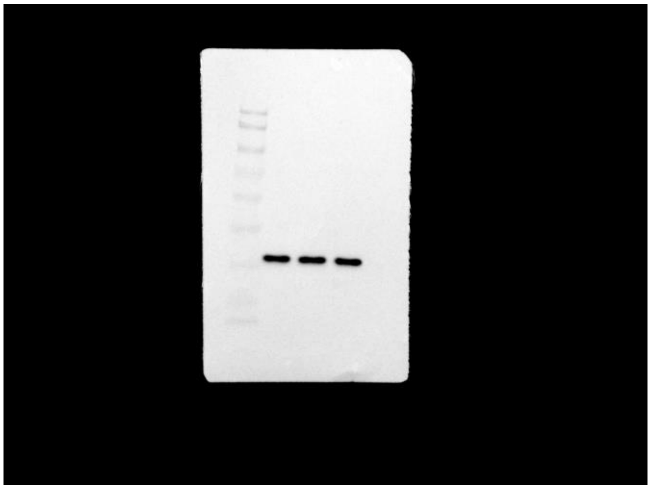

Figure 9C-9(1)

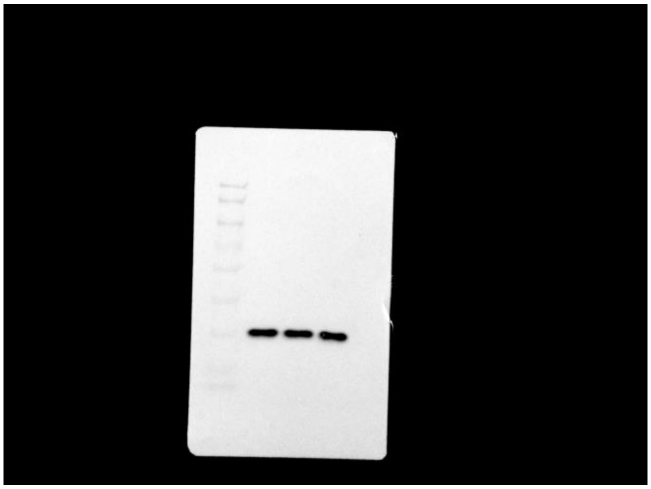

Figure 9C-9(2)

Figure 9D

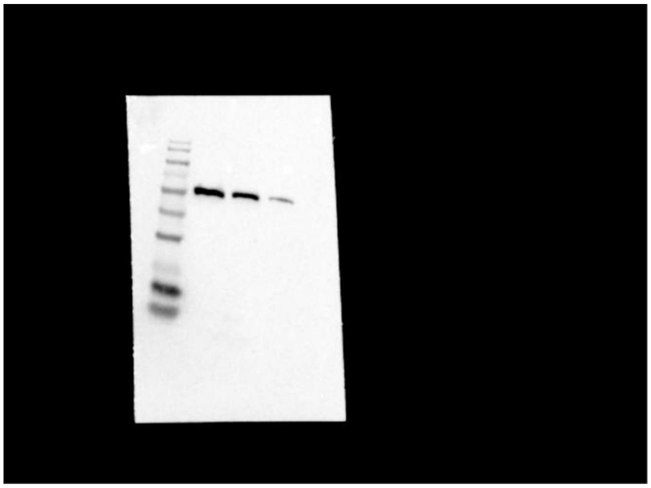

Figure 9D-1

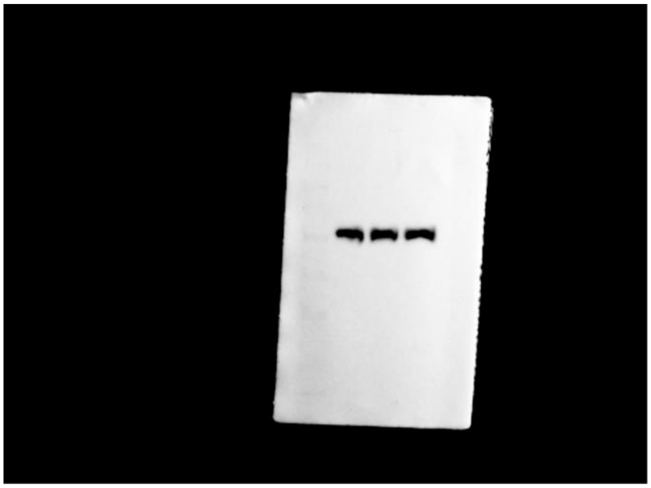

Figure 9D-2

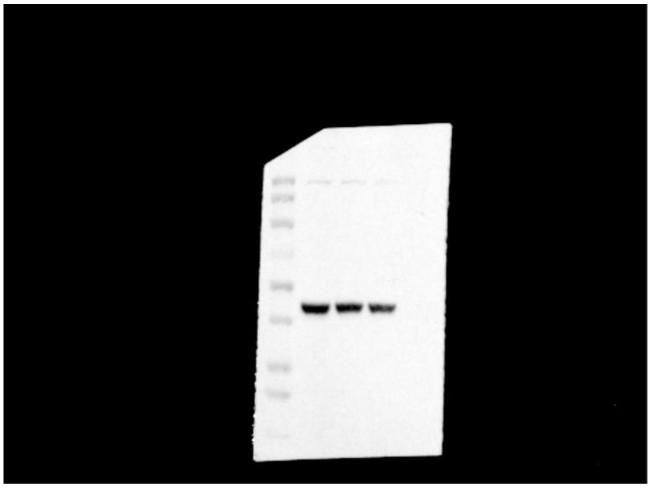

Figure 9D-3

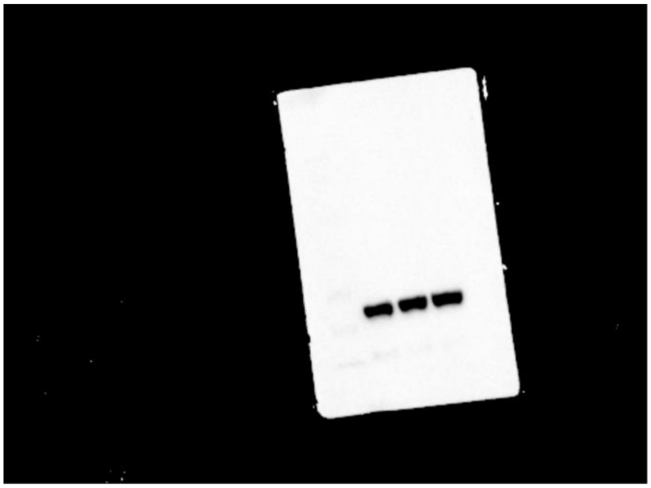

Figure 9D-4

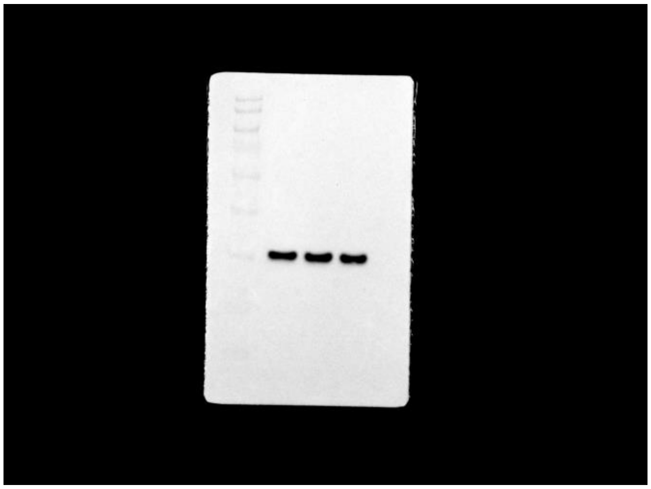

Figure 9D-5

Figure 9E

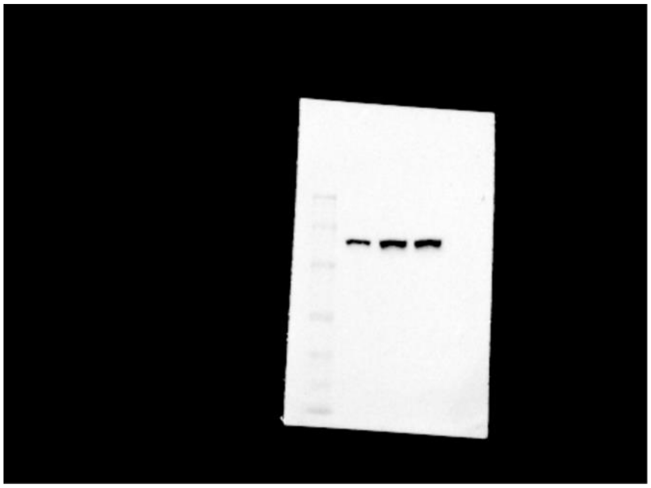

Figure 9E-1

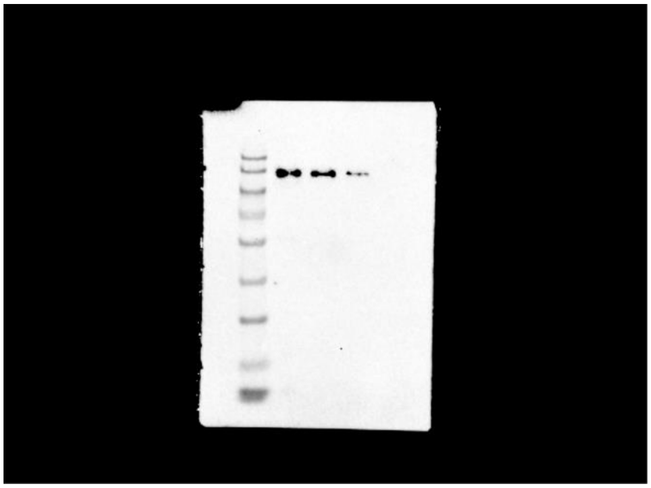

Figure 9E-2

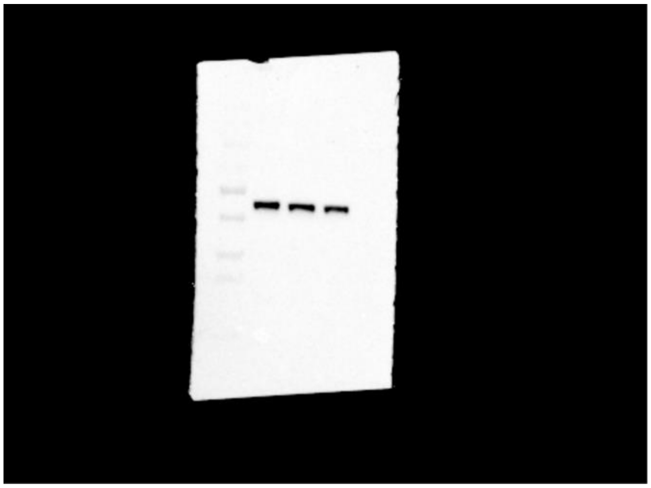

Figure 9E-3

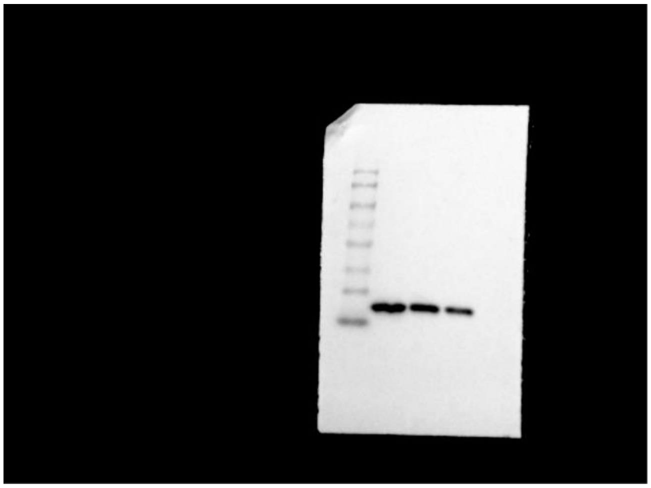

Figure 9E-4

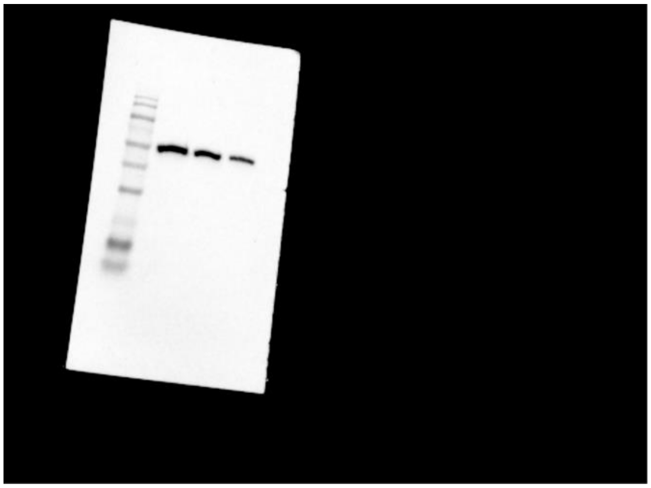

Figure 9E-5

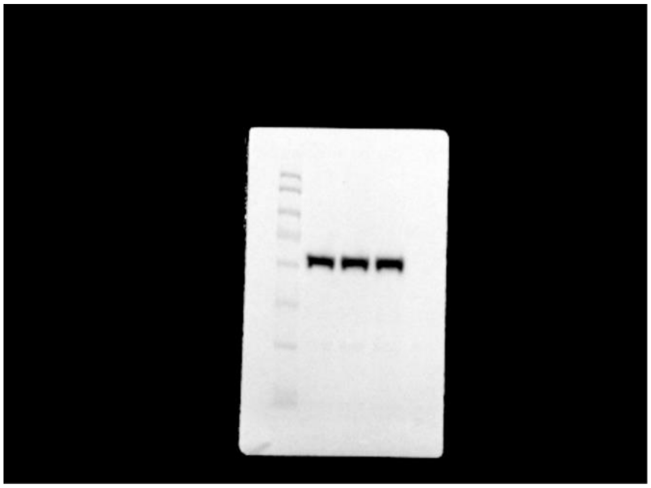

Figure 9E-6

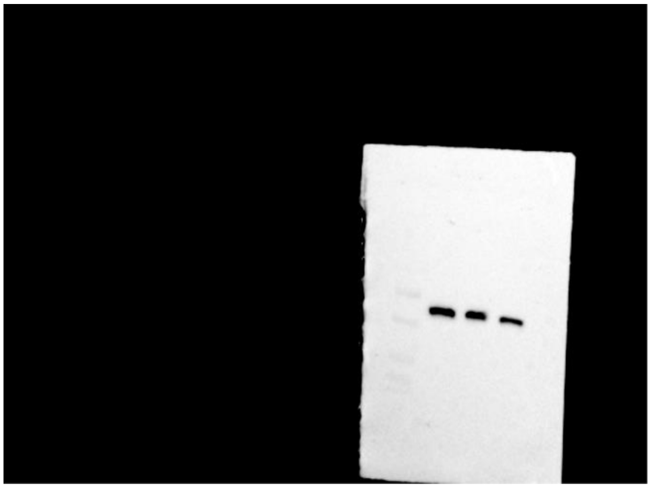

Figure 9E-7

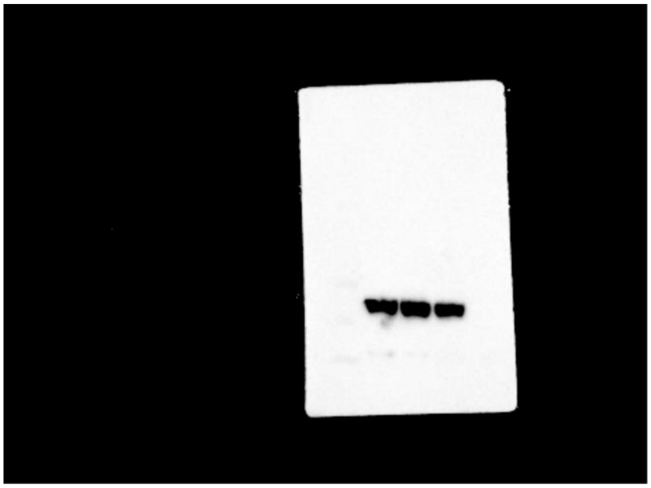

Figure 9E-8

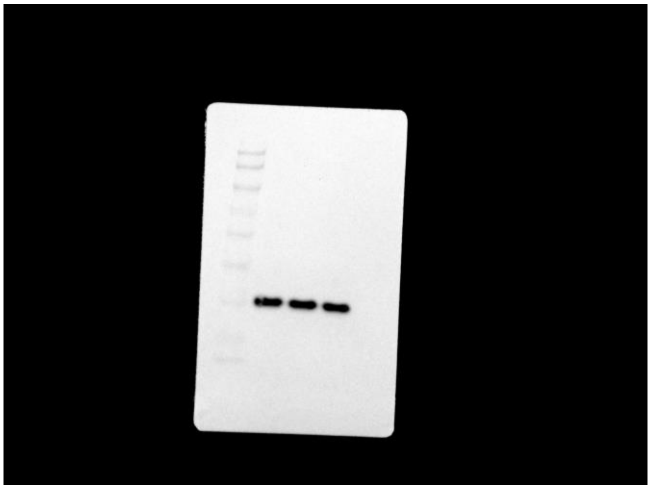

Figure 9E-9

Supplementary Figure S7

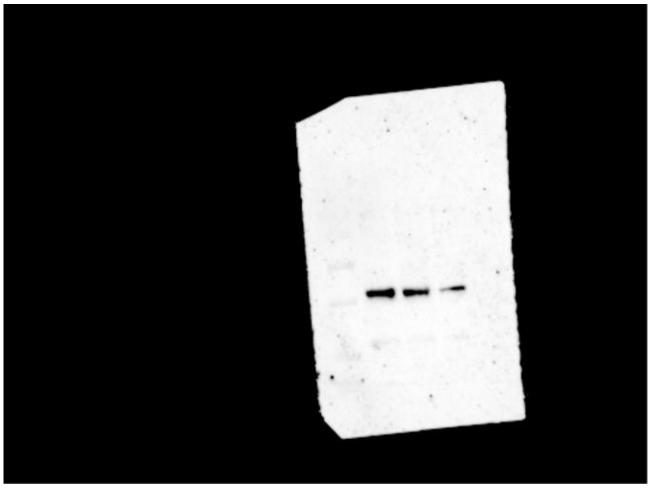

Figure S7_B_1_INHBA

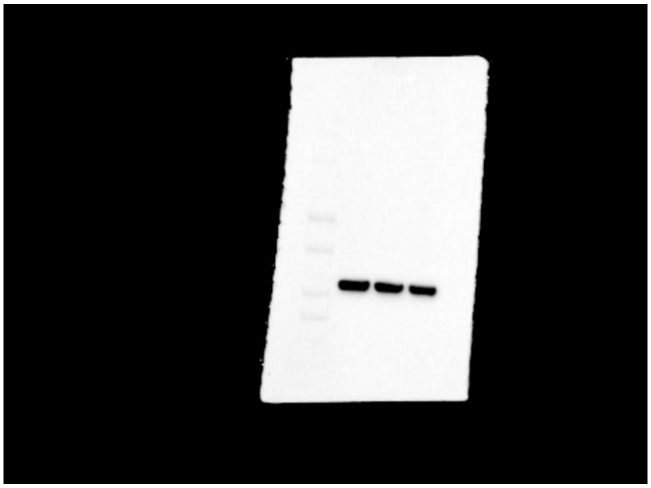

Figure S7_B_2_GAPDH

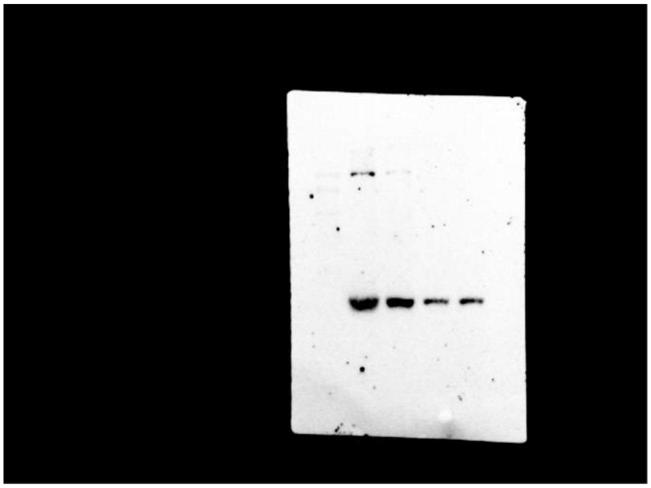

Figure S7_E_1_INHBA

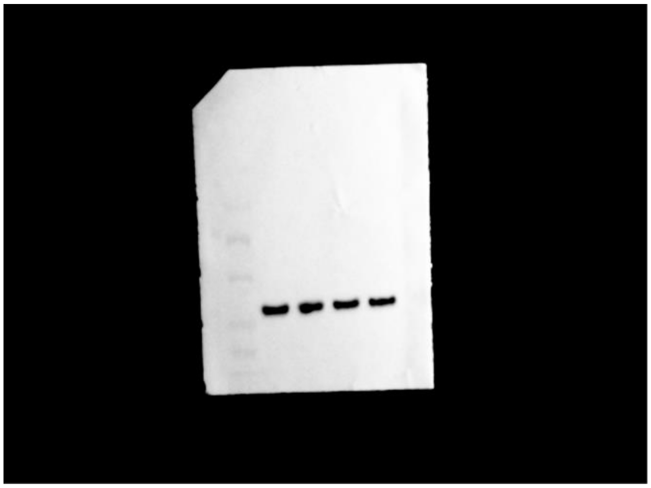

Figure S7_E_2_GAPDH

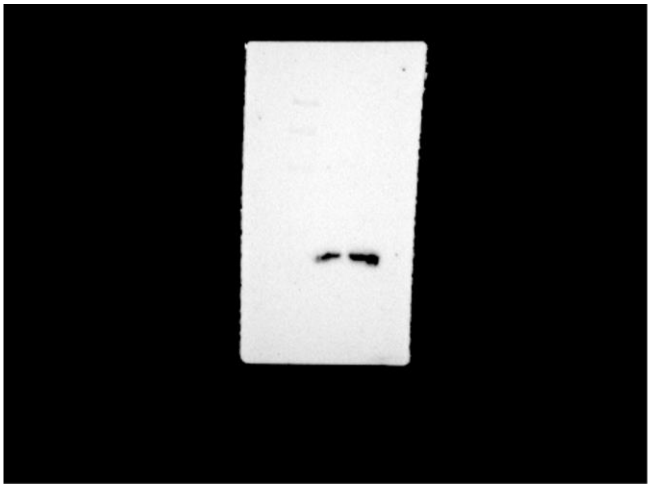

Figure S7_F_1_INHBA

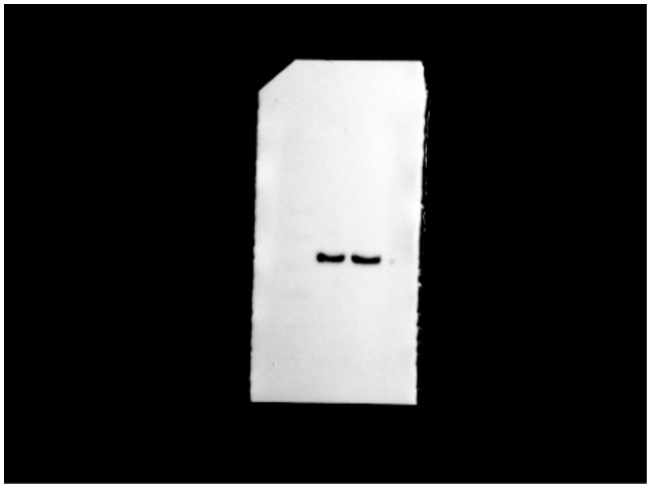

Figure S7_F_2_GAPDH
