# Supplementary material for: Construction of an Esophageal Cancer Prognostic Model Based on Mitochondrial Energy Metabolism-Related Genes and Mechanism of INHBA in Promoting Malignant Progression via the Smad2/3 Pathway
Source: Biomolecules. 2026 Sep 20;16(9):1367. doi: 10.3390/biom16091367 (PMC13604558; doi:10.3390/biom16091367)
Supplement: Supplementary file 1 [file biomolecules-16-01367-s001.zip › supplementary-figures and legend.pdf]

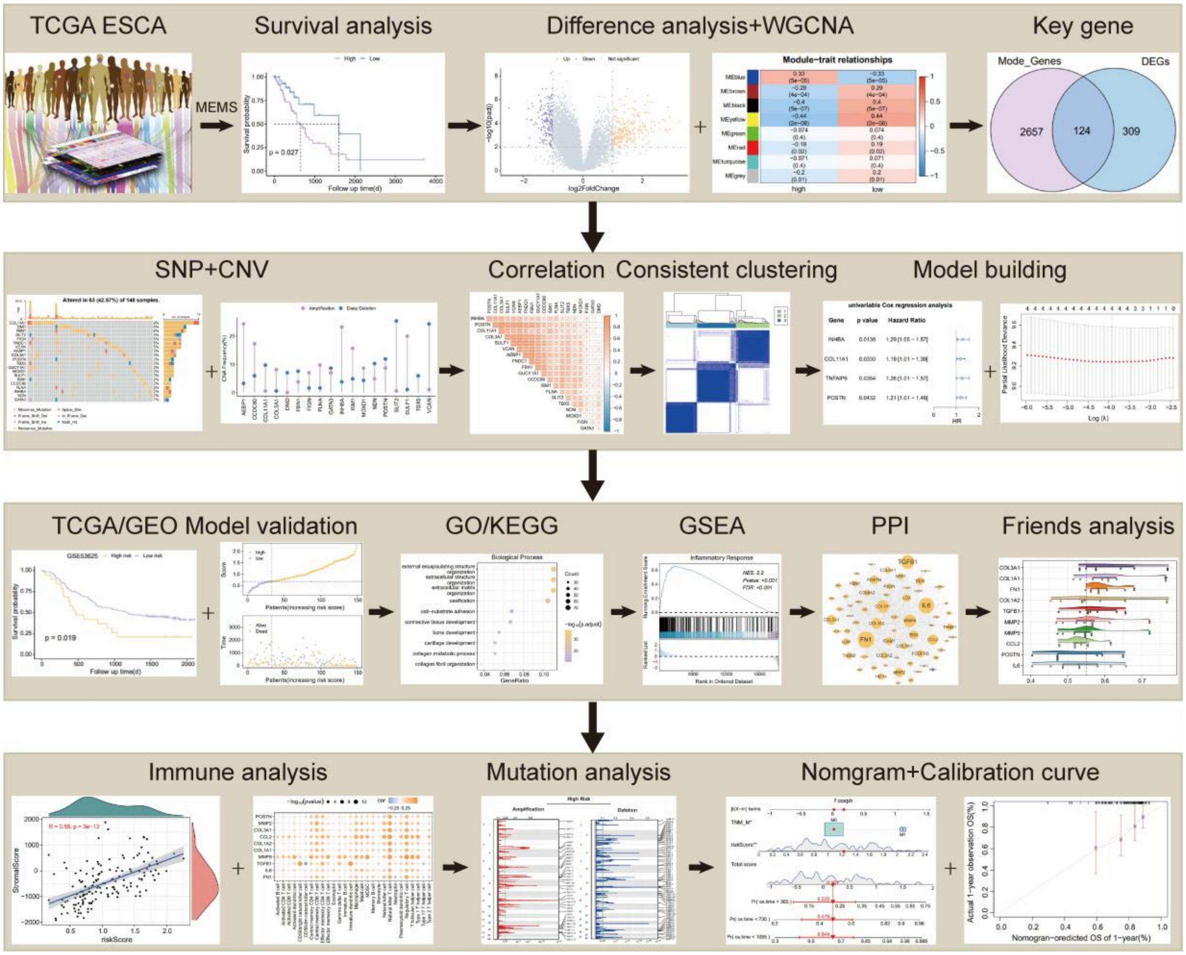

Figure S1. Overall workflow of the bioinformatics analyses in this study.

Note: This figure shows the overall technical route of this study, including data acquisition, MEMS calculation, differential expression analysis, WGCNA, molecular subtyping, prognostic model construction and validation, functional enrichment analysis, PPI network analysis, immune infiltration analysis, and clinical prediction model construction. TCGA: The Cancer Genome Atlas; ESCA: Esophageal Cancer; MEMS: Mitochondrial Energy Metabolism Score; WGCNA: Weighted Gene Co-Expression Network Analysis; CNV: Copy Number Variation; GEO: Gene Expression Omnibus; GO: Gene Ontology; KEGG: Kyoto Encyclopedia of Genes and Genomes; GSEA: Gene Set Enrichment Analysis; PPI: Protein-Protein Interaction.

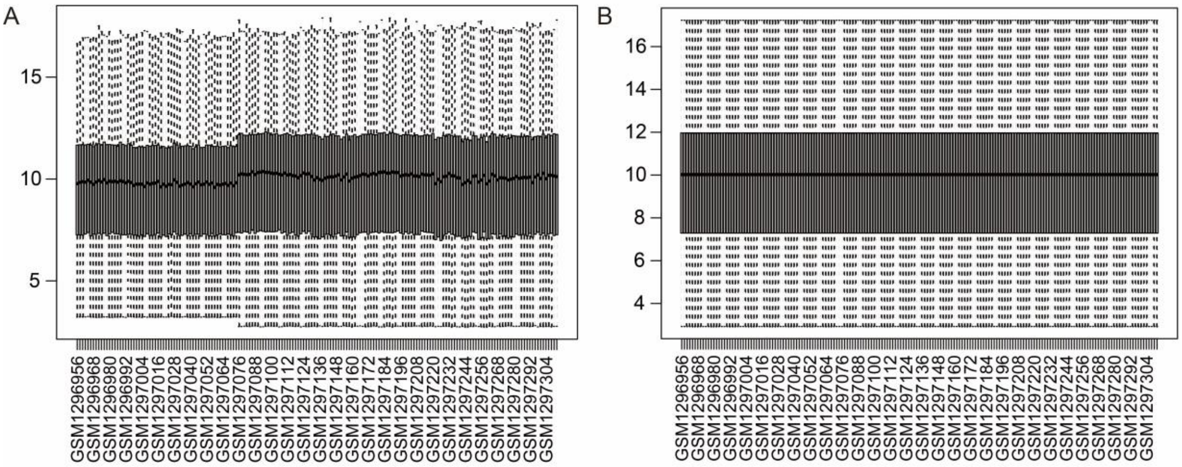

Figure S2. Boxplots of expression profiles before and after normalization correction in the GSE53625 dataset.

Note: (A) Boxplot of gene expression distributions before normalization correction in the

GSE53625 dataset; (B) Boxplot of gene expression distributions after normalization correction in the GSE53625 dataset. The x-axis represents sample IDs, and the y-axis represents gene expression values. After correction, median expression levels across samples are consistent, indicating that batch effects have been effectively removed.

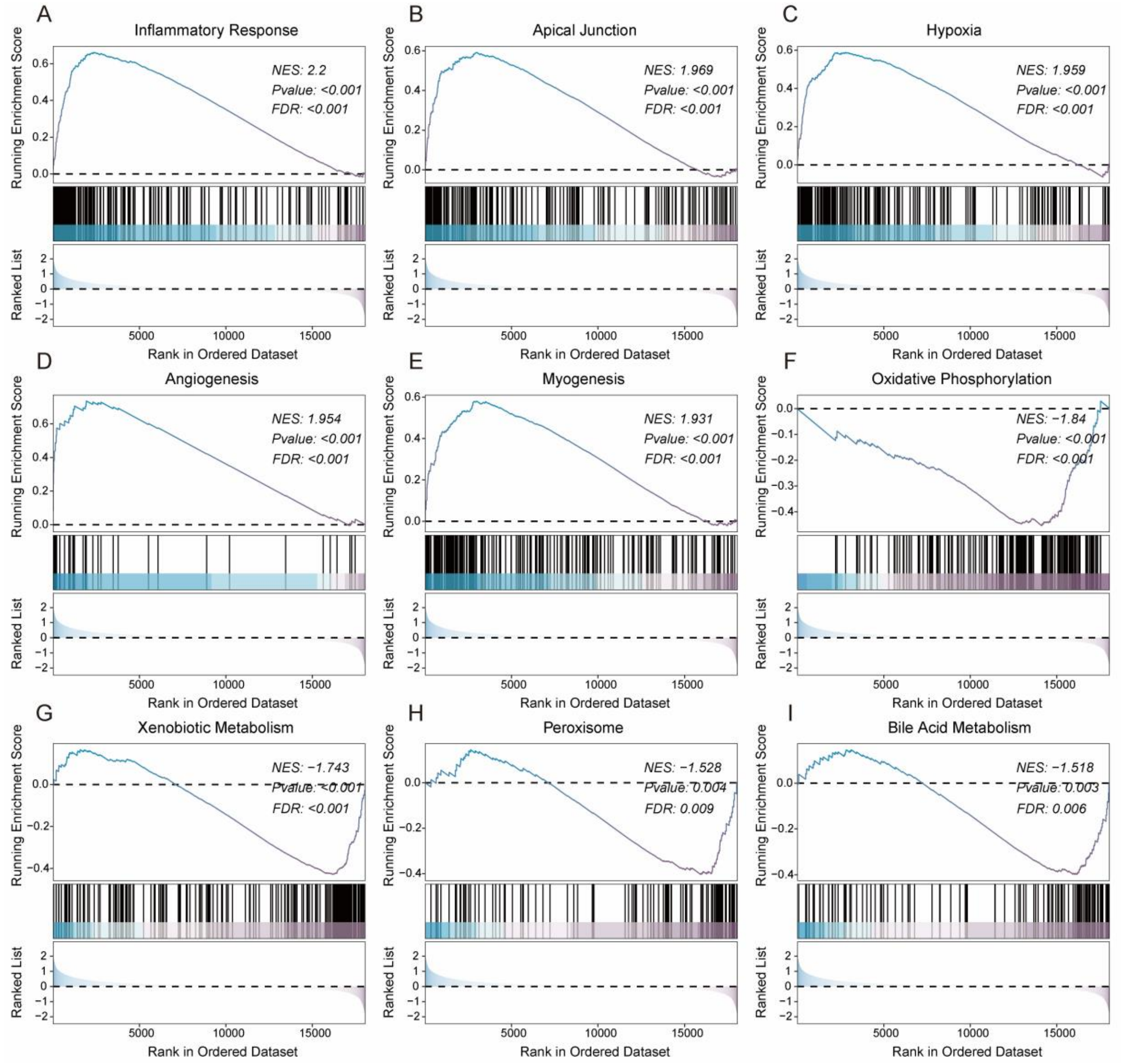

Figure S3. GSEA enrichment analysis plots based on the TCGA-ESCA dataset.

Note: (A-I) The GSEA enrichment curves for the Inflammatory Response, Apical Junction, Hypoxia, Angiogenesis, Myogenesis, Oxidative Phosphorylation, Xenobiotic Metabolism, Peroxisome, and Bile Acid Metabolism pathways, respectively. In each panel, the upper part shows the enrichment score curve, the vertical bars in the middle indicate the positions of the gene set members in the ranked gene list, and the lower part shows the distribution of the signal-to-noise ratio for gene ranking. NES > 0 indicates that the pathway is enriched in the high-risk group, whereas NES < 0 indicates that the pathway is enriched in the low-risk group. NES: Normalized Enrichment Score; FDR: False Discovery Rate; TCGA: The Cancer Genome Atlas; ESCA: Esophageal Cancer; GSEA: Gene Set Enrichment Analysis.

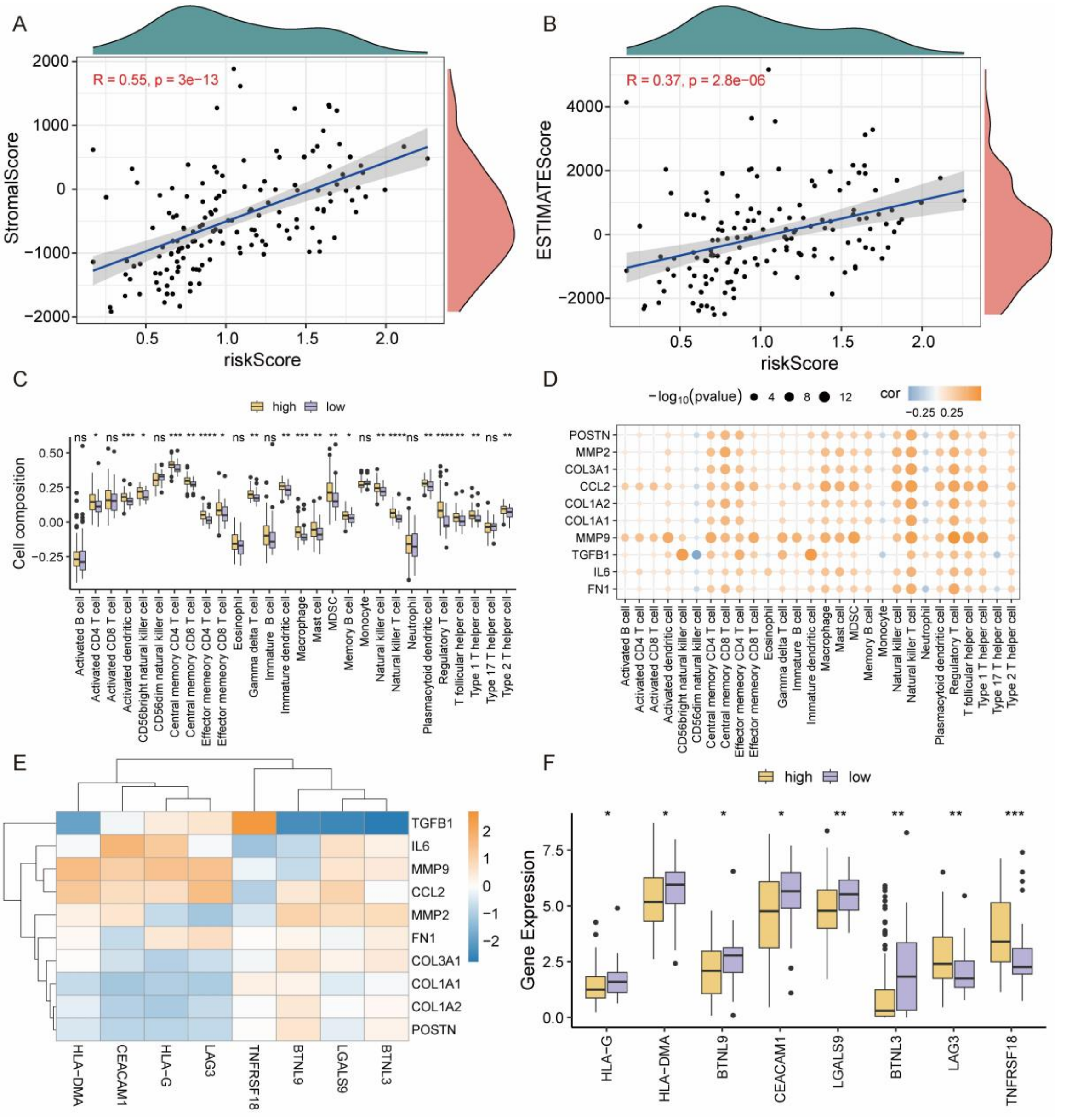

Figure S4. Immune infiltration analysis based on the TCGA-ESCA dataset.

Note: (A) Scatter plot showing the Pearson correlation between the risk score and Stromal Score; the blue fitted line indicates the linear regression trend, and the shaded area indicates the 95% confidence interval. (B) Scatter plot showing the Pearson correlation between the risk score and ESTIMATE Score; labeling is the same as in (A). (C) Box plots comparing infiltration levels of immune cell subtypes between the high- and low-risk groups; yellow indicates the high-risk group and blue indicates the low-risk group; between-group differences were assessed using the Wilcoxon rank-sum test. (D) Bubble plot of Pearson correlations between hub genes and immune cell subtype infiltration scores; bubble size

represents -$\log_{10}$(p-value), and color intensity represents the magnitude of the correlation coefficient; orange indicates positive correlation, and blue indicates negative correlation. (E) Heatmap of Pearson correlations between hub genes and expression levels of immune-related genes; colors from blue to red indicate correlation coefficients from negative to positive. (F) Box plots comparing expression of immune-related genes between the high- and low-risk groups; yellow indicates the high-risk group and blue indicates the low-risk group; between-group differences were assessed using the Wilcoxon rank-sum test. TCGA: The Cancer Genome Atlas; ESCA: Esophageal Cancer. *$p < 0.05$, **$p < 0.01$, ***$p < 0.001$; ns indicates no statistically significant difference.

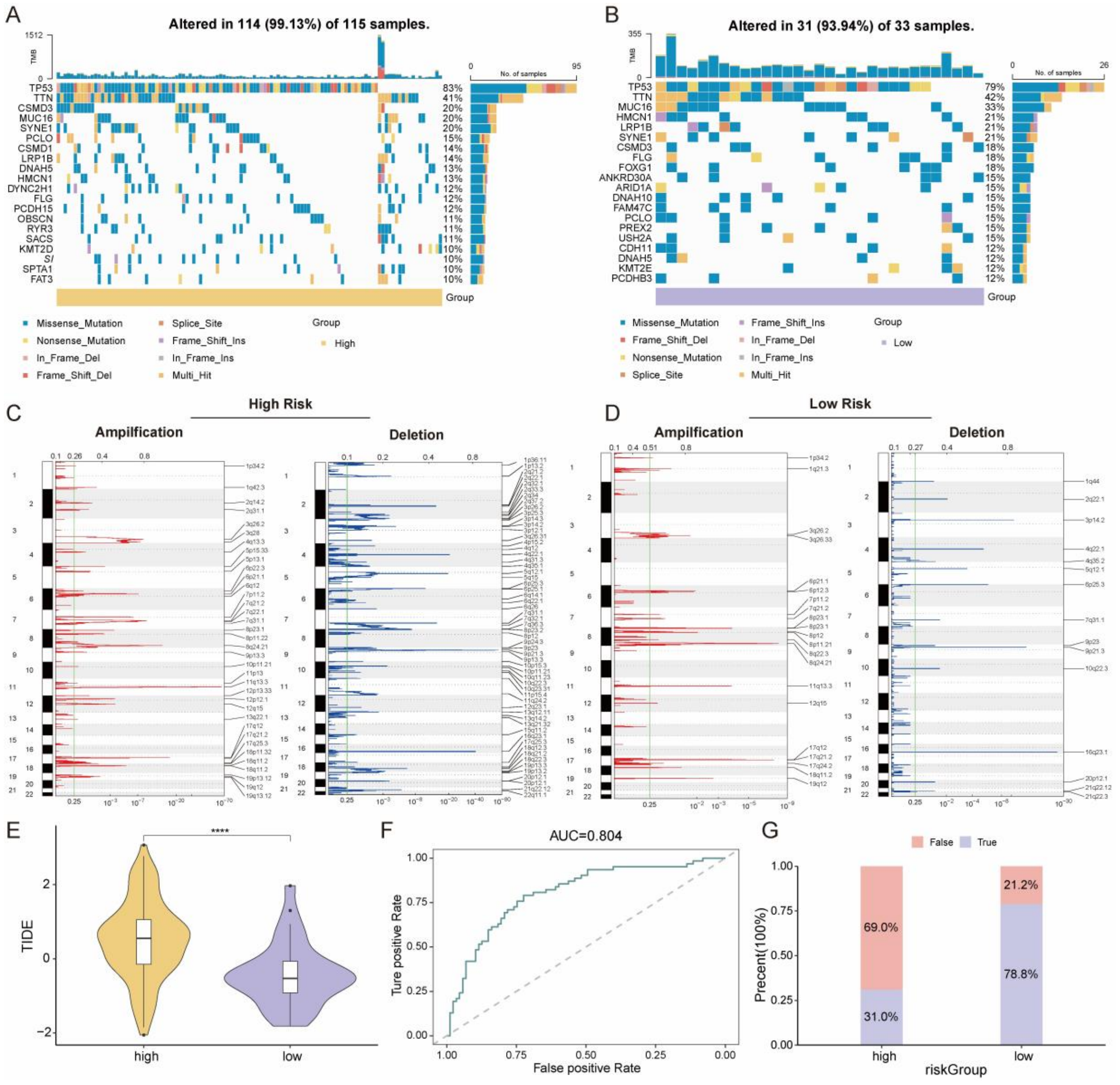

Figure S5. MEMS risk score and genomic alterations and immunotherapy response

assessment in TCGA-ESCA patients.

Note: (A) Waterfall plot of somatic mutations in common driver genes in the high-risk group. The bar plot at the top shows the mutation burden for each sample, and the percentages on the right indicate the mutation frequency for each gene; different colors represent different mutation types. (B) Waterfall plot of somatic mutations in common driver genes in the low-risk group, annotated as in (A). (C) CNV landscape from GISTIC 2.0 analysis in the high-risk group. The left panel shows copy-number amplification and the right panel shows copy-number deletion; the x-axis indicates the G-score and the y-axis indicates chromosomal locations. (D) CNV landscape from GISTIC 2.0 analysis in the low-risk group, annotated as in (C). (E) Violin plot comparing TIDE scores between high- and low-risk groups; yellow indicates the high-risk group and purple indicates the low-risk group. The Wilcoxon rank-sum test was used to assess between-group differences. (F) ROC curve for predicting immunotherapy response by the risk score; the green line is the ROC curve, AUC = 0.804. (G) Stacked bar chart showing the proportions of predicted immunotherapy response status in the high- and low-risk groups; red indicates predicted non-response (False) and blue indicates predicted response (True). TCGA, The Cancer Genome Atlas; ESCA, Esophageal Cancer; TIDE, Tumor Immune Dysfunction and Exclusion; ROC, Receiver Operating Characteristic; AUC, Area Under the Curve. ***$p < 0.001$.

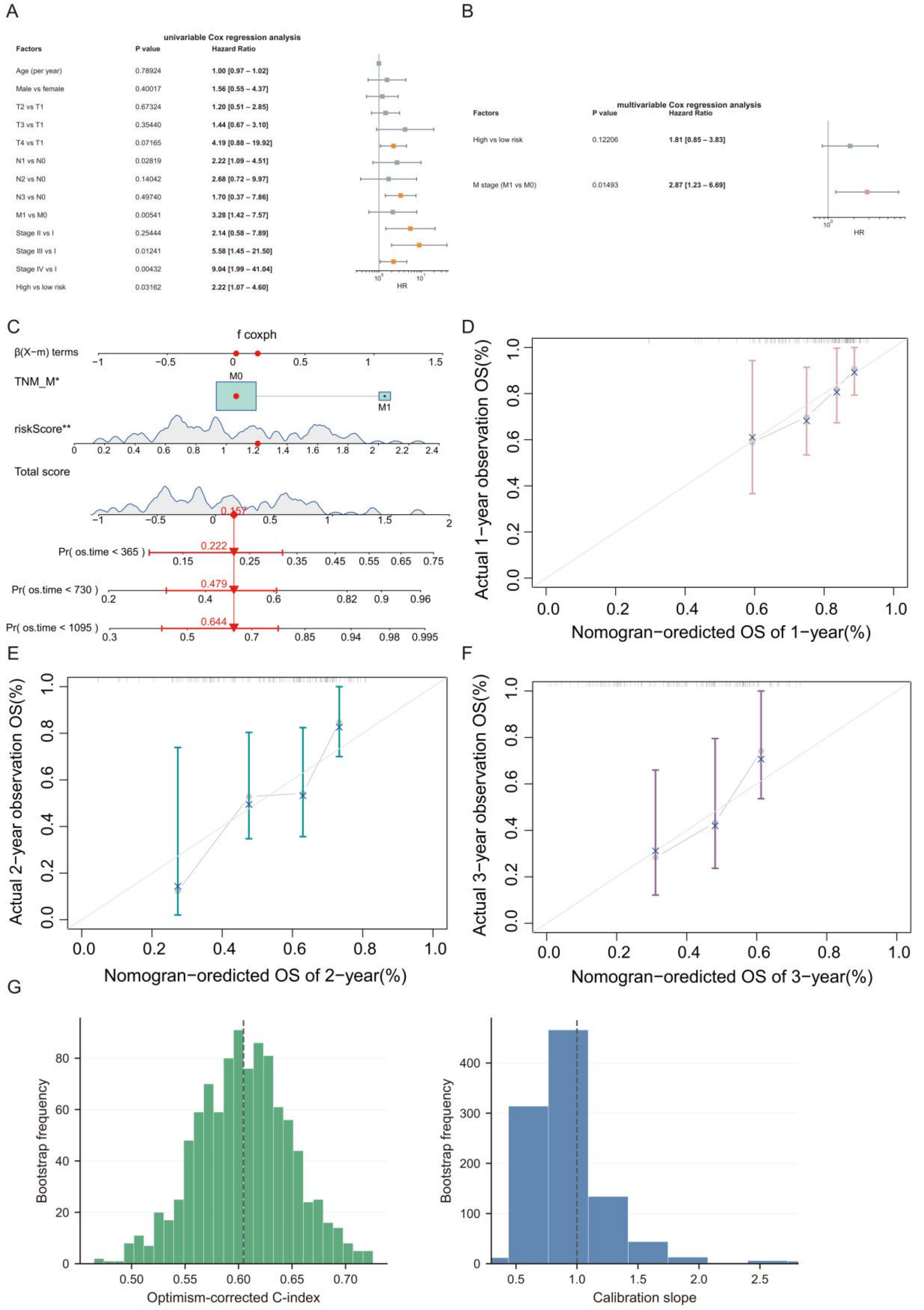

Figure S6. Construction and evaluation of a risk score-based clinical prediction model**.**

Note: (A) Univariate Cox regression forest plot showing HRs and 95% CIs using variable-

specific complete cases (n/events: age, sex, and risk group, 149/48; T stage, 148/47; N stage, 145/47; M stage, 127/42; overall stage, 135/44); orange indicates $p < 0.05$ and gray indicates $p \geq 0.05$. (B) Multivariable Cox regression forest plot for risk group and M stage in 127 complete cases with 42 deaths. (C) Nomogram constructed by integrating the risk score and M stage; each variable corresponds to a score, and the total score is used to predict 1-, 2-, and 3-year survival probabilities. (D) Calibration curve for 1-year OS. (E) Calibration curve for 2-year OS. (F) Calibration curve for 3-year OS. (G) Distributions of the optimism-corrected Harrell C-index and bootstrap calibration slope from 1,000 resamples of the final two-variable Cox model. In the calibration curves, the x-axis represents the survival probability predicted by the nomogram, and the y-axis represents the actually observed survival probability; the diagonal dashed line indicates ideal prediction, and calibration points closer to the diagonal indicate higher predictive accuracy. TCGA: The Cancer Genome Atlas; ESCA: Esophageal Cancer; OS: Overall Survival; HR: Hazard Ratio.

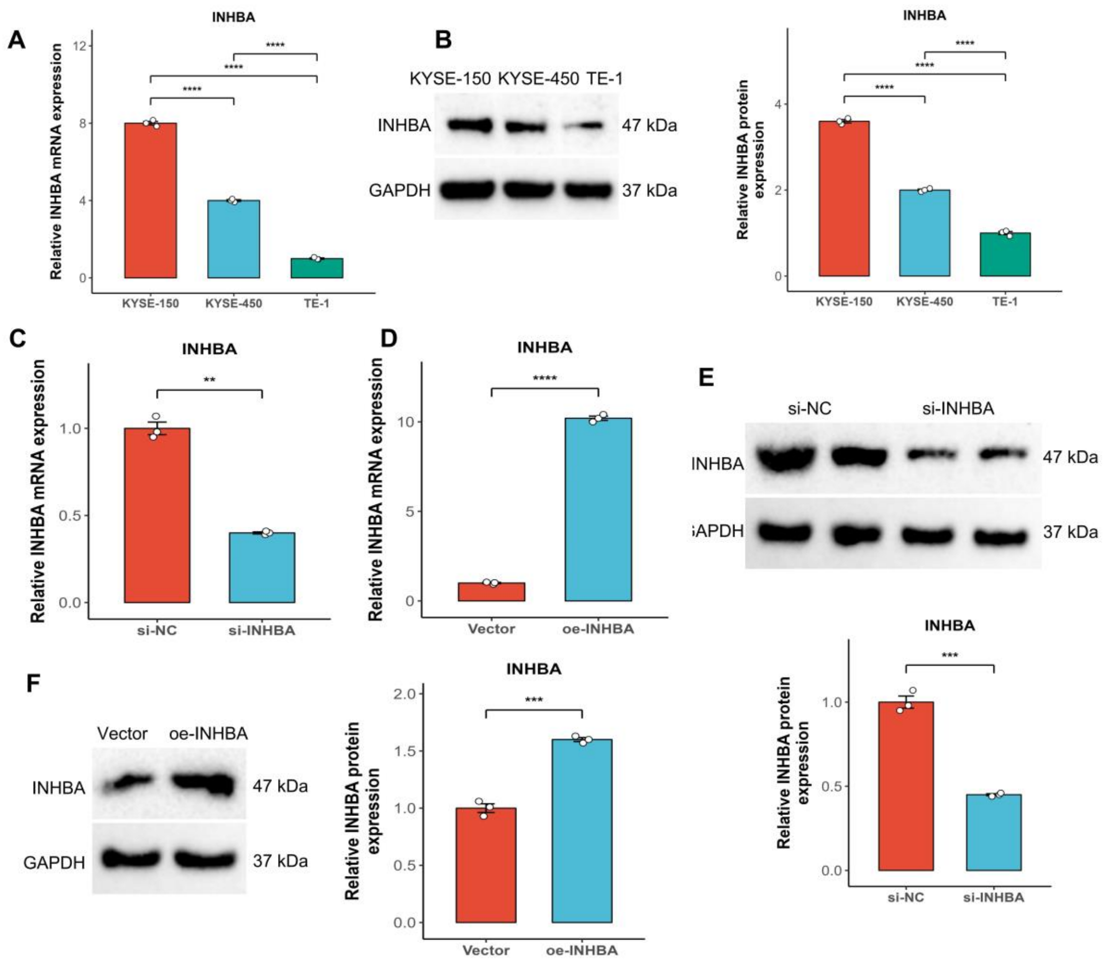

Figure S7. INHBA expression levels in esophageal squamous cell carcinoma cells and construction of intervention models.

Note: (A) qRT-PCR was used to detect the relative INHBA mRNA expression levels in KYSE-150, KYSE-450, and TE-1 cells; (B) WB was used to detect INHBA protein expression levels in different esophageal squamous cell carcinoma cells (left: representative bands; right: grayscale densitometric analysis); (C) Verification of INHBA mRNA expression in KYSE-150 cells after si-INHBA transfection; (D) Verification of INHBA mRNA expression in TE-1 cells after oe-INHBA transfection; (E) Verification of INHBA protein expression in KYSE-150 cells after si-INHBA transfection (left: representative bands; right: grayscale densitometric analysis); (F) Verification of INHBA protein expression in TE-1 cells after oe-INHBA transfection (left: representative bands; right: grayscale densitometric analysis). qRT-PCR results were calculated using the $2^{-\Delta\Delta Ct}$ method, with GAPDH as the

internal reference gene; WB densitometric values were normalized to GAPDH. Data are presented as x̄ ± s, and experiments were independently repeated three times. **$p < 0.01$, ***$p < 0.001$, ****$p < 0.0001$.

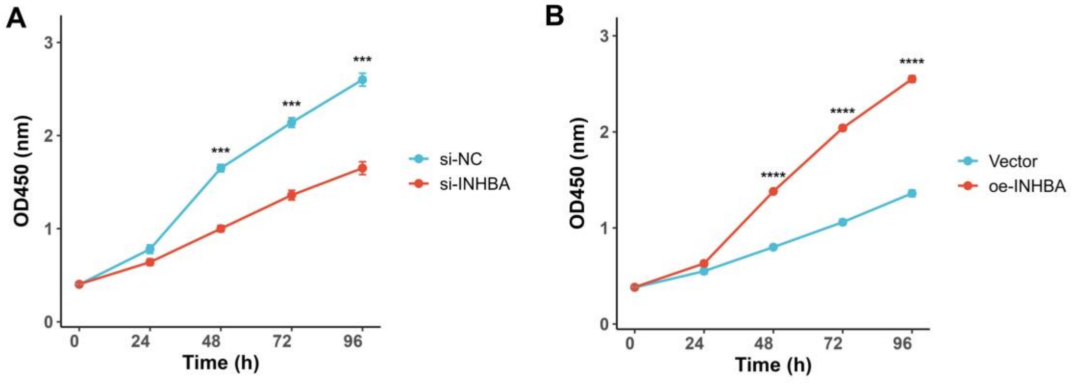

Figure S8. Effects of INHBA on the short-term proliferative capacity of esophageal squamous cell carcinoma cells.

Note: (A) CCK-8 growth curve of KYSE-150 cells after transfection with si-INHBA; blue indicates the si-NC control group, and orange indicates the si-INHBA group. (B) CCK-8 growth curve of TE-1 cells after transfection with oe-INHBA; blue indicates the empty vector control group (Vector), and orange indicates the oe-INHBA group. OD_{450} values were measured at 0, 24, 48, 72, and 96 h. Data are presented as x̄ ± s; experiments were independently repeated three times. **$p < 0.01$, ***$p < 0.001$, ****$p < 0.0001$.

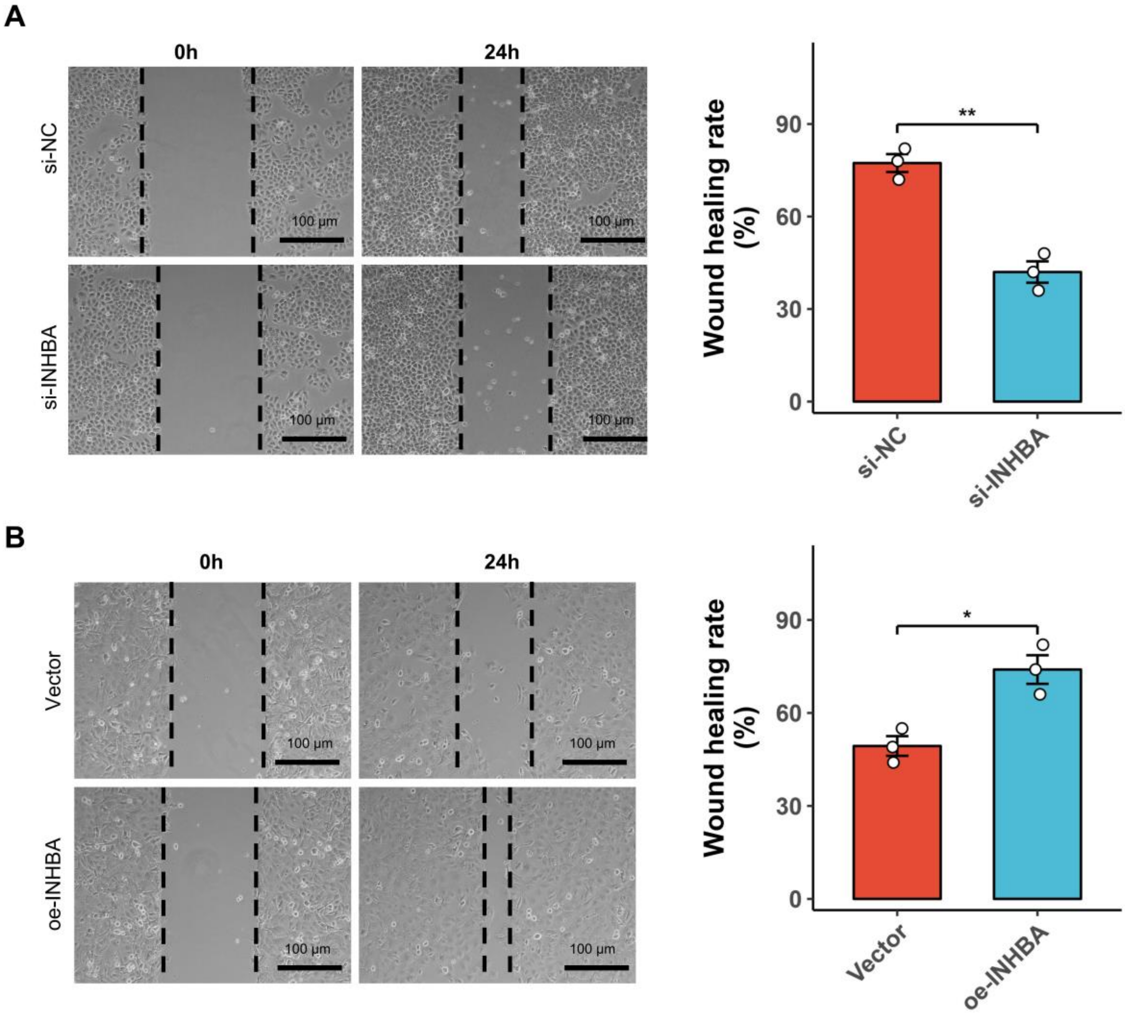

Figure S9. Effects of INHBA on the wound-healing migration ability of esophageal squamous cell carcinoma cells.

Note: (A) Representative images (left: 0 h and 24 h) and statistical analysis of the wound-healing rate (right) of KYSE-150 cells after si-INHBA transfection; (B) Representative images (left: 0 h and 24 h) and statistical analysis of the wound-healing rate (right) of TE-1 cells after oe-INHBA transfection. Wound-healing rate = (wound width at 0 h - wound width at 24 h)/wound width at 0 h × 100%. Data are presented as $\bar{x} \pm s$, and experiments were independently repeated three times. *$p < 0.05$, **$p < 0.01$.

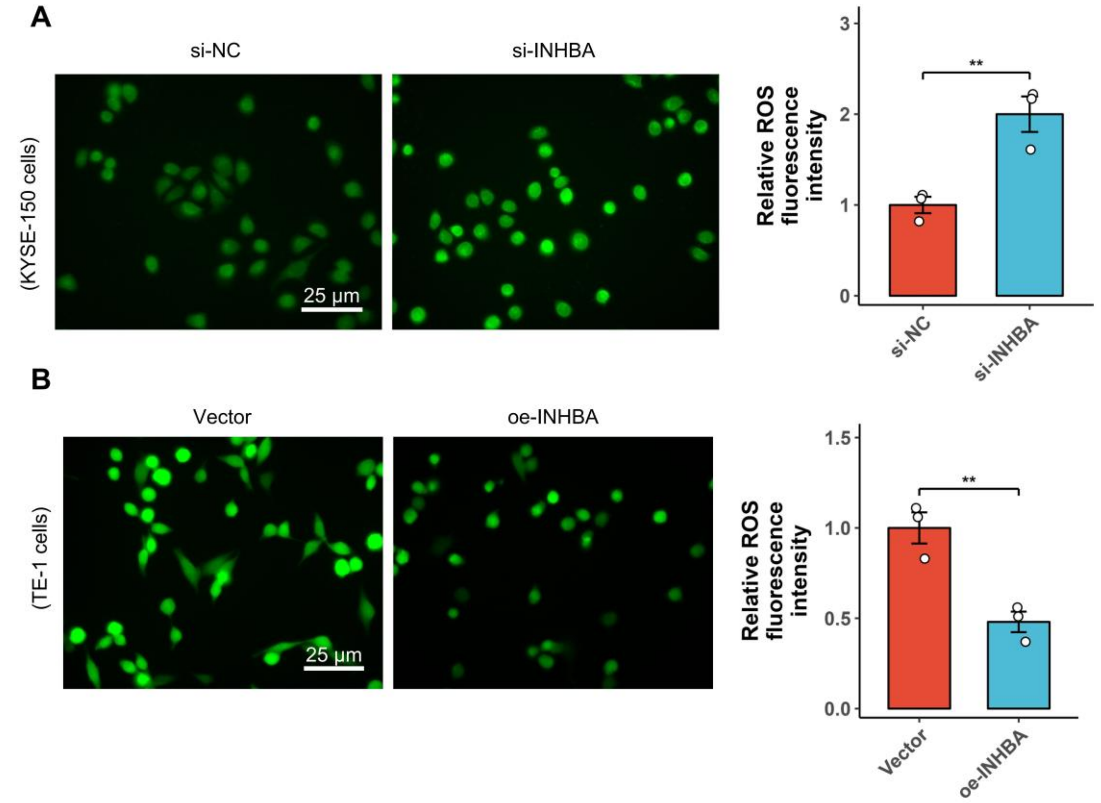

Figure S10. Effects of INHBA on oxidative stress levels in esophageal squamous cell carcinoma cells.

Note: (A) Representative images (left) and quantification (right) of DCFH-DA fluorescence in KYSE-150 cells after si-INHBA transfection (scale bar: 25 μm); (B) Representative images (left) and quantification (right) of DCFH-DA fluorescence in TE-1 cells after oe-INHBA transfection (scale bar: 25 μm). ROS levels are expressed as relative DCFH-DA fluorescence intensity. Data are presented as $\bar{x} \pm s$, with experiments independently repeated three times. $^{**}p < 0.01$.

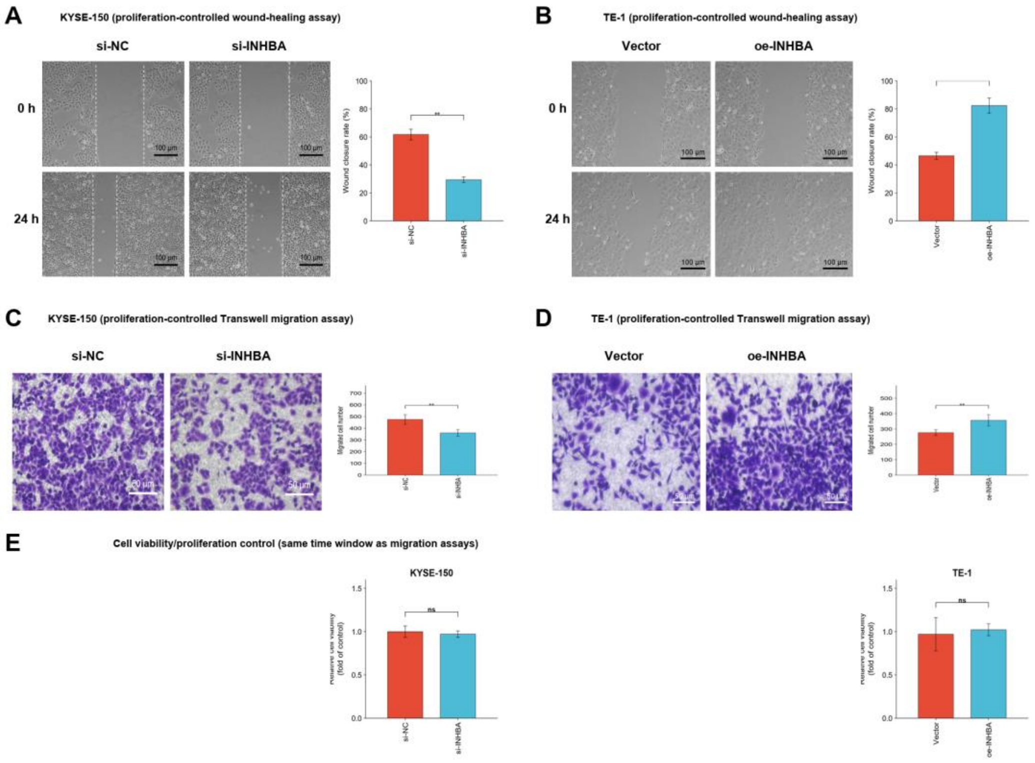

Figure S11. Proliferation-controlled migration assays validate the effect of INHBA on esophageal squamous cell carcinoma cell motility.

Note: (A) Representative wound-healing images and wound closure rate in KYSE-150 cells under proliferation-controlled conditions after si-INHBA transfection; (B) representative wound-healing images and wound closure rate in TE-1 cells under proliferation-controlled conditions after oe-INHBA transfection; (C) representative Transwell migration images and migrated-cell quantification in KYSE-150 cells under proliferation-controlled conditions after si-INHBA transfection; (D) representative Transwell migration images and migrated-cell quantification in TE-1 cells under proliferation-controlled conditions after oe-INHBA transfection; (E) parallel cell-viability/proliferation monitoring during the migration assay window. Data are presented as mean ± SD from n = 3 independent experiments. *$p < 0.05$, **$p < 0.01$; ns indicates no statistically significant difference.

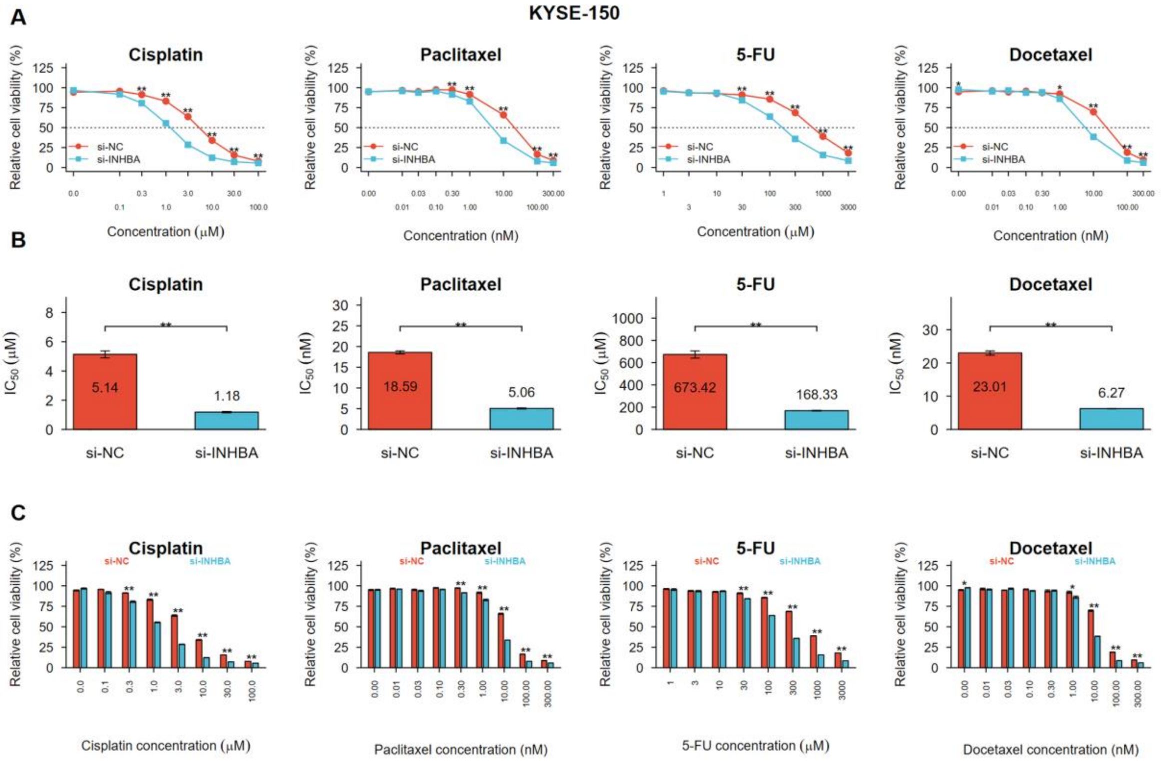

Figure S12. INHBA knockdown alters chemotherapy sensitivity in esophageal cancer cells. Note: (A) Dose-response curves of si-NC and si-INHBA-transfected esophageal cancer cells treated with Cisplatin, Paclitaxel, 5-FU, or Docetaxel; (B) IC_{50} values calculated from nonlinear dose-response fitting. IC_{50} values decreased from 5.23 to 1.21 μM for Cisplatin, from 18.6 to 5.12 nM for Paclitaxel, from 642.3 to 172.4 μM for 5-FU, and from 22.7 to 6.38 nM for Docetaxel after INHBA knockdown; (C) cell-viability summary at each drug concentration. Data are presented as mean ± SD from n = 3 independent experiments. *$p < 0.05$, **$p < 0.01$ (si-NC vs si-INHBA).
